# An integrated transcriptomic and proteomic approach to identify the main *Torymus sinensis* venom components

**DOI:** 10.1038/s41598-021-84385-5

**Published:** 2021-03-03

**Authors:** Carmen Scieuzo, Rosanna Salvia, Antonio Franco, Marco Pezzi, Flora Cozzolino, Milvia Chicca, Chiara Scapoli, Heiko Vogel, Maria Monti, Chiara Ferracini, Pietro Pucci, Alberto Alma, Patrizia Falabella

**Affiliations:** 1 Department of Sciences, University of Basilicata, Via dell’Ateneo Lucano 10, 85100 Potenza, Italy; 2 Spinoff XFlies S.R.L, University of Basilicata, Via dell’Ateneo Lucano 10, 85100 Potenza, Italy; 3 Department of Life Sciences and Biotechnology, University of Ferrara, Via L. Borsari 46, 44121 Ferrara, Italy; 4 Department of Chemical Sciences, University Federico II of Napoli, Via Cinthia 6, 80126 Naples, Italy; 5 CEINGE Advanced Biotechnology, Via Gaetano Salvatore 486, 80126 Naples, Italy; 6 Department of Entomology, Max Planck Institute for Chemical Ecology, Hans-Knöll-Straße 8, 07745 Jena, Germany; 7 Department of Agricultural, Forest and Food Sciences, University of Torino, Largo Paolo Braccini 2, 10095 Grugliasco, Italy

**Keywords:** Proteomics, Sequencing, Entomology

## Abstract

During oviposition, ectoparasitoid wasps not only inject their eggs but also a complex mixture of proteins and peptides (venom) in order to regulate the host physiology to benefit their progeny. Although several endoparasitoid venom proteins have been identified, little is known about the components of ectoparasitoid venom. To characterize the protein composition of *Torymus sinensis* Kamijo (Hymenoptera: Torymidae) venom, we used an integrated transcriptomic and proteomic approach and identified 143 venom proteins. Moreover, focusing on venom gland transcriptome, we selected additional 52 transcripts encoding putative venom proteins. As in other parasitoid venoms, hydrolases, including proteases, phosphatases, esterases, and nucleases, constitute the most abundant families in *T. sinensis* venom*,* followed by protease inhibitors. These proteins are potentially involved in the complex parasitic syndrome, with different effects on the immune system, physiological processes and development of the host, and contribute to provide nutrients to the parasitoid progeny. Although additional in vivo studies are needed, initial findings offer important information about venom factors and their putative host effects, which are essential to ensure the success of parasitism.

## Introduction

Insects are characterized by the highest level of biodiversity of all organisms, with around 1 million described species^1,2^. A molecular and functional understanding of major associations between insects and other organisms may result in the identification of useful genes and molecules for the development of new strategies to control harmful insects^3,4^, and also for biomedical and industrial applications through biotechnologies^5,6^. Phytophagous insects are responsible for massive negative impacts on agricultural production, inducing directly and indirectly plant damage, i.e. through the transmission of plant pathogens^7^. However, insects can be attacked by a wide range of natural antagonists, which are commonly known as entomophages and categorized as predators or parasitoids^8^. Predators attack and consume their prey directly and immediately^8^. Parasitoids, instead, preserve the victim for their own progeny by injecting secretions into the hosts that can regulate their physiology, transforming the host into a suitable environment for the development of the new-born larvae^9^. Hymenopteran parasitoids show the most sophisticated strategies. Whereas ectoparasitoid wasps lay their eggs on the surface of hosts or in the environment close to the host, and their larvae develop outside the host body, endoparasitoid wasps oviposit and their progeny develop inside the host hemocoel^10^.

The main alterations observed in the parasitized host are induced by secretions (venom and ovarian calyx fluid) injected by the adult female during oviposition^10^. Venom of parasitoid insects is produced by glands attached to the female reproductive system and consists of a complex mixture of proteins, peptides, and some unidentified compounds^11^. Bioactive components in venom are responsible for alterations of host development and metabolism, in order to optimize nutrient supply for parasitoid offspring^12^. Unlike endoparasitoids whose venom can induce various effects ranging from the regulation/alteration of host physiology^13,14^ to transiently paralyzing even lethal effects^15,16^, ectoparasitoid venom paralyze or rapidly kill the host^12,17^. To better understand the pathological syndrome observed in parasitized hosts, including alterations of host physiology, development, and reproduction, it is essential to identify and characterize the components of venom and ovarian fluid.

To date, several venom proteins have been identified both in endo- and ectoparasitoid wasps using different approaches^18–20^. Only after candidate venom protein identification, they can be functionally characterized to understand how they alter the host physiological processes. These alterations, in combination with other parasitic factors, induce changes in the immune system, both humoral (suppression of melanization processes) and cellular (inhibition of the encapsulation of foreign bodies by the hemocytes), in reproductive processes and in the digestive system (host tissues provide suitable nutrients for the parasitoid offspring)^13,21–23^. Moreover, some of the venom protein components can also play a paralyzing role, preserving the host tissues in response to the nutritional needs of the parasitoid progeny^24,25^.

Although several venom proteins from endoparasitoids have been identified^18,26–29^, proteins from ectoparasitoid venom are still largely unknown^20,30–32^. The most reliable strategy for the identification of these proteins consists of an integrated bottom-up proteomic and transcriptomic approach, using a combination of high-throughput next-generation transcriptome sequencing and mass spectrometry^18,20,22,26,28,30,32,33^.

*Torymus sinensis* Kamijo (Hymenoptera: Torymidae), is the parasitoid of the Asian chestnut gall wasp, *Dryocosmus kuriphilus* Yasumatsu (Hymenoptera: Cynipidae), a globally invasive pest of *Castanea* species. *T. sinensis*, a univoltine ectoparasitoid, is considered as the main biocontrol agent of *D. kuriphilus,* and its biological cycle is perfectly synchronized with its host^34,35^. The adult female inserts its ovipositor in the newly formed galls of *D. kuriphilus* and lays eggs in the inner wall of the gall or on the surface of the *D. kuriphilus* larva (Supplementary Fig. S1). Adults of *T. sinensis* emerge from the gall in early spring and mate, starting the biological cycle again^34^; the lack of a host may cause up to a 12-month diapause^36^. For these reasons, *T. sinensis,* native to China, has been introduced into several countries of Asia, North America and Europe to control populations of Asian chestnut gall wasps^37–40^.

Here, we employed an effective approach that combined next-generation transcriptome sequencing and proteomics to identify the major protein components of *T. sinensis* venom. The transcriptome of the *T. sinensis* venom gland was built by using a high-throughput nucleic acid sequencing method. Transcriptomic information provided an overall picture of the putative proteins of the venom gland and on their molecular functions, biological processes, and putative cellular compartments. Proteomic analysis was carried out on the components of the venom, fractionated by SDS-PAGE electrophoresis, and analyzed by mass spectrometry (nanoLC-MS/MS). The comparison between translated transcriptomic and proteomic data allowed us to identify the expressed venom proteins. Based on similarities in databases, we obtained a number of functional annotated proteins and a group of novel proteins (without any similarities in databases).

By understanding the role of venom in parasitized hosts, we hope to apply these molecules as bioinsecticides in integrated pest control^41,42^.

## Results

### Transcriptome assembly and functional analysis by gene ontology

**Figure 1:**
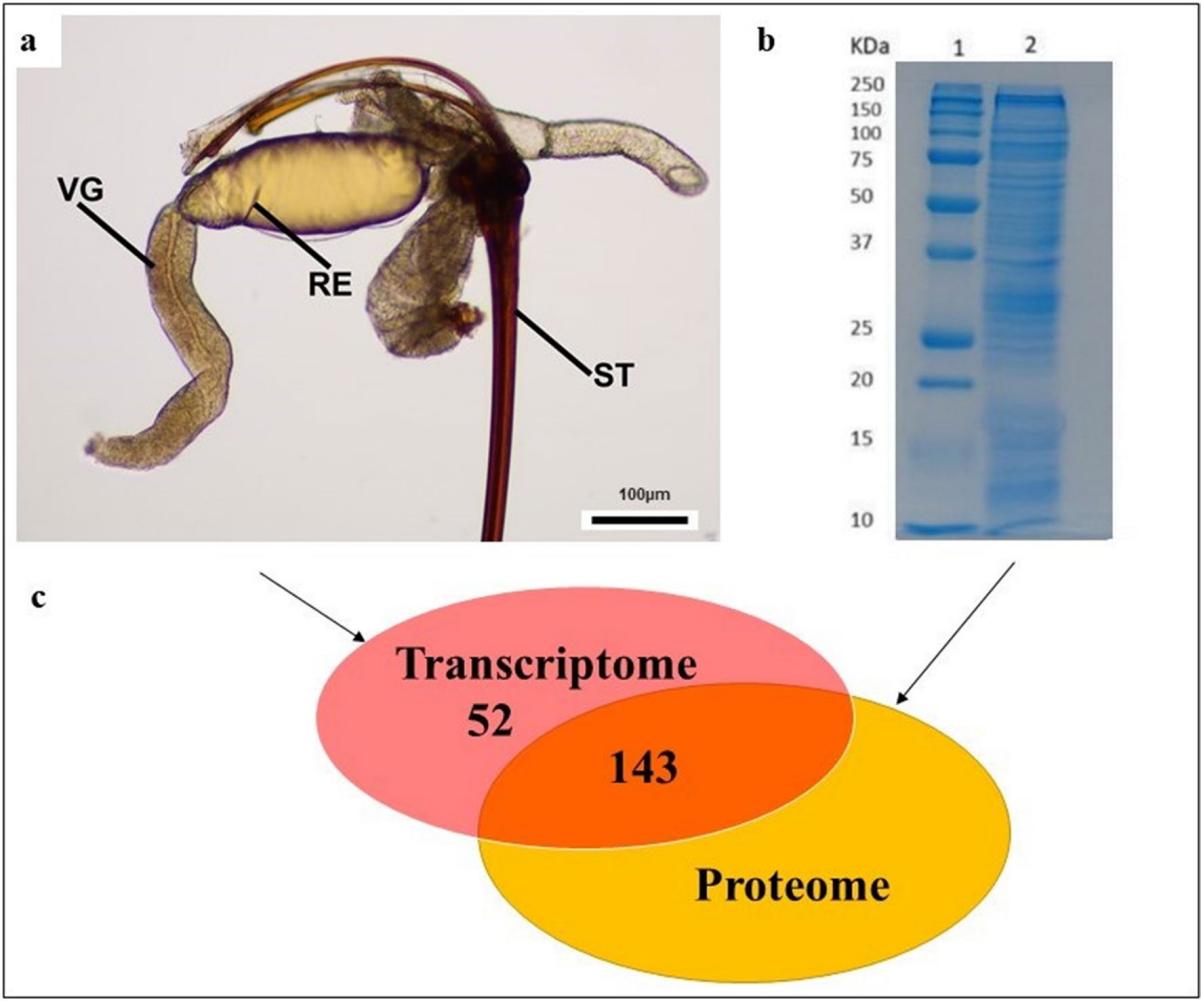
Identification of putative venom proteins in *T. sinensis* (Hymenoptera: Torymidae) combining transcriptomic and proteomic approach. (**a**) Overview of venom gland, reservoir, and sting of the female of *T. sinensis* at the optical microscope. Scale bar 100 µm. *RE* reservoir, *ST* sting, *VG* venom gland. (**b**) SDS-PAGE of crude venom extract from *T. sinensis*. Venom proteins were separated on a 12.5% SDS-PAGE gel (Sigma, St. Louis, MO, USA) and stained with colloidal Coomassie Brilliant Blue G250 (Sigma, St. Louis, MO, USA) (lane 2). Selected protein bands were excised from the gel and processed for LC/MS–MS analysis. Lane 1: molecular marker “All Blue Standards Biorad” (Biorad, Hercules, California, USA) (**c**) venom protein number identified with transcriptomic and combined proteomic and transcriptomic approach: 143 venom proteins were identified through an integrated transcriptomic and proteomic approach and 52 additional transcripts encoding putative venom proteins were identified in venom gland transcriptome through a “venom” keyword research.

Next-generation sequencing (RNAseq) performed with RNA isolated from the venom glands of *Torymus sinensis* (Fig. 1a) allowed us to generate a de novo transcriptome assembly, which contained 22,875 contigs, with a maximum contig length of 19,306 bp. The six reading frames of the 22,875 nucleotide sequences were translated into their corresponding amino acid sequences, resulting in 137,250 predicted proteins (“*T. sinensis* protein database”).

**Figure 2:**
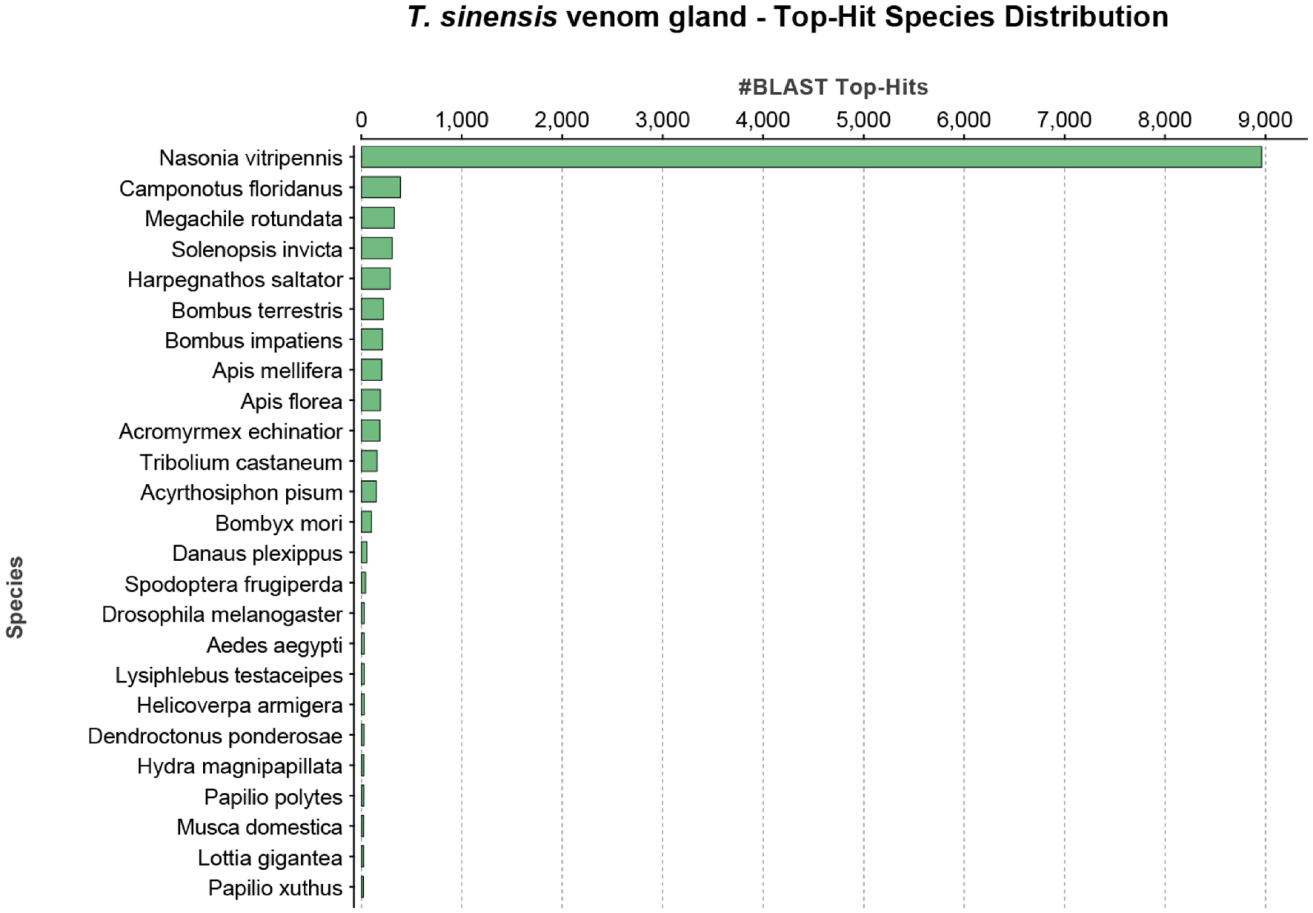
Distribution of top BLAST hit species for the *T. sinensis* transcriptome assembly. Top BLAST hits were obtained from BLASTx analysis against the NCBI non-redundant (nr) protein database. The number of top BLAST hits per species is shown on the x-axis. The highest number of matches were obtained for the ectoparasitoid wasp *Nasonia vitripennis*.

To define similarities with annotated proteins, the contig sequences of the de novo transcriptome of *T. sinensis* venom glands were searched using the BLASTx algorithm^43^ against a non-redundant (nr) NCBI protein database with an E-value cut-off of 10^–5^ identifying 7,466 contigs (33%) matching entries. The species distribution of the top BLAST hit against the nr database for the *T. sinensis* venom gland transcriptome showed that the majority of obtained top hits matched *N. vitripennis* (Fig. 2).

For functional annotation, all sequences were subjected to gene ontology (GO) analysis in Blast2GO, with 12,714 (56%) of the 22,875 contigs sharing significant similarity to proteins with assigned molecular functions in the GO database^43^, whereas 44% of the total transcripts did not match any annotated sequences in the nr database indicating a consistent group of noncoding transcript and species-specific putative proteins or more probably transcript not annotated yet.

**Figure 3:**
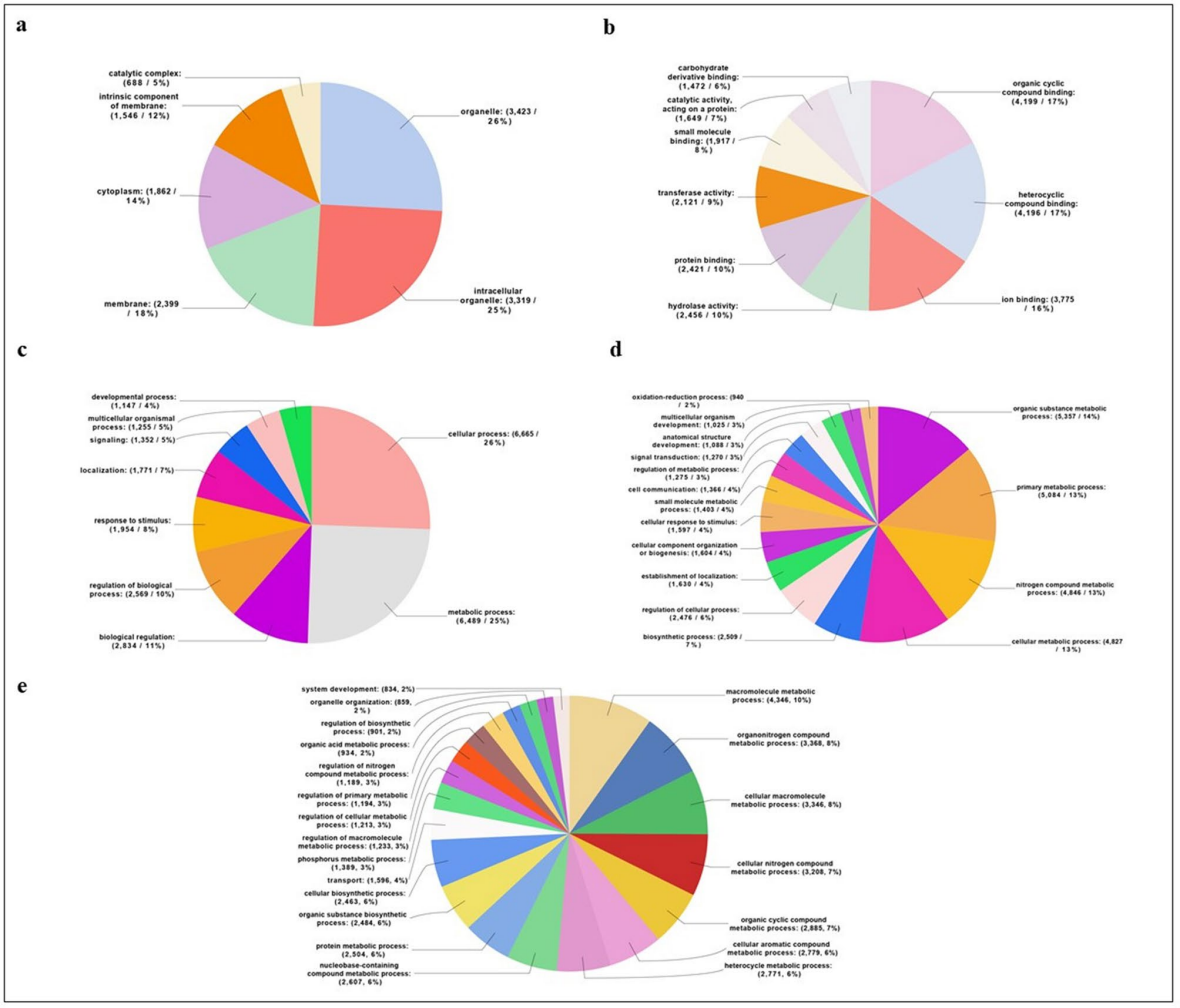
Gene Ontology sequence annotation. Functional classification of all nr-matched transcripts from the *T. sinensis* venom gland. (**a**) Cellular component; (**b**) molecular function; (**c**–**e**) biological process. Data are presented as level 2 GO category for Biological Process (**c**), level 3 GO category for cellular component (**a**), molecular function (**b**) and biological process (**d**) and level 4 GO category for biological process (**e**). Classified gene objects are displayed as total contig number and percentages of the total number of gene objects with GO assignments. Percentages below 2% are not shown.

The 12,714 annotated contigs were classified into three GO categories: biological processes, cellular components and molecular functions. The most prominent GO Cellular Component categories (Level 3) were organelles (51%) (Fig. 3a). The most prominent Molecular Function (Level 3) were different kinds of protein with binding activity (74%), followed by hydrolases (10%) and transferases (9%) (Fig. 3b). The most prominent category of GO Biological Processes (Level 2-3-4) was composed of proteins involved in metabolic and cellular processes (Fig. 3c–e). This result was linked to the very large number of general GO terms, terms that include basic processes for a living organism.

**Figure 4:**
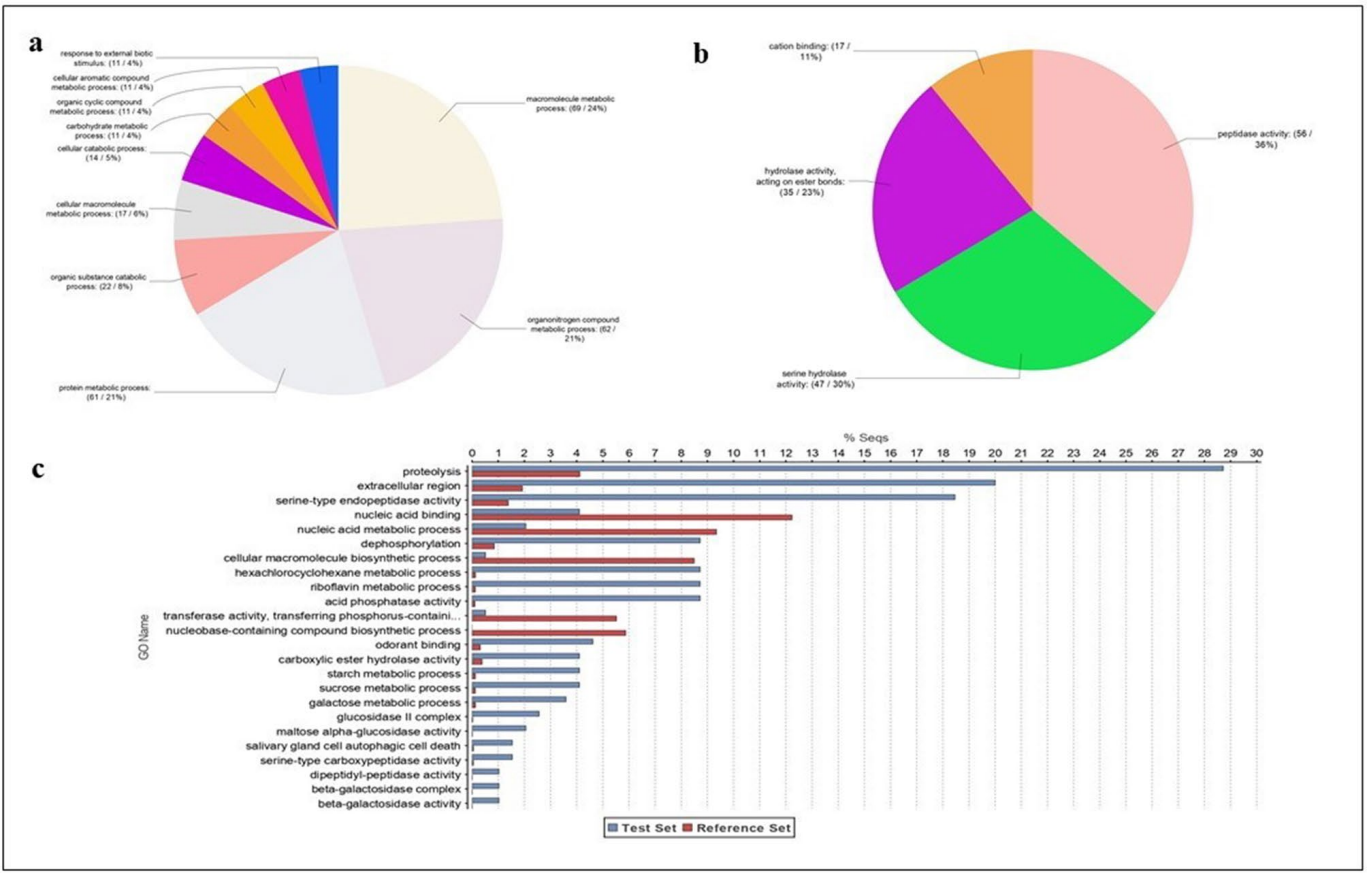
Gene Ontology sequence annotation of *T. sinensis* 195 venom proteins. Gene Ontology (GO) assignments for the *T. sinensis* venom proteins. GO assignments as predicted for their involvement in (**a**) biological processes and (**b**) molecular functions. All data are presented at level 4 GO categorization. Classified gene objects are depicted as absolute numbers and percentages (in brackets) of the total number of gene objects with GO assignments. In (**c**) enriched distribution of Gene Ontology (GO) terms in *T. sinensis* venom proteins were identified. Bar charts show the GO terms that were significantly (false discovery rate (FDR) < 0.05) enriched in the group of venom proteins compared to the complete *T. sinensis* gland transcriptome. The GO terms are sorted in an ascending order according to their FDR value, starting with the most significantly enriched. Only the most specific GO terms are displayed. Differences are shown as the percentage of sequences associated with a specific GO category in the test set (venom protein-encoding contigs) versus the reference set (transcriptome backbone assembly) using Fisher’s exact test in OmicsBox.

A GO analysis was performed on the identified 195 venom proteins (Fig. 4). The most abundant categories of Biological Processes (Level 4) were macromolecules, proteins and organonitrogen metabolic processes (Fig. 4a). Four macro-categories were identified through Molecular Function (Level 4) analysis: peptidases, serine proteases, hydrolases and cation binding activity proteins (Fig. 4b). A further "Enrichment Analysis" highlighted that proteins with proteolytic and serine-type endopeptidase activity are the most abundant in *T. sinensis* venom*,* comparing venom protein components and total *T. sinensis* transcripts (Fig. 4c).

**Figure 5:**
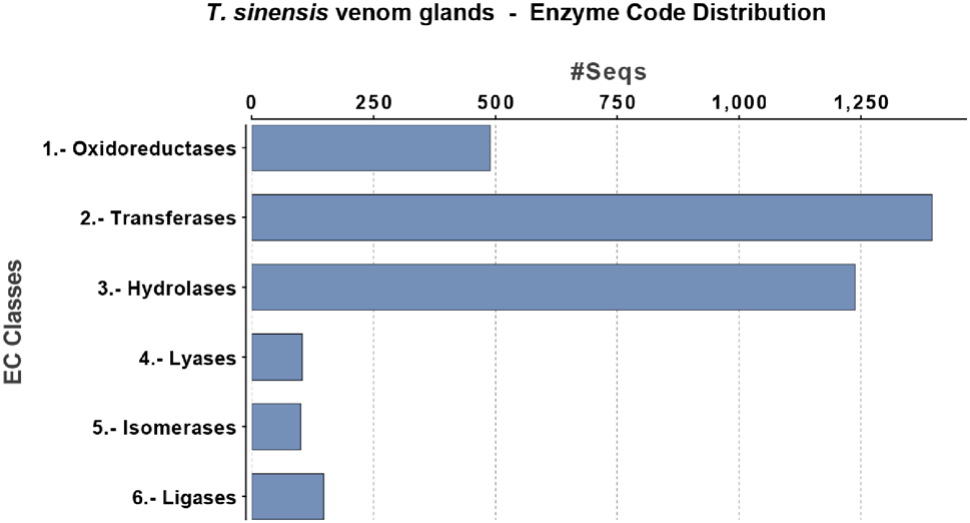
Enzyme Code (EC) Classes of the *T. sinensis* contigs encoding enzymes. The most abundant families of enzymes found in the transcriptome of *T. sinensis* venom glands are shown.

As previously reported, contigs not matching any known sequences in the nr database are relevant. The relatively high number of contigs without significant BLAST matches is quite common in the case of de novo transcriptome assemblies obtained from RNA-Seq data, and could be due to fragmented assembly, resulting in larger numbers of contigs corresponding to untranslated (UTR) regions of cDNAs, noncoding transcripts or species-specific un-annotated orphan genes. In *T. sinensis* venom transcriptome, the enzyme code distribution shows that the most abundant families of enzymes were transferases and hydrolases (Fig. 5).

A quantitative RNA-Seq analysis of the transcripts encoding for putative venom proteins based on the Reads Per Kilobase of transcript method, per Million mapped reads (RPKM) showed large differences in expression levels; the most expressed transcripts in the venom glands are reported in Table 1.

**Table 1:** Most expressed *T. sinensis* venom proteins.

Contig	Protein name	RPKM
445	Alpha-amylase-like	9,77118
4	Serine protease 33 isoform X2	9,71077
270	Trypsin-like	9,66998
1650	Peptidyl-prolyl cis–trans isomerase 5	9,54057
157	Venom protein U precursor	9,49706
1105	Serine protease inhibitor 3/4 isoform X16	9,17066
293	Cathepsin L	8,64858
2095	Carboxylesterase clade B, member 2 precursor	8,59989
289	Low-density lipoprotein receptor-related protein 2-like	8,58940
428	Lysosomal aspartic protease-like	8,45276
940	Heat shock protein 70	8,39405
1194	Ferritin precursor	8,33117
1001	Calreticulin precursor	8,31852
1747	Endoplasmin	8,09155
1971	Chymotrypsin-1-like	7,94740
392	Lipase 3-like	7,90375
181	Protein disulfide-isomerase A3	7,84263
495	Pancreatic triacylglycerol lipase	7,75945
4156	Ribonuclease Oy-like	7,67279
3120	Venom allergen 5-like	7,52232
874	Adipocyte plasma membrane-associated protein-like	7,17361
2736	Chitotriosidase-1 isoform X1	7,14344
128	Pancreatic triacylglycerol lipase	7,03636
1330	Alpha-glucosidase-like	6,95432
1835	Serine protease homolog 21 precursor	6,76623
1890	Ovomucoid-like	6,61221
3708	Trypsin beta-like	6,55134
2538	Apolipoprotein D-like	6,40265
575	Mesencephalic astrocyte-derived neurotrophic factor homolog	6,31104
2298	Dipeptidase 1 isoform X1	6,29161
69	Trypsin-3-like	6,28669
18	Serine protease inhibitor 28Dc isoform X2	6,21690
1034	Vitellogenin	6,21393
2025	Serpin 5 precursor	6,17429
5412	Calnexin isoform X2	6,02715
3815	Inosine-uridine preferring nucleoside hydrolase-like precursor	6,02358

Proteins identified by SDS-PAGE and LC–MS/MS of venom extract, ordered by descending RPKM values. Expression levels are reported as log2-transformed RPKM values.

### Gene expression levels in *Torymus sinensis* venom gland

**Figure 6:**
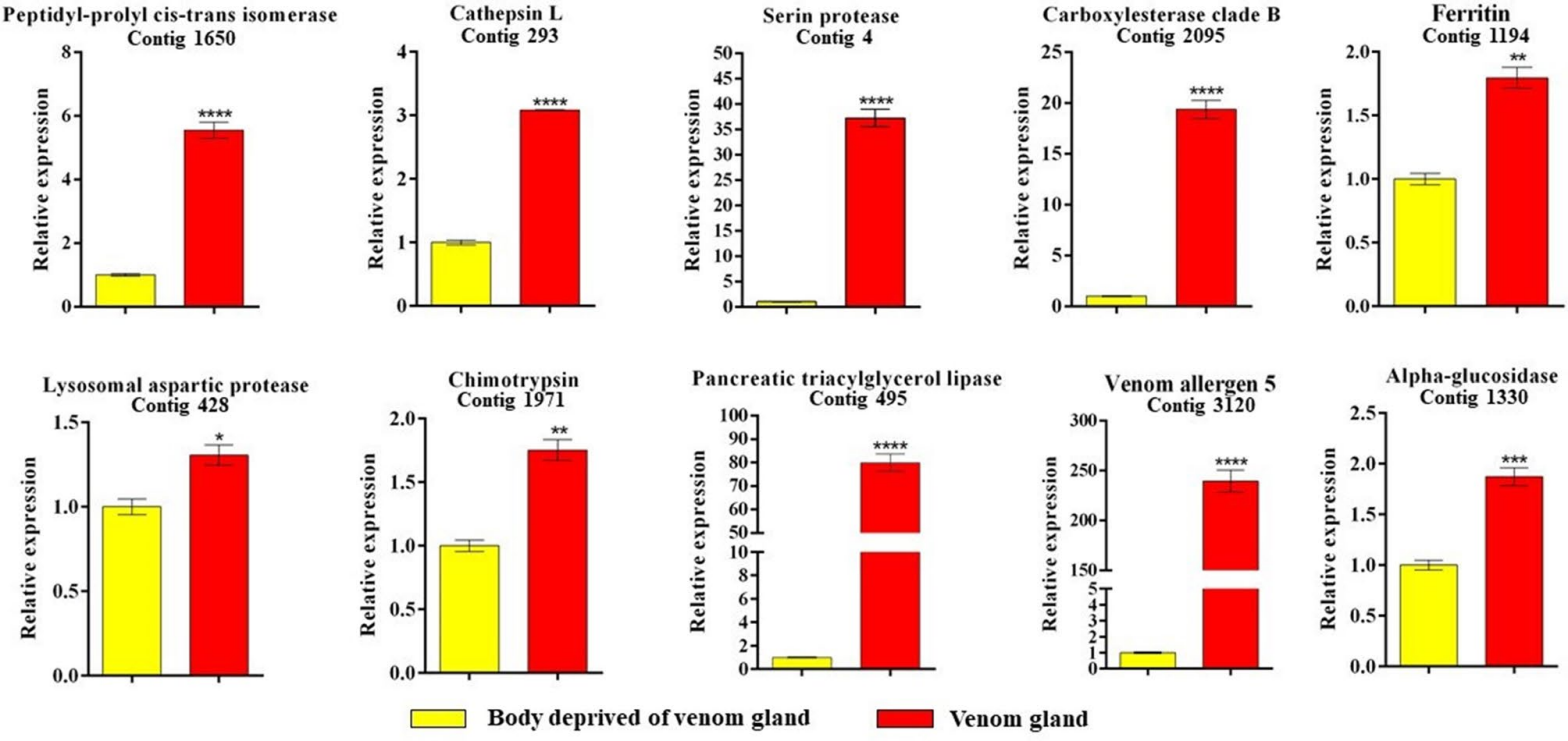
Relative expression level of ten selected genes of *T. sinensis* venom gland and *T. sinensis* female body deprived of venom gland. Gene expression levels were quantified by quantitative real time PCR (qPCR). Data represent the mean of three independent replicates ± SEM. Samples were compared by the Unpaired t-test and statistically significant differences between samples are indicated with asterisk (*p = 0.015, **p = 0.011, ***p = 0.0009, ****p < 0.0001). Reference genes: GAPDH and beta-tubulin. Calibrator sample: female body deprived of venom gland.

To confirm the specific expression of genes in *T. sinensis* venom gland, a quantitative real time PCR (qPCR) experiment was performed on RNA extracted from venom glands and females deprived of the venom gland, focusing on a sample set of 10 genes selected among transcripts reported in Table 1. Female body without venom glands was used as calibrator. A significantly higher transcription level in venom glands was observed for all the selected genes (Fig. 6).

### SDS-PAGE and mass spectrometry

The one-dimensional SDS-PAGE electrophoretic profile showed that the *T. sinensis* venom consists of a complex protein mixture whose molecular weights range from 10 to 250 KDa (Fig. 1b). The sample lane containing the venom proteins was cut into thin slices, and the protein bands were excised, destained and subjected to in situ proteolytic digestion with trypsin. Protein identification was performed by direct nanoLC–MS/MS analysis of the corresponding peptide mixtures. Mass spectral data were processed with the Andromeda^44^ search engine and then compared with the “*T. sinensis* protein database”, leading to the identification of the proteins expressed in venom. Mass spectrometry analysis provided a list of 15,381 peptide sequences.

### Proteomic and transcriptomic data analysis

The comparison between proteomic and transcriptomic data sets led to the identification of 1322 contig. The surprisingly large number of proteins identified by mass spectrometry is common^20,27,28,45^. Further analyses were performed with the software Signal P 5.0 (http://www.cbs.dtu.dk/services/SignalP/) in order to select only proteins bearing the signal peptide and therefore are likely to be secreted. This in silico prediction allowed us to select a total of 195 secretory proteins.

To further confirm the venomous nature of these 195 proteins, their sequences were aligned and compared to venom proteins from *Nasonia vitripennis*, described in de Graaf et al*.*^30^, obtaining 74 matches reported in Supplementary Tables S1, S2 and S3.

Among the 195 *T. sinensis* venom proteins, 121 proteins with signal peptides did not match any *N. vitripennis* venom protein. Although two contigs (12 and 2618) are not annotated, as they have no similarity with other known proteins, 69 proteins were found to show significant similarity with venom protein components of other parasitoid wasps or venomous animals or were uncharacterized proteins.

The 143 putative venom protein identified with the proteomic approach are listed in Supplementary Table S4 (Fig. 1c). The remaining 50 proteins without any similarity to known components of insect or other animal venom were considered either ‘venom trace elements’ with a limited function in the venom duct or in the reservoir, or contaminants of the venom gland released during the dissection of the gland tissue^30,46^. In addition to the proteomic approach, a key-word approach was used to identify a further group of putative venom protein in the venom gland transcriptome: all putative proteins annotated with the word “venom” were selected. 52 contigs annotated with “venom” keyword were identified (Supplementary Tables S5, S6; Fig. 1c). In addition, 2 contigs annotated with the “toxin” keyword were also identified (Supplementary Tables S5, S6). 18 of these contigs showed the signal peptide while this signature was missing in 34 contigs (Supplementary Table S5). Among the contigs missing the signal peptide, 18 had an incomplete sequence at 5 ' end, 14 had a complete sequence and 2 had an open reading frame (ORF) too small to give information about signal peptide (Supplementary Table S6).

To further confirm that the 52 putative proteins annotated using “venom” as a keyword belonged to the mixture of *T. sinensis* venom protein components, the nucleotide sequences of these proteins were translated using Expasy-translate tool software (https://web.expasy.org/translate/), and the corresponding protein sequences were compared to those of *N. vitripennis*, reported in de Graaf et al*.*^30^, obtaining 46 matches reported in Supplementary Tables S7, S8, S9. 9 *T. sinensis* contigs did not match any of the *N. vitripennis* venom proteins but were similar to other venom protein components, including carboxylesterases, Kazal-type serine protease inhibitors, acid phosphatases and carboxypeptidases (Supplementary Table S10).

All *T. sinensis* venom proteins, identified by the proteomic approach or exclusively by using the transcriptomic approach, can be classified in different groups, according to their functional annotation (Table 2).

**Table 2:** *T. sinensis* venom proteins, identified by the transcriptomic or proteomic approach, categorized according to their role.

Protein category	Contig number	Approach
**Hydrolases**		
Serine proteases	1, 4, 25, 69, 70, 87, 125, 235, 270, 368, 378, 706, 753, 775, 789, 991, 1058, 1059, 1089, 1143, 1233, 1310, 1617, 1636, 1794, 1835, 1855, 1907, 1971, 2101, 2283, 2399, 3012, 3708, 5058, 6126, 6382, 11,004, 11,592	Proteomic
	7259, 8948	Transcriptomic
Metalloproteases	8199	Proteomic
Dipeptidases	2298, 6303, 11,199	Proteomic
	7070	Transcriptomic
Aminopeptidases	5399, 7646, 18,029	Proteomic
Lysosomal proteases	293	Proteomic
Carboxypeptidases	1049, 12,519	Proteomic
	7893, 15,076	Transcriptomic
Venom acid phosphatases	3591, 3878	Proteomic
	3070, 7321, 7343, 7836, 8187, 8691, 8857, 10,928, 13,041, 13,453, 14,872, 14,873, 16,876, 16,269, 18,015, 18,733, 18,844, 20,332	Transcriptomic
Carboxylesterases	106, 1651, 1652, 2095, 2872, 3239, 9844	Proteomic
	4090, 7684, 11,671, 12,240, 15,429, 19,895, 21,975	Transcriptomic
Lipases	128, 392, 495	Proteomic
Glucosidases	1330, 2201, 5291, 8029, 10,216, 10,716	Proteomic
Galactosidases	7966, 4813	Proteomic
Amylases	445, 5249	Proteomic
Trehalase	4724	Proteomic
Chitinases	2736	Proteomic
Nucleases	2219, 3815, 4156, 4479, 6082,	Proteomic
	1587, 1953	Transcriptomic
**Protease inhibitors**		
Serine protease inhibitors	18, 1105, 2025	Proteomic
Kazal type serine protease inhibition-like venom proteins	1890	Proteomic
	1535, 11,921	Transcriptomic
Cysteine-rich proteins	5346, 21,875	Transcriptomic
**Immune related proteins**		
Calreticulin	1001	Proteomic
Calnexin	5412	Proteomic
Nucleobindin	4917	Proteomic
C1q-like venom protein	9506	Transcriptomic
**Recognition/binding proteins**		
Odorant binding proteins	281, 590, 1303, 1372, 4531, 4663, 7398, 8693, 9106, 10,799, 14,598	Proteomic
Chemosensory proteins	5573	Proteomic
Low-density lipoprotein receptors	289, 2622	Proteomic
	1256	Transcriptomic
Apolipophorines	784, 2538	Proteomic
Insulin-like growth factor-binding protein	2057	Proteomic
**Glutathione metabolism**		
γ-Glutamyl cyclotransferase	5910	Transcriptomic
**Oxidase**		
Laccase	17,081	Transcriptomic
**Dehydrogenases**		
Alcohol dehydrogenase	2176	Proteomic
**Isomerases**		
Peptidyl-prolyl cis/trans isomerase	1650	Proteomic
FK506-binding protein	3257	Proteomic
Protein disulfide-isomerase A3	181	Proteomic
**Heat shock proteins**		
Endoplasmin	1747	Proteomic
Heat shock 70 kDa	940	Proteomic
**Other proteins**		
Allergens 3 and 5	3120, 8578	Proteomic
	1427, 7940, 12,687, 12,783	Transcriptomic
Major royal jelly protein	11,185	Proteomic
Adipocyte plasma membrane-associated protein	874	Proteomic
Ferritin	1194, 1432	Proteomic
Lachesin	3847	Proteomic
Mesencephalic astrocyte-derived neurotrophic factor	575	Proteomic
Vitellogenin	1034	Proteomic
Agatoxin	12,568	Transcriptomic
**Unknown and hypothetical proteins**		
Venom protein D	3227	Transcriptomic
Venom protein F	1029	Transcriptomic
Venom protein L	2092	Transcriptomic
Venom protein N	10,580	Transcriptomic
Venom protein O	19,579	Transcriptomic
Venom protein Q	472	Transcriptomic
Venom protein R	2910	Proteomic
Venom protein T	2487	Transcriptomic
Venom protein U	157, 1301	Proteomic
Venom protein V	4647	Transcriptomic
Uncharacterized proteins	158, 266, 1114, 1951, 3464, 3779, 5267, 5502, 6782, 7280, 7912, 7916, 8096, 8212, 9897, 10,521	Proteomic
Hypothetical proteins	529, 990, 1109	Proteomic

The table contains the contig number and the approach by which each protein was identified.

Among the identified proteins, hydrolases constituted the most abundant family followed by protease inhibitors, recognition and binding proteins, isomerases and dehydrogenases. These findings are in agreement with data reported for the venom of other parasitoids^18,28,29,47^. Among the hydrolases in the *T. sinensis* venom glands, we identified proteases and, in particular, serine proteases, metalloproteases, different kinds of peptidases, proteins of carbohydrate metabolism, lipases, phosphatases and nucleases.

## Discussion

In order to identify the venom proteins of the ectoparasitoid *Torymus sinensis*, a combined proteomic and transcriptomic approach was used (hereafter defined as the proteomic approach). The transcriptomic analysis provided general information about putative proteins of the venom gland, focusing on their molecular functions, biological processes, and cellular compartments. This first level of analysis allowed us to select putative candidate proteins, annotated as “venom/toxin”. The proteomic analysis was performed on the venom extract using mass spectrometry, and the expressed proteins were identified when their sequences matched with the translated transcripts (contigs) of the venom gland transcriptome. Proteins containing a putative signal peptide for extracellular localization and predicted cleavage sites were reported as venom-expressed components. All putative (predicted from the transcriptome) or expressed (confirmed by the proteomic approach) proteins identified as venom protein components were further compared to *Nasonia vitripennis* venom proteins described by de Graaf et al*.*^30^, as both ectoparasitoids belong to the Chalcidoidea superfamily. The comparison with *N. vitripennis* venom proteins further confirmed the venomous nature of *T. sinensis* predicted and expressed proteins. We also identified putative or expressed *T. sinensis* venom proteins showing similarity to venom protein components of other parasitoids or animals. Lastly, a group of *T. sinensis* venom proteins was identified with sequence similarities to unknown, hypothetical or uncharacterized proteins. All the identified proteins were grouped according to their functions, and their possible role in the complex parasitic syndrome after envenomation was discussed.

### Hydrolases

Hydrolases have been found in several endo- and ectoparasitoid venoms^48^. The hydrolases identified in *T. sinensis* venom can be grouped in different classes: proteases (serine proteases, metalloproteases, dipeptidases, amino- and carboxypeptidases), phosphatases, esterases, lipases, glucosidases, galactosidases, amylases, trehalases, nucleases. Some of these proteins belong to carbohydrate metabolism, including glucosidases, galactosidases, amylases and trehalases.

Proteases have been reported as abundant components in numerous parasitoid venoms^18,20,27–29,49^.

Among proteases, serine proteases are the most abundant family, including trypsins, chymotrypsins and serine protease homologues. Serine proteases and serine protease homologues were found in different parasitoid species, such as the endoparasitoids *Aphidius ervi*^49^, *Microplitis mediator*^47^, *Pimpla hypochondriaca*^24^, *Pteromalus puparum*^29^, *Toxoneuron nigriceps*^18^ and *Cotesia rubecula*^50^. Serine proteases can play a crucial role in regulating the immune system by inhibiting melanization in the host hemolymph blocking the phenoloxidase cascade^51,52^. Generally, serine protease homologues can act as co-enzymes for prophenoloxidase-activating proteinases and are important for the activation of prophenoloxidase and melanization^53^. In *C. rubecula*, a particular protease named Vn50 was structurally similar to other serine protease homologues but showed a different function. Vn50 inhibits melanization presumably by competing with host serine protease homologs for binding the prophenoloxidase that remains un-cleaved in the hemolymph^50,51^. Besides the action of serine protease homologues, serine protease may also play a role in down-regulating prophenoloxidase^20,54^. A different model for the involvement of venom serine protease in the melanization response was proposed by Choo et al.^55^ In the venom of *Apis mellifera* and *Bombus spp.*, serine proteases seemed to be involved in the hyperactivation of prophenoloxidases, resulting in an excessive melanization response leading the target insects (*Bombyx mori*, *Spodoptera exigua* and *Pieris rapae*) to death. A similar poisoning effect may be induced by injecting ectoparasitoid venoms into the host. In addition to its involvement in host immunity disruption, a serine protease in *N. vitripennis* venom showed a putative cytotoxic function in assays with a *Spodoptera frugiperda* cell line^56^, whereas a trypsin-like enzyme found in salivary secretions of the ectoparasitoid *Euplectrus separatae* larva seemed to be able to digest host tissues^57^, prompting the hypothesis that serine proteases are extensively involved in the parasitic syndrome. In *T. sinensis* venom, many serine proteases were identified: trypsins (peptides identified by the proteomic approach matching to 25 contigs), chymotrypsins (peptides identified by the proteomic approach matching to 6 contigs), general serine proteases (peptides identified by the proteomic approach matching to 7 contigs, and 2 contigs identified by the transcriptomic approach) and a serine protease homologue (identified by the proteomic approach). Seven trypsins, and two general serine proteases identified in *T. sinensis* venom did not show the classic serine protease catalytic site, composed of serine, histidine and aspartic acid.

In the predicted serine proteases identified from the *T. sinensis* data, the relative positions of the amino acids of catalytic triad are mostly conserved: histidine at position 70, aspartic acid at positions 125 and 130, and serine at positions 240 and 245. A few anomalies have to be mentioned: in three contigs, the catalytic triad showed slight differences; in a trypsin, the serine residue is substituted by arginine, and in the serine protease homologue, the histidine residue is substituted by glycine. For some contig-derived amino acid sequences, the absence of the catalytic triad can be explained by the incompleteness of the contig sequence.

The high number of serine proteases identified as part of the complex *T. sinensis* venom and the absence of clear one-to-one orthology to other insect proteases (Supplementary Fig. S2), including *N. vitripennis* venom proteins, indicates species-specific gene duplication events in *T. sinensis*. Such large-scale gene duplication events of the serine protease gene family is a frequent phenomenon^29,58^ and we suspect that a greatly expanded set of proteases is associated with complex venom function.

*Metalloproteases* are involved in several biological and disease-related processes, such as intracellular signalling, matrix degradation, inflammation, and coagulation disorders^59,60^. In insect, metalloproteases are related to the immune response; indeed zinc-dependent proteases were highly expressed in *Manduca sexta* larvae infected with bacteria, and one of these proteases was quite similar to human neutral endopeptidase NEP 24.11, which is involved in the immune response^61^. In addition, metalloproteases were found in the venom of *P. hypochondriaca*^24^ and *M. mediator* endoparasitoids^62^, and *Eulophus pennicornis* ectoparasitoid^63^*.* In the latter cases, metalloproteases were shown to be responsible for the alteration of host development, reducing and even blocking host larval growth and metamorphosis, and promoting parasitoid development^47,63^. Some of *M. mediator* metalloproteases belong to the M12B subfamily, a member of which was also identified in the venom of endoparasitoid *T. nigriceps*^18^. According to the MEROPS database (http://merops.sanger.ac.uk), the *T. sinensis* venom metalloprotease, identified by the proteomic approach, belongs to the M12B subfamily, whose components are able to regulate processes related to neoplastic progression in mammals, such as immune response evasion, matrix degradation, metastasis and inflammation processes^64^.

*Dipeptidases* including Dipeptidyl peptidase IV, dipeptidase 1 and the Angiotensin-converting enzyme, are exopeptidases that catalyze hydrolysis of dipeptides^65^.

Dipeptidyl peptidase IV is a very common enzyme in snake venom^66^ and in the venom of some Hymenoptera, such as *A. mellifera*^67^, *Vespa basalis*^68^ and *Polistes dominula*^69^. Although its function is not completely known, it seems to be involved in the processing of precursors of venom protein components^70^. This enzyme is a serine protease that cleaves dipeptides from the N-terminus of peptides with proline or alanine in the penultimate position^71^, and its function could be related to the maturation of toxic peptides, as proposed for mastoparan B, the major toxin in the venom of *V. basalis*^68^. Mastoparan B is indeed synthesized as pro-peptide and then activated through an enzymatic cleavage by a dipeptidyl peptidase IV able to generate a consecutive release of dipeptides^68^. Dipeptidyl peptidase IV cleavage activity was also related to other functions, such as the regulation of inflammatory and immunological responses, signal transduction and apoptosis by degrading physiological substances^67^. The angiotensin-converting enzyme is a peptidyl dipeptidase that removes dipeptides from the C-terminus of short oligopeptides. In mammals, angiotensin I is converted into angiotensin II and bradykinin is activated, thus regulating blood pressure and electrolyte homeostasis. This enzyme was identified in the venom of the endoparasitic wasp *P. hypochondriaca*^5^, in *Thalassophryne nattereri* and *Scorpaena plumieri*, venomous fishes typical of the north-eastern coast of Brazil^72^. Although its specific function remains unknown, it is probably involved in processing peptide precursors.

In *T. sinensis* venom, two contigs encoding for dipeptidyl peptidase IV proteins were identified, one of them using the proteomic approach and the other one by the transcriptomic approach. A dipeptidase 1 and one Angiotensin-converting enzyme were identified using the proteomic approach.

*Aminopeptidases* remove one or more specific N-terminal residues from target proteins or peptides, and are common in venom of snakes^73,74^, in the venom of the predatory ant *Pachycondyla striata*^75^, in the venom of the velvet spider *Stegodyphus mimosarum*^76^ and in the venom of the genus *Thoracobombus* (Hymenoptera)^77^, even if its function has not been understood yet. Mammalian aminopeptidases, which cleave brain angiotensin II to angiotensin III, are implicated in the control of arterial blood pressure^78^. This suggests that aminopeptidases might be involved, in synergy with angiotensin-converting enzymes, in angiogenic mechanisms, such as in regulating blood vessel formation and blood pressure^79^. However, the most probable functions in venoms are 1) to help degrade the host tissues extracellular matrix in order to increase its permeability to venom protein components^74^ and 2) to contribute in transforming host tissues into nutrients for parasitoid progeny. In *T. sinensis* venom, three aminopeptidases were identified using the proteomic approach.

*Lysosomal proteases* belong to the aspartic, cysteine, or serine endoprotease family; despite the adjective “lysosomal,” they are usually detected within all vesicles of the endocytic pathway. They are also known as cathepsins^80^. Lysosomal aspartic proteases are enzymes whose catalytic sites consist of two aspartate residues^81^. A similar protein, a cathepsin D, was found in the venom of the endoparasitoids *Leptopilina heterotoma*^82^ and *Chouioia cunea*^83^. A putative lysosomal aspartic protease was also upregulated in the pupal transcriptome of *Sarcophaga crassipalpis* 25 h after envenomation by *N. vitripennis*, supporting the hypothesis that this protease plays a key role in the success of parasitism^84^. Lysosomal proteases, such as cathepsins, are activated in apoptotic and necrotic cells and during autophagy phenomena^81^. The regulation of autophagy, which is associated with starvation, nutrient recycling and cell cycle arrest, can be a strategy used by parasitoids to manipulate host development and metabolism for its progeny^84^. Although the specific role of lysosomal proteases in Hymenoptera parasitoids is still unclear, they may be involved in the production of venom protein components or in blocking host immunity, contributing to their offspring development^82^. In *T. sinensis* venom, one lysosomal aspartic protease was identified using the proteomic approach. Cathepsin L is a lysosomal cysteine protease with a catalytic dyad consisting of cysteine and histidine^85^. Lysosomal cysteine proteases are involved in extra- and intra-cellular protein degradation, antigen presentation and cellular development^86^, and in various orders of insects are considered important digestive enzymes^87^. In parasitoid venom, cathepsin L was first found in *T. nigriceps* venom^18^. A large quantity of this protein was found in fat body/hemocytes complex of a *Spodoptera littoralis* larva parasitized by the ectoparasitoid *Bracon nigricans*^88^; in this latter case it was hypothesized that cathepsin L induced the formation of these complexes, because of the rapid degradation of fat body required to mobilize the stored nutrients in favor of the parasitoid offspring^88^. In *T. sinensis* venom, one cathepsin L was identified using the proteomic approach. Moreover, a protein annotated as “uncharacterized”, identified by the proteomic approach, might also be included in the Lysosomal protease category as it contains a cysteine protease domain and a region similar to cathepsin L.

*Carboxypeptidases* cleave peptide bonds at the C-terminal of a protein^89^. Putative venom serine carboxypeptidases were found in the venom of the ants *Odontomachus monticola*^90^ and *Tetramorium bicarinatum*^91^, the snake *Crotalus durissus terrificus*^92^, the Hymenoptera of the Apidae family^77^ and in the venom gland of the ectoparasitoid *Anisopteromalus calandrae*^31^. A serine carboxypeptidase was also found in the venom of the endoparasitoids *Psyttalia concolor*^93^ and *T. nigriceps*^18^. Although the specific role of carboxypeptidases in parasitoid venom is not clear yet, this enzyme could be involved in the degradation of the host tissues, most likely as the aminopeptidase enzymes. In *T. sinensis* venom, four carboxypeptidases were identified: three using the proteomic approach, and one using the transcriptomic one.

*Venom acid phosphatases* have been identified in the venom of some hymenopteran species, such as *A. mellifera* and *Apis cerana*^94,95^, the endoparasitoids *P. hypochondriaca*^96^, *Pimpla turionellae*^45^, *P. puparum*^97^ and the ectoparasitoids *A. calandrae*^31^ and *Bracon hebetor*^98^. Venom acid phosphatases were also identified in the venom gland of the endoparasitoid *M. pulchricornis*^99^. They are characterized by a conserved catalytic core containing a histidine residue which is phosphorylated during catalysis. In venom, they have a neurotoxic, myotoxic, anticoagulant and inflammatory effect^100^. In *T. sinensis,* two acid phosphatases were identified by the proteomic approach, and eighteen acid phosphatases were identified by the transcriptomic approach.

*Carboxylesterases* are hydrolases containing a catalytic apparatus consisting of three residues, serine, glutamate or aspartate and histidine, and the mechanism involves a nucleophilic attack on a carbonyl carbon atom^101^. Although their function in venoms has not been identified yet, they could be allergen proteins; indeed, carboxylesterase-6 is one of the main allergens in honeybee venom^102^. In *N. vitripennis* venom, two types of esterases have been found: an arylsulfatases B isoform X1 and a carboxylesterase clade B member 2 precursor^30^. In *T. sinensis* venom, seven carboxylesterases were identified by the proteomic approach and seven by the transcriptomic one.

*Lipases,* which act on carboxylic esters, have the same catalytic triad as esterases. In general, lipases have essential roles in the digestion, transport, and processing of dietary lipids in most living organisms^103^. It has been demonstrated that *N. vitripennis* venom induces alterations in the host lipid metabolism, although the specific role of lipases has not been clarified yet^104^. Recently, lipase activity has been found in the venom of the endoparasitoid wasps *P. hypochondriaca*^96^, *Psyttalia lounsburyi* and *P. concolor*^93^, *Microctonus aethiopoides*^105^, *Ooencyrtus telenomicida*^106^ and the ectoparasitoid *B. nigricans*^32^. An interesting example is reported in the endoparasitoid *Cotesia kariyai*: several days after parasitization, the total amount of lipid from the fat body of the parasitized hosts decreased as the lipase activity of parasitoid larvae increased. Although in this case lipase was not annotated as a component of the endoparasitoid venom, this observation could support the hypothesis that, in general, lipases can digest host lipids and provide nutrients to the parasitoid larvae^54,107^. Lysis of fat body cells, with the increase in the hemolymphatic lipid content, was also observed in the lepidoptera *Pseudaletia separata* after it was parasitized by ectoparasitoid wasps of the genus *Euplectrus*^108^. Lipases can then be considered involved, at least partially, in increasing the suitability of the host environment in favor of parasitoid progeny. In *T. sinensis* venom three lipases were identified by the proteomic approach.

*Glucosidases* hydrolyze glycosidic bonds from glycosides and oligosaccharides and remove non-reducing terminal glucosyl residues releasing glucose as product^109^. Among parasitoids, β-glucosidases were detected in the venom of the endoparasitoids *P. hypochondriaca*^96^, *Microplitis demolitor*^110^, *P. lounsburyi* and *P. concolor*^93^. The release of glucose, deriving from host hemolymph carbohydrates, may increase the amount of energetic nutrients available for the developing parasitoid larvae, suggesting that glucosidases are involved in modifying host metabolic pathways in favor of parasitoid development. In *T. sinensis* venom six glucosidases were identified by the proteomic approach.

*Galactosidases* catalyze the hydrolysis of galactoside molecules by breaking glycosidic bonds^111^. Among parasitoids, galactosidases were identified in the venom of the endoparasitoid *P. hypochondriaca*^96^. Like glucosidases, galactosidases could also release carbohydrates into the host hemolymph for supply of parasitoid larvae. In *T. sinensis* venom, two galactosidases were identified by the proteomic approach.

*Amylases* are enzymes catalyzing the hydrolysis of alpha-1,4 glycosidic bonds of starch and glycogen into sugars, such as maltose, maltotriose and residual branched maltodextrins^112^. Most amylases have been identified in insect salivary glands or digestive tracts; few examples have been reported in the venom of parasitoid wasps, such as *Nasonia* species and *P. puparum*^113,114^. As carbohydrates are essential for metabolism, it is proposed that amylases expressed in the venom of *P. puparum* and secreted in the host (*P. rapae*) hemolymph are involved in the degradation of host polysaccharides for energy intake and parasitoid larvae development^114^. In *T. sinensis* venom, two amylases were identified by the proteomic approach.

*Trehalases,* enzymes which catalyze the conversion of trehalose to glucose, have been found in the venom of wasps such as *Cerceris rybyensis*^115^, the endoparasitoids *Ampulex compressa*^116^, *P. hypochondriaca*^117^ and the ectoparasitoid *N. vitripennis*^118^. Trehalases may convert the high concentration of trehalose in the host hemolymph to glucose in order to provide a source of energy for the parasitoid larva development^118,119^. In *T. sinensis* venom, one trehalase was identified by the proteomic approach.

Collectively glucosidases, galactosidases, amylases and trehalases may play a key role in metabolic pathways, providing nutrients to the parasitoid offspring.

*Chitinases* are able to disrupt and digest chitin, one of the components of the exoskeleton of arthropods^120^. This enzyme is vital for animals during ecdysis and metamorphosis, as well as for animals that feed on organisms whose structures are composed of chitin. Although this enzyme has been detected in the venom of spiders^121^ and scorpions^122^, it is also important in the physiology of different endoparasitoids, like *T. nigriceps,* where teratocytes release chitinases to support the emergence of larvae by degrading the host cuticle^123^. A similar process may be performed by chitinase in the venom of the endoparasitoid wasp *Chelonus inanitus*, helping the parasitoid larva to hydrolyze the host embryonic cuticle to reach host embryo^124^. In *T. sinensis* venom, one chitotriosidase was identified by the proteomic approach. Moreover, a protein annotated as “uncharacterized protein”, found by the proteomic approach was included in this group as it contains chitin-binding domains in its structure.

*Nucleases* are capable of cleaving the phosphodiester bonds among nucleotides. Nucleases are very common in snake^125^ and Cnidaria venoms^126^. It was predicted that these enzymes play a central role in strategies of prey immobilization, as free adenosine molecules may induce the inhibition of neurotransmitter release. Moreover, these enzymes may induce renal failure and cardiac arrest and increase vascular permeability, thereby helping the spread of toxins in host tissue^125^. Endonuclease-like venom proteins are characterized by a DNA/RNA non-specific endonuclease conserved domain that gives the protein the ability to cut double-stranded and single-stranded nucleic acids^127^. They were found in the venom of the ectoparasitoid *A. calandrae*^31^ and the endoparasitoid *Cotesia chilonis*^28^. A ribonuclease Oy-like found in the venom of the ectoparasitoid *Pachycrepoideus vindemmiae* may be related to the cleavage of host RNA to face host defensive reactions^20^. Inosine uridine-preferring nucleoside hydrolase-like is an enzyme capable of hydrolyzing nucleotides in nucleosides, with a preference for inosine and uridine^128^. It was found in the venom of the seed-parasitic wasp, *Megastigmus spermotrophus*^129^ and in the venom of the endoparasitoids *Leptopilina boulardi* and *L. heterotoma*^26^. However, its role in venom is still unknown. Another kind of deoxyribonuclease with predicted function in DNA degradation found in *T. sinensis* venom is plancitoxin. Plancitoxin, first detected in the venom of the starfish commonly known as crown-of-thorns, *Acanthaster planci*, is a DNAse II able to reduce cellular antioxidant level in response to high oxidative stress and induce hepatotoxic damage^130^. It was found in the venom gland of the endoparasitoids *M. demolitor*^109^ and *Lysiphlebus fabarum*^131^, although its role in parasitoid venom remains unknown. In *T. sinensis,* several nucleases were found by the proteomic approach: one ribonuclease oy-like, two endonuclease-like venom protein precursors, one inosine uridine-preferring nucleoside hydrolase-like precursor, one poly(U)-specific endoribonuclease homolog. Moreover, one endonuclease-like venom protein precursor and one plancitoxin were identified by the transcriptomic approach. Finally, one protein annotated as “uncharacterized protein” found by the proteomic approach can also be included in this group, as in its structure it contains a DDE_Tnp_4 domain, belonging to the endonuclease family.

### Protease inhibitors

*Serine protease inhibitors*, known also as serpin proteins, are common in the venom of different species, such as snakes, scorpions and wasps. Serpin proteins were found in the venom of endoparasitoids *L. boulardi*^132^, *A. ervi*^49^, *A. calandrae*^31^, *T. nigriceps*^18^ and *P. puparum*^133^ and in the venom gland of the endoparasitoid *Meteorus pulchricornis*^99^. Serpins can form permanent covalent complexes with target serine proteases^134^ and are involved in regulating the prophenoleoxidase cascade as well as blocking melanization process^133,135^. In *T. sinensis* venom, one serpin 5, a serine protease inhibitor 28Dc isoform X2 and a serine protease inhibitor 3/4 isoform X16 were identified using the proteomic approach. Moreover, a further protein annotated as “uncharacterized protein” found by the proteomic approach, was also included in this group, as it contains domains belonging to the Serpin superfamily.

*Kazal-type serine protease inhibition-like venom proteins* are involved in the inhibition of serine proteases, such as trypsin, chymotrypsin, and elastases. The inhibitory domain contains a specific peptide bond, which serves as a substrate for the cognate enzyme. The reactive site peptide bond is located within a loop whose conformation is identical in all Kazal inhibitors and all enzyme/inhibitor complexes. Similar domains, which are also present in follistatin and follistatin-like family members, play an important role in regulating specific tissues^136^. Four Kazal proteins are significantly expressed in the venom gland of the endoparasitoid *P. puparum*^29^, and two types were identified in the venom gland of the ectoparasitoid *A. calandrae*^31^, all with protease inhibitory activity. Kazal proteins may be involved in the immune response. Indeed, in *B. mori,* Kazal inhibitor proteins showed antimicrobial activity playing a putative protective role against invading pathogenic microorganisms^137^. Moreover, Kazal inhibitor proteins were also found in the saliva of blood-sucking insects, indicating a putative anticoagulant role^138^. Few studies on these proteins have been carried out in parasitoid wasps, unlike in other insects. Studies on Kazal-type serine protease inhibition-like proteins of *N. vitripennis* venom showed an inhibition of prophenoloxidase activation in parasitized *Musca domestica* hemolymph^139^. The same activity was observed in the endoparasitoid wasps *Venturia canescens*^140^. The prophenoloxidase system, one of the main components of the immune system in arthropods, activates specific humoral immune responses to non-self organisms through the melanization and damages their tissues. This process is mediated by the enzyme phenoloxidase, which is synthesized as the zymogen prophenoloxidase. The activation of prophenoloxidase in phenoloxidase is tightly regulated by the serine proteases cascade and by serpins^141^. Accordingly, it was proposed that Kazal-type serine protease inhibition-like venom proteins may play a role in suppressing host melanization^139^. One protein belonging to Kazal-type serine protease inhibition-like venom protein-1 and one protein belonging to Kazal-type serine protease inhibition-like venom protein-2 were identified in *T. sinensis* venom, using the transcriptomic approach.

Finally, an ovomucoid-like protein was identified by the proteomic approach. Ovomucoids, proteins found in the whites of eggs, are composed of three Kazal-type domains^142^. This protein, found in the venom of the snake *Bothriechis schlegelii*, has a putative function of a serine protease inhibitor, according to Kazal-type serine protease inhibitor role^142^.

*Cysteine-rich proteins* are protease inhibitors showing a distribution of cysteine residues similar to toxin proteins and serine protease inhibitors of insects and crustaceans^30^. Specifically, the Kunitz (KU) type motif was found in toxins from amphibians, snakes, spiders, cone snails and sea anemones^143^. In addition to the classical function of serine proteases inhibition, these cysteine-rich/KU venom proteins can block ion channels, which are essential for regulating various physiological processes such as blood coagulation, fibrinolysis and host defense, favoring the spread of parasitization events^30,45^. Moreover, cysteine-rich protease inhibitors could be involved in disrupting host immunity by inactivating the prophenoloxidase cascade, as the Kazal-type serine protease inhibition-like venom protein of the endoparasitoids *P. hypochondriaca* and *P. turionellae* and the ectoparasitoids *A. calandrae* and *N. vitripennis*^31,45,118,144^. In *T. sinensis* venom, using the transcriptomic approach, one cysteine-rich/KU venom protein and one cysteine-rich/pacifastin venom protein-2 were identified.

### Immune-related proteins

Several studies report that *N. vitripennis* venom inhibits host cellular immune response^145^. Venom proteins involved in the suppression of host defense are collectively named “Immune-related proteins”.

*Calreticulin* is a Ca^2+^-binding chaperone that was found in endoparasitoid *C. rubecola* venom, where it may compete with host calreticulin on the surface of hemocytes, acting as an antagonist of hemocyte activation in early encapsulation reactions. Calreticulin, indeed, seems to play an important role in encapsulation and phagocytosis, inhibiting hemocytes diffusion and suppressing host immune reaction^146,147^. Calreticulin has also been identified in the venom of other endoparasitoids such as *P. puparum*^148^ and *T. nigriceps*^18^, suggesting a role similar to that of *C. rubecola* calreticulin in hemocyte encapsulation. Beside that, by altering the intracellular calcium balance, calreticulin might affect the biological processes in which Ca^2+^ is involved, such as apoptosis, inflammation and the activation of hydrolytic enzymes. In *T. sinensis* venom, a calreticulin was identified by the proteomic approach.

*Calnexin* is another Ca^2+^-binding protein found in the venom of snake *Bothrops colombiensis*; together with calreticulin, calnexin may be involved in the process of toxin secretion^149^. In *T. sinensis* venom, calnexin was identified by the proteomic approach.

*Nucleobindin-2* is another protein that can be included in the Ca^2+^-binding protein group. In mammals, nucleobindin 2 is the precursor of a DNA- and calcium-binding protein, nesfatin-1, that seems to be associated with brain changes in stress situations. Nucleobindin-2 was reported in the venom of *A. mellifera*^102^, in the venom of *Ornithorhynchus anatinus* platypus, a venomous monotreme^150^, and in the venom of the snake *Bothrops jararaca*^151^, in which it could induce excitotoxicity, that is the alteration of nerve cells by excessive neurotransmitter stimulation^151^. In *T. sinensis* venom, Nucleobindin-2 was identified by the proteomic approach.

*Complement component 1q (C1q)* is a key protein in the classical complement pathway and represents the joining link between the acquired and the innate immune response^152^. As the human C1q domain directly interacts with lipopolysaccharides from gram-negative bacteria^153^, it is possible that this protein plays a role in opsonizing molecules^30^. This protein was also found in *A. mellifera* venom^154^. In *T. sinensis* venom, one C1q-like venom protein was identified through the transcriptomic approach.

### Recognition/binding proteins

*Odorant binding proteins* (OBPs) are a group of small globular and soluble polypeptides highly concentrated in olfactory organs as nasal mucus and tears in vertebrates^155^ and sensillar lymph in insect sensilla^156^. In invertebrates, they are characterized by a pattern of six conserved cysteine residues, paired in three disulphide bridges^157^. Beside the general OBP-like (GOBP-like) proteins in *N. vitripennis* venom^30^, OBPs were found in the venom of the endoparasitoids *P. puparum*^158^, *L. heterotoma*^82^, *C. inanitus*^124^, in the fire ant *Solenopsis invicta*^159^ and in the giant ant *Dinoponera quadriceps*. In the giant ant, OBP-like proteins induced IgE antibody production in prey (insects, small birds, and mammals) and acted as a powerful allergy-inducing molecule^160^. Their possible role in Hymenoptera venom and in host-parasitoid interactions is not known yet, but OBPs were supposed to be involved in host selection and in the search for an appropriate substrate to oviposit. In addition to this classical chemosensory function, OBP-like proteins in venom may play a role in transporting hydrophobic molecules, such as components of the nourishment, ensuring nutrients to the parasitoid rather than the host^31,156^. In *T. sinensis* venom, eleven general OBP-like proteins were identified by the proteomic approach.

*Chemosensory proteins,* similarly to OBPs, are small soluble proteins mediating sensory perception in insects. Their function in parasitoid venom could be very similar to OBPs mediating host selection and carrying hydrophobic feed molecules for the parasitoid offspring^31,124^. Chemosensory proteins were found in the venom of the endoparasitoid *C. inanitus*^124^ and the ectoparasitoid *A. calandrae*^31^. In *T. sinensis* venom, one chemosensory protein was identified using the proteomic approach.

*Low-density lipoprotein receptors* have a central role in cholesterol and other lipoprotein metabolism^161^, even though they are uncommon among venom proteins and their role in envenomation is still unknown. Low-density lipoprotein receptors were described in the venom of parasitoids, such as in *N. vitripennis*^30^ and *P. puparum*^27^ and the spider *Latrodectus hesperus*^162^. An example of this protein is PH-4 (neuropeptide prohormone-4) of *Profundiconus* cone snail genus. This protein, found also in the venom of other sea snails, has a mature sequence and a precursor-related peptide containing low-density lipoprotein receptor A domain^163^. Another example is PS1 (peptidase S1) from the crustacean *Xibalbanus tulumensis*, which also contains a low-density lipoprotein receptor A domain. This domain allows PS1 to bind lipoproteins, while the PS1 domain facilitates their digestion. These proteins are hypothesized to facilitate the interaction with lipoproteins of the prey/host to create a substrate for predators and parasitoids^164^. In *T. sinensis* venom, two low-density lipoprotein receptors were identified using the proteomic approach and one using the transcriptomic one.

*Apolipophorins,* which belong to apolipoprotein family and are involved in lipid transport processes in the insect hemolymph^165^, play also an important role in immunity, programmed cell death and the detoxification of lipopolysaccharide endotoxins^166^. They were found in venom of *A. mellifera* and *B. pascuorum* wasps and in predatory ant *O. monticola*^90,165,167^. In *T. sinensis* venom, two apolipophorins were identified using the proteomic approach. Moreover, this group of apolipophorins may include one protein annotated as “hypothetical protein”, as it contains an apolipophorin domain in its structure.

*Insulin-like growth factor-binding proteins* are able to bind insulin-like growth factors and allow their transport to target tissues, where they promote cell growth, proliferation, differentiation and survival^168^. These proteins were identified in the venom of the scorpion *Tityus stigmurus*^169^, in venom glands of the spider *Cupiennius salei*^170^ and in cobra venom, in which it seems to be related to apoptosis induction^171^. In *T. sinensis* venom, an insulin-like growth-factor-binding protein was identified using the proteomic approach.

### Glutathione metabolism

*γ-Glutamyl cyclotransferase* proteins are involved in glutathione metabolism. This enzyme was found in transcripts of the venom gland in the ectoparasitoid *A. calandrae*^31^ and in the venom of *M. spermotrophus*^129^. The function of this protein in the parasitoid venoms is still unknown. However, glutathione is fundamental in regulating homeostasis in the cell, and its alteration could result in oxidative stress and apoptosis, as reported for the γ-Glutamyl transpeptidase like venom protein identified in *A. ervi* venom^13,49^. In *T. sinensis* venom, one γ-glutamyl cyclotransferase-like venom protein was identified using the transcriptomic approach.

### Oxidases

*Laccases*, which belong to a group of proteins collectively known as multicopper oxidases, were supposed to play an important role in insect cuticle sclerotization^172^. During this extracellular process, cuticular proteins are cross-linked into a matrix as result of oxidative and nucleophilic reactions of catechols to their corresponding quinones^173^. This enzyme was found also in the venom of both the ectoparasitoid *N. vitripennis*^30^ and the endoparasitoid *P. hypochondriaca*^118^. In *T. sinensis* venom, one laccase was identified by the transcriptomic approach.

### Dehydrogenases

*Alcohol dehydrogenases* are oxidoreductase enzymes that catabolize otherwise toxic alcohols. They are not common in venom; to the best of our knowledge, this enzyme was previously identified exclusively in the venom of *A. mellifera*^174^ and in the venom of scorpion species *Leiurus abdullahbayrami*^175^. In *T. sinensis* venom, one alcohol dehydrogenase was identified by the proteomic approach.

### Isomerases

*Peptidyl-prolyl cis/trans isomerases* are enzymes that catalyze the cis-to-trans isomerization around proline, allowing proteins to fold into their correct conformation. In nature, proline is the only amino acid existing in cis and trans isomerization form; correct protein folding is often not possible when a proline peptide bond is in the incorrect configuration, and proper isomerization is necessary^176^. Peptidyl-prolyl cis/trans isomerases were identified in the venom gland of predatory marine cone snails *Conus novaehollandiae*, where they facilitate the in vitro folding of conotoxins^177^. This enzyme was also identified in the venom of the endoparasitoids *C. chilonis*^28^ and *T. nigriceps*^18^, in the venom of the ectoparasitoid *B. nigricans*^32^ and in the venom of the predatory ant *O. monticola*^90^ and the snake *C. durissus terrificus*^92^. Although the specific function of these enzymes in parasitoid venom remains to be determined, they may be involved in the folding of toxin peptides, as it occurs in other venomous organisms^177^. In *T. sinensis* venom, an isomerase, annotated as peptidyl-prolyl cis–trans isomerase 5-like, was identified by the proteomic approach.

*FK506-binding proteins* are immunophilins that bind immunosuppressive drugs such as FK506, rapamycin and cyclosporin A^178^. They often have peptidyl-prolyl cis–trans isomerase activity^179^. This enzyme was found in venom of the endoparasitoid *C. chilonis*^28^. In *T. sinensis* venom, one isomerase, annotated as FK506-binding protein, was identified by the proteomic approach.

*Protein disulfide-isomerase A3* is a molecular chaperone, supporting folding and processing of glycoprotein after their synthesis in the endoplasmic reticulum^180^. It was identified in the venom of the endoparasitoid *P. puparum*^27^, although its role in venom is still unknown. In *T. sinensis* venom, one isomerase, annotated as protein disulfide-isomerase A3, was identified by the proteomic approach.

### Heat shock proteins (HSPs)

*Endoplasmins* are molecular chaperones belonging to the HSP 90 family and involved in the final processing and export of secreted proteins. They may also play a role in the stabilization of other proteins. They were found in the venom of the endoparasitoid *A. ervi*, and, according to HSP functions, they may help protect parasitoid proteins during secretion and transport in host cells^49^. The same protein was identified in venom of the endoparasitoids *P. lounsburyi*, *P. concolor*^93^ and *C. cunea*^83^ and snake *Crotalus adamanteus*^181^. In *T. sinensis* venom, a heat shock protein, annotated as endoplasmin, was identified by the proteomic approach.

*Heat shock 70 kDa,* molecular chaperones involved in the folding of other proteins, were found in venom of the Hymenoptera wasps *Apoica pallens*^182^ and *Polybia paulista*^183^, and in venom of the ant *Neoponera villosa*^184^. These proteins also occur in the venom glands of bees^165^ and in venom of the endoparasitoid *T. nigriceps*^18^. In *T. sinensis* venom a heat shock protein, annotated as generic heat shock 70 kDa, was identified by the proteomic approach.

### Other proteins

In addition to the previously described categories, other proteins were identified, difficult to categorize but similar to known proteins.

*Allergens 3 and 5* are allergen proteins. Mostly studied and characterized in humans, they are also found in several wasp and ant species^130,185^. Venom allergen 3-like and allergen 5 were found in the venom of the ectoparasitoid *A. calandrae*^31^, allergen 3 in the venom of the endoparasitoid *P. puparum*^27^, allergen 5 in the venom of the endoparasitoids *T. nigriceps*^18^, *C. inanitus*^124^ and the ectoparasitoid *B. nigricans*^32^. *T. nigriceps* venom allergen 5-protein contains a sperm-coating protein (SCP)-like extracellular protein domain, that may function as endopeptidase. This protein might be involved in protein proteolysis and tissue degradation by the parasitoid^18^. In *T. sinensis* venom, six allergen proteins annotated as allergens 3 and 5 have been identified, two of which by the proteomic approach and four by the transcriptomic one. These proteins are very similar to *N. vitripennis* antigen 5-like proteins, which is also one of three major allergenic proteins found in the venom of *Vespula*, *Vespa* and *Dolichovespula*^100^.

*Major royal jelly proteins* are proteins involved in the development of bee larvae. MRJP7 is highly expressed in nurse bees and bees that feed the worker and the queen with jelly secreted from specific glands^186^. Moreover, proteins very similar to MRJP8 and 9 were also identified as components of honeybee venom^165,186^ and of the venom of parasitoids, such as the endoparasitoid *P. puparum*^27^ and the ectoparasitoid *P. vindemmiae*^20^, although their function in parasitoid venom is still unknown^165,187^. Recently it was hypothesized that they could be allergens^188^ or proteins related to storage of nutrients^20^. In *T. sinensis* venom, one protein annotated as major royal jelly protein was identified by the proteomic approach. Moreover, a protein annotated as “Uncharacterized protein” found by the proteomic approach could also be included in this group, as it contains a major royal jelly protein domain in its structure. The same protein was found in the ectoparasitoid *P. vindemmiae* venom^20^.

*Adipocyte plasma membrane-associated protein* was previously identified in the endoparasitoid *Tetrastichus brontispae*. In this study, the protein was found on the surface of the parasitoid egg and it was supposed to be involved in evading the host immunity response and protecting the egg during the early parasitoid stages, in association with a lipophorin protein^189^. The adipocyte plasma membrane-associated protein was very similar to hemomucin, an O-glycosylated surface mucin found on the extraembryonic membrane of many parasitoid eggs that may allow the embryo to evade the host encapsulation reaction^190^. Therefore, this protein could have protective properties in host-parasitoid systems. In *T. sinensis* venom, one protein annotated as adipocyte plasma membrane-associated protein was identified by the proteomic approach.

*Ferritin* is an intracellular protein that carries and stores iron. This protein is found in the venom of scorpion *Centruroides vittatus*^191^ and the endoparasitoids *M. aethiopoides* and *Microctonus hyperodae*^105^ and in venom gland of the endoparasitoid *M. pulchricornis*^99^, but its role is still unknown. In *T. sinensis* venom two proteins annotated as ferritin were identified by the proteomic approach.

*Lachesins* belong to the disintegrin family, typically of viper venoms, and act as potent inhibitors of platelet aggregation and integrin-dependent cell adhesion^192^. Lachesin was characterized in snake *Lachesis muta* venom^193^. Although this protein is not found in parasitoid venom, it could be involved in defence against host immunity responses. In *T. sinensis* venom one protein annotated as lachesin was identified by the proteomic approach.

*Mesencephalic astrocyte-derived neurotrophic factor* is an endoplasmic reticulum stress-inducible protein, originally identified as protein protecting rat dopaminergic neurons in vitro and prevents neuron degeneration in Parkinson's disease^194^. It was found in venom of endoparasitoid *C. chilonis*, in which probably it is involved in cell protection from endoplasmic reticulum stress^28^. In *T. sinensis* venom one protein, annotated as mesencephalic astrocyte-derived neurotrophic factor, was identified by the proteomic approach.

*Vitellogenin* is a protein involved in lipid transport from ovarian follicle cells to oocytes, providing nutrition during embryogenesis and playing a role as egg yolk protein precursor in the ovaries^195^. Despite its main role, it is also component of venom of Hymenoptera, such as *A. mellifera* and *Vespula vulgaris* in which it represents one of the allergens, with its IgE-reactive allergenic properties^196^, and *A. cerana* in which it was hypothesized that could be involved in response to microbial infection and oxidative stress, ensuring protection to DNA against ROS^197^. Indeed, it could be also considered an antimicrobial and antioxidant agent^197^. It was also found in venom of the ant *O. monticola*^134^ and in venom of the ectoparasitoid *A. calandrae*^31^. In *T. sinensis* venom a protein annotated as vitellogenin was identified by the proteomic approach.

*Agatoxins* are polyamine and peptide toxins isolated from spider and scorpion venoms^170,198^. Their mechanism of action led to the inactivation of several ion channels, causing neurotoxic effects^199,200^. Agatoxin was found also in the venom of the giant ant *D. quadriceps*^201^ and in the venom gland transcriptome of solitary and social wasps, such as *Vespa velutina*^202^. In *T. sinensis* one u8-agatoxin-ao1a-like isoform 1 was identified exclusively by the transcriptomic approach. To the best of our knowledge, the *T. sinensis* venom is the only one containing a putative agatoxin among parasitoid venoms. Because agatoxins are strongly involved in blocking ion channels, their action in parasitic syndrome could be strictly related to prey paralysis, that is one of the main effects of ectoparasitoid wasps attack^12^.

### Unknown and hypothetical proteins

We found different proteins that are not associated with known proteins, but they are very similar to the same unknown *N. vitripennis* proteins; they are named from “**venom protein A**” to “**venom protein Z**”.

In *T. sinensis* we found:

- venom protein D (one protein) identified by the transcriptomic approach, also found in the venom of *P. puparum*^27^ and *A. calandrae*^31^;
- venom protein F (one protein) identified by the transcriptomic approach, also found in the venom of the wasps *M. spermotrophus*^129^, *Megaphragma amalphitanum, Ceratosolen solmsi,* the endoparasitoid *Trichogramma pretiosum*^203^, and *A. calandrae*, in this case with a putative role in actin polymerization and in transcription regulation of cholesterol and fatty acid homeostasis^31^;
- venom protein L (one protein) identified by the transcriptomic approach, also found in the venom of *P. puparum*^27^, *A. calandrae*^31^ and *P. vindemmiae*^20^;
- venom protein N (one protein) identified by the transcriptomic approach, also found in the venom of *A. calandrae*^31^ and *P. vindemmiae*^20^;
- venom protein O (one protein) identified by the transcriptomic approach also found in the venom of *P. puparum*^27^ and *A. calandrae*, in this case with a putative role of OBP^31^;
- venom protein Q (one protein) identified by the transcriptomic approach, also found in the venom of *Nasonia giraulti*^114^ and *A. calandrae*, in which a seryl-tRNA synthetase domain was detected^31^;
- venom protein R (one protein) identified by the proteomic approach, also found in the venom of *P. puparum*^27^, *N. giraulti*^114^, *M. amalphitanum, C. solmsi, T. pretiosum*^203^, *Tetrastichus brontispae*^204,205^ and *M. spermotrophus*^129^. RNA sequencing performed on abdomen tissue in *Ischnura elegans* also revealed a venom protein r-like, a toxin and hemolymph juvenile hormone binding protein, that regulates embryogenesis, larva development and reproductive maturation^206^.
- venom protein T (one protein) identified by the transcriptomic approach, also found in the venom of *A. calandrae*^31^ and *N. giraulti*^114^.
- venom protein U (two proteins) identified by the proteomic approach, also found in the venom of *P. puparum*^27^, *A. calandrae*^31^ and *P. vindemmiae*^20^;
- venom protein V (one protein) identified by the transcriptomic approach, also found in the venom of *P. vindemmiae*^20^ and *A. calandrae*, in which a chaperone_ClpB domain was detected^31^. Chaperone ClpBs from several microorganisms are essential for survival under severe stress conditions^207^.

In addition to the above-mentioned proteins, a group of “**uncharacterized proteins”** and a group of “**hypothetical proteins**” were also found by the proteomic approach. A hypothetical protein is a protein whose existence has been predicted since it derives from an ORF, but there is no experimental evidence of translation^208^. The identification of these proteins in venom by the proteomic approach is an experimental evidence of their expression, but their characterization and their role remain unknown. In *T. sinensis* venom, we identified two proteins similar to hypothetical protein LOC100679659 isoform 1 and uncharacterized protein LOC100118367 of *N. vitripennis*, also found in the venom of *P. puparum*^27^. The analysis of the structure of uncharacterized protein LOC100113619 led to the identification of a SWVC domain (single-domain von Willebrand factor type C). Proteins characterized by this domain were also identified in the venom of the spider *Pamphobeteus verdolaga*^209^ and in the venom gland transcriptome of the scorpions *Hadrurus spadix*^208^, *Centruroides hentzi*^211^ and *Paravaejovis schwenkmeyeri*^212^. In the structures of uncharacterized protein LOC106783674 isoform X2 and uncharacterized protein LOC108911535 isoform X1, a DUF4803 domain (domain of unknown function) was identified. Some molecules containing this domain were found in venom of the endoparasitoids *P. lounsburyi* and *P. concolor*^93^ and the ectoparasitoid *B. nigricans*^32^. DUF protein families are still functionally uncharacterized. Overall, in *T. sinensis* venom, sixteen uncharacterized proteins and three hypothetical proteins were identified by the proteomic approach. According to the functional domains identified through the BLASTp software (https://blast.ncbi.nlm.nih.gov/Blast.cgi), some of the uncharacterized/hypothetical proteins can be included in the previous groups, as already described.

Further investigation will provide more information about these proteins.

## Conclusions

The integrated transcriptomic and proteomic approach used to analyze *Torymus sinensis* venom and the accompanying analysis, using a transcriptomic approach, provides an overview of venom’s major protein components, in order to understand the mechanism underlying the complex host/parasitoid interaction. The study of endo- and ectoparasitoid venoms, using these approaches, is the starting point for detailed knowledge of the molecular biology, evolution and effects of venom proteins in the host/parasitoid interactions. Indeed, although general physiological effects of Hymenoptera parasitoid venoms have been recorded, their exact composition is not completely known, also considering the high number of species and the differences among them. Specifically, in *T. sinensis* venom, a large number of proteins was identified, involved both in inhibition of the host immune system and in providing nutrients to the parasitoid progeny. Although additional in vivo studies are needed, this first view of venom protein components confirms how venom, combined with other maternal factors, helps ensure the success of parasitism.

## Materials and methods

### Insect rearing

*Torymus sinensis* adults were reared from galls of *Dryocosmus kuriphilus* collected in 26 sites within Cuneo province (NW Italy), where 40% of the forestry area is covered by sweet chestnut, *Castanea sativa* Mill. (Eudicots: Fagaceae) and nearly 10% by *Quercus* *spp*.^213^. This area has been widely infested by *D. kuriphilus* and includes the initial sites of *T. sinensis* release in Europe, performed in 2005. Chestnut trees were sampled from both mixed forests and chestnut orchards. Galls were randomly collected by hand from low branches and with the aid of lopping shears from the medium–high canopy, according to a previously described method^36,214^. They were separated from any non-gall plant material to avoid contamination by other insects not associated with the galls and then stored in containers outdoors in cardboard rearing boxes. Up to 2000 galls were kept in a container. Rearing boxes were checked once per week until the emergence of the first parasitoid wasp, then parasitoids were collected daily and their date of emergence recorded. All *T. sinensis* wasps were removed using an entomological pooter, then stored in 99% ethanol. All specimens were divided by sex by observing their morphological characters and then sent to the University of Basilicata’s laboratory for the subsequent analysis.

### Venom gland collection

Before venom glands were collected, *T. sinensis* adult females were briefly (a few minutes) anesthetized on ice and subsequently placed in a phosphate-buffered saline solution (1X PBS) (Sigma, St. Louis, MO, USA) in a Petri dish (Sigma, St. Louis, MO, USA). A stereo microscope (Nikon, Tokyo, Japan) was used for dissections. The entire reproductive apparatus was removed with a pair of forceps and placed in a drop of 1X PBS solution. Subsequently, the venom glands were isolated and placed in a centrifuge tube (Eppendorf, Hamburg, Germany) containing TRI Reagent (Sigma, St. Louis, MO, USA) for the following RNA extraction and on slides for the subsequent microscope observations (Sigma, St. Louis, MO, USA).

### Light microscopy

Venom glands were carefully transferred onto slides and fixed at room temperature for 10 min with 4% formaldehyde pH 6.9 (Carlo Erba, Milano, Italy). Then samples were observed using a Nikon Eclipse 80i (Instruments Europe, Amsterdam, The Netherlands) at 10X magnification. Images were recorded by a Nikon Digital Sight Ds-Fil camera (Nikon Instruments Europe).

### RNA extraction

Total RNA was extracted from 360 venom glands, collected as previously described, using TRI Reagent following the manufacturer's instructions (Sigma, St. Louis, MO, USA). A Turbo DNase (Ambion Austin, TX, USA) treatment was carried out to eliminate any contaminating DNA. The DNase enzyme was then removed and the RNA was further purified using the RNeasy MinElute Clean up Kit (Qiagen, Venlo, The Netherlands) following the manufacturer's protocol and eluted in 20 µl of RNAse free water (Ambion Austin, TX, USA). RNA integrity was verified on an Agilent 2100 Bioanalyzer using the RNA Nano chips (Agilent Technologies, Palo Alto, CA), and RNA quantity was determined by a Nanodrop ND1000 spectrophotometer (Thermo Fisher Scientific, Waltham, MA, USA).

### RNAseq data generation and de novo transcriptome assembly

Tissue-specific transcriptome sequencing of the RNA sample was performed with poly(A) + enriched mRNA (New England Biolabs, Ipswich, MA, USA) fragmented to an average of 240 nucleotides. Sequencing was carried out by the Max Planck Genome Center (http://mpgc.mpipz.mpg.de/home/) using standard TruSeq procedures on an Illumina HiSeq2500 sequencer (Illumina., San Diego, CA, USA), generating ~ 42 Mio paired-end (2 × 100 bp) reads for the venom tissue sample. Quality control measures, including the filtering of high-quality reads based on the score given in fastq files, removal of reads containing primer/adaptor sequences and the trimming of read length, were carried out using CLC Genomics Workbench v9 (http://www.clcbio.com). The de novo transcriptome assembly was carried out using CLC Genomics Workbench v9 with standard settings and two additional CLC-based assemblies with different parameters and then selecting the presumed optimal consensus transcriptome, as previously described^215^. The transcriptome was annotated using BLAST, Gene Ontology and InterProScan with Blast2GO Pro version 4.1^216^*.* For BLASTx searches against the non-redundant NCBI protein database (nr database), up to 20 best NR hits per transcript were retained, with an E-value cutoff of ≤ 1E-3 and a minimum match length of 15 amino acids. To optimize annotation of the obtained data, we used GO slim, a subset of GO terms that provides a high level of annotations and allows a global view of the result. The functions "Gene Ontology Graphs" and "Enrichment Analysis" (Fisher's exact test) was used as part of OmicsBox (1.4.11) to identify the distribution of gene ontology (GO) terms as well as overrepresentation of GO terms among the *T. sinensis* venom protein dataset relative to the complete reference dataset (*T. sinensis* transcriptome assembly). The GO-enriched bar charts were reduced to display only the most specific GO terms by removing parent terms representing existing child terms using the function “Reduce to most specific terms” implemented in OmicsBox. A GO term was considered significantly enriched if the p-value corrected by false discovery rate control (FDR) was less than 0.05. To assess transcriptome completeness, we performed a BUSCO (Benchmarking Universal Single-Copy Orthologs; http://busco.ezlab.org) analysis by comparing our assembled transcriptome against a set of highly conserved single-copy orthologs. This was accomplished using the BUSCO v3 pipeline^217^, comparing the predicted proteins of the *T. sinensis* transcriptome to the predefined set of 1658 Insecta single-copy orthologs from the OrthoDB v9.1 database. This resulted in 91.9% complete and 5% missing BUSCO genes for the venom gland transcriptome assembly. The assembled and annotated venom gland transcriptome was used to generate a custom-made protein database. The six reading frames of the 22,874 nucleotide sequences were translated in their corresponding amino acid sequences by SEQtools software (http://www.seqtools.dk/).

### Digital gene expression analysis

Digital gene expression analysis was carried out by using CLC Genomics workbench v9 (http://www.clcbio.com) to generate BAM (mapping) files and QSeq Software (DNAStar Inc., Madison, WI, USA) to remap the Illumina reads onto the reference transcriptome, and finally by counting the sequences to estimate expression levels, using previously described parameters for read-mapping and normalization^218^. In particular, the expression abundance of each contig was calculated based on the reads per kilobase per million mapped reads (RPKM) method^218^, using the formula: RPKM (A) ¼ (10,00,000 _ C _ 1000)/(N _ L), where RPKM (A) is the abundance of gene A, C is the number of reads that uniquely aligned to gene A, N is the total number of reads that uniquely aligned to all genes, and L is the number of bases in gene A.

### Quantitative real time PCR (qPCR)

The relative expression in *T. sinensis* venom gland and female body deprived of venom gland of 10 genes selected among those reported in Table 1 was evaluated by a quantitative real time PCR (qPCR) (ABI PRISM R 7500 Fast Real-Time PCR System Thermal Cycler—Applied Biosystems, Foster City, CA, USA). Glyceraldehyde-3-phosphate dehydrogenase (GAPDH) and Beta-tubulin have been chosen as reference genes for normalization of qPCR data. Primers were designed using Primer3web (version 4.1.0) (Supplementary Table S11). PCR amplifications, with 30 ng of cDNA, were carried out using GoTaq qPCR Master Mix (Promega, Madison, WI, USA), following the manufacturer's instructions. Cycling programme was: 2 min at 95 °C, 40 cycles of 15 s at 95 °C and 1 min at 58 °C. Three technical replicates and three biological replicates were performed for each reaction. To evaluate gene expression levels, relative quantification was performed using equations described by Liu et al.^219^, based on PCR amplification efficiencies of reference and target genes. Amplification efficiency of each gene was calculated according to the equation E = 10^–1/S^—1 (S is the slope of the curve derived from three serial tenfold cDNA dilutions)^220^. The efficiencies of the amplicons were approximately equal. Quantification analysis of amplification was performed using the comparative ΔΔCt method^221^. Data were expressed as mean ± SEM (standard error of mean) of independent biological replicates and were compared by the Unpaired t-test using GraphPad Prism 6.00 software for Windows (GraphPad Software, La Jolla, CA, USA).

### Phylogenetic analyses of *T. sinensis* trypsins

We inferred the species-specific diversification patterns of putative trypsins identified in the *T. sinensis* venom gland transcriptome in phylogenetic analyses. We used all predicted serine protease sequences from *T. sinensis* as query to search for homologs in the NCBI nr protein database using Blastp (E-value threshold of 10–5), identified the top 50 best Blast hits and removed redundant entries. Next, we removed partial sequences with less than 50% of the typical protease length. The corresponding protein sequences were aligned using MAFFT implemented in Geneious (v11.0.4) with FFT-NS-i × 1000 algorithm and BLOSUM62 scoring matrix. The alignments were trimmed manually. Maximum-likelihood phylogenetic trees were constructed in FastTree implemented in Geneious (v11.0.4) with 1000 ultrafast bootstrap replicates for the full dataset. The tree was visualized and processed in Figtree v1.4.4 (http://tree.bio.ed.ac.uk/software/figtree). Numbers next to the tree branches indicate the support values.

### Collection of venom and SDS-PAGE electrophoresis

Wasps previously anesthetized on ice were submerged in 1 × PBS solution (Sigma, St. Louis, MO, USA) and their venom apparatus (venom glands and reservoir) was isolated. Each reservoir was gently opened with a dissecting needle in a drop of water (ratio 1 µl of water: 1 reservoir). The resulting crude extract was centrifuged at 5000*g* for 5 min at 4 °C, and the supernatant was used for electrophoretic analysis. For the proteome analysis, the venom from 30 *T. sinensis* females was collected for a total of 150 µg of protein. Protein quantity was measured using the Bradford method, with bovine serum albumin as standard^222^. An aliquot of venom proteins was loaded on a 12,5% polyacrylamide running gel on a Bio Rad Electrophoresis Cell Mini Protean II (Biorad, Hercules, CA, USA). After the run, the gel was stained with colloidal Coomassie Blue G-250 (Sigma, St. Louis, MO, USA) for 1 h and the excess dye was removed by washing in deionized water for 12 h.

### In situ protein digestion

After electrophoresis and staining, whole lanes were cut in 35 bands. The bands were in situ hydrolysed by trypsin as reported in Medugno et al.^223^. Briefly, gel bands were destained by washes in acetonitrile (ACN) (Honeywell, Charlotte, NC, USA), and 50 mM ammonium bicarbonate (NH4HCO3). Cysteine residues involved in disulphide bridges were reduced by 10 mM of dithiothreitol (Sigma, St. Louis, MO, USA) in NH4HCO3 for 45 min at 56 °C and then alkylated in 55 mM iodoacetamide (Sigma, St. Louis, MO, USA) for 30 min. The excess reagents were finally removed by washing with ACN and 50 mM NH4HCO3 alternatively. The dehydrated gel bands were then treated overnight with trypsin and peptide mixtures extracted in 0.2% HCOOH and ACN. The obtained mixtures were vacuum dried by a Savant SpeedVac System (Thermo Fisher Scientific, Waltham, MA, USA).

### LC–MS/MS and protein identification

Each peptide mixture was resuspended in 10 μl of 0.2% HCOOH (Sigma, St. Louis, Missouri, USA) and analyzed by nanoLC–MS/MS on a LTQ Orbitrap mass spectrometer equipped with a nano HPLC system (Thermo Fisher Scientific, Waltham, MA, USA). After loading, the peptide mixture was first concentrated and desalted in the precolumn (C18 Easy Column L = 2 cm, ID = 100 mm, Thermo Fisher Scientific Waltham, MA, USA). Each peptide sample was then fractionated on a C18 reverse-phase capillary column (C18 Easy Column L = 20 cm, ID = 7.5 µm, 3 µm, (Thermo Fisher Scientific Waltham, MA, USA) working at a flow rate of 250 nl/min. The gradient used for peptide elution ranged from 5 to 95% of buffer B. Buffers A and B have the following composition:—buffer A, 2% ACN LC–MS grade and 0.2% HCOOH;—buffer B, 95% ACN LC–MS grade and 0.2% HCOOH. The MS/MS method was set up in a data-dependent acquisition mode, with a full scan ranging from 400 to 1800 m/z range, followed by fragmentation in CID modality of the top 10 ions (MS/MS scan) selected on the basis of intensity and charge state (+ 2, + 3, + 4 charges). An exclusion time of 40 s was applied to avoid the repetitive fragmentation of the same signals over a 40 s interval and to increase the number of fragmented peptides and, therefore, the number of available protein identification sequences. The raw files obtained from this analysis were used as inputs in the Andromeda search engine. The peak list generated was uploaded in Andromeda software and a research was performed using the “*T. sinensis* protein database.” These parameters were fixed: “trypsin” as an enzyme allowing up to 2 missed cleavages, carbamidomethyl as a fixed modification, oxidation of M, pyroGlu N-term Q, as variable modifications, 0.5 Da MS/MS tolerance, 10 ppm peptide tolerance. Scores used to evaluate the quality of matches for MS/MS data were higher than 10 for unmodified peptides, otherwise 40.

### Transcriptomic and proteomic data analysis

Putative venom proteins in the venom gland transcriptome were identified using a key-word approach: all proteins annotated with the word “venom” or “toxin” were selected. Proteins identified with the proteomic and the transcriptomic approach were analyzed using the Signal P 5.0 software (http://www.cbs.dtu.dk/services/SignalP/) in order to pick out those with signal peptide. Then, a second filter was applied, consisting of the alignment of the amino acid sequences of these proteins with venom proteins of *Nasonia vitripennis* identified by de Graaf et al. 2010^30^. The *N. vitripennis* database was used as reference because the top BLAST Hit Species Distribution showed a high level of matching with *T. sinensis*. The alignments were made using the software BLASTp (https://blast.ncbi.nlm.nih.gov/Blast.cgi?PAGE=Proteins). Most of these proteins have been updated in recent years, so the final amino acid sequences were identified in the NCBI protein database by using accession numbers or amino acid sequences.

## Supplementary Information

Supplementary Information
