# Supplementary material for: An integrated transcriptomic and proteomic approach to identify the main Torymus sinensis venom components
Source: Sci Rep. 2021 Mar 3;11:5032. doi: 10.1038/s41598-021-84385-5 (PMC7930282; doi:10.1038/s41598-021-84385-5)
Supplement: Supplementary file 1 — Supplementary Information [file 41598_2021_84385_MOESM1_ESM.docx]

**An integrated transcriptomic and proteomic approach to identify the main *Torymus sinensis* venom components**

Carmen Scieuzo^1,2#^, Rosanna Salvia^1,2#^, Antonio Franco^1,2^, Marco Pezzi^3^, Flora Cozzolino^4,5^, Milvia Chicca^3^, Chiara Scapoli^3^, Heiko Vogel^6^*, Maria Monti^4,5^, Chiara Ferracini^7^, Pietro Pucci^4,5^, Alberto Alma^7^, Patrizia Falabella^1,2*^.

1 Department of Sciences, University of Basilicata, Via dell'Ateneo Lucano 10, 85100, Potenza, Italy.

2 Spinoff XFlies s.r.l, University of Basilicata, Via dell'Ateneo Lucano 10, 85100, Potenza, Italy.

3 Department of Life Sciences and Biotechnology, University of Ferrara, Via L. Borsari 46, 44121, Ferrara, Italy

4 Department of Chemical Sciences, University Federico II of Napoli, Via Cinthia 6, 80126, Napoli, Italy

5 CEINGE Advanced Biotechnology, Via Gaetano Salvatore 486, 80126, Napoli, Italy.

6 Department of Entomology, Max Planck Institute for Chemical Ecology, Hans-Knöll-Straße 8, D-07745, Jena, Germany.

7 Department of Agricultural, Forest and Food Sciences, University of Torino, Largo Paolo Braccini 2, 10095, Grugliasco, Italy

#equally

*corresponding authors: [patrizia.falabella@unibas.it](mailto:patrizia.falabella@unibas.it); [hvogel@ice.mpg.de](mailto:hvogel@ice.mpg.de)


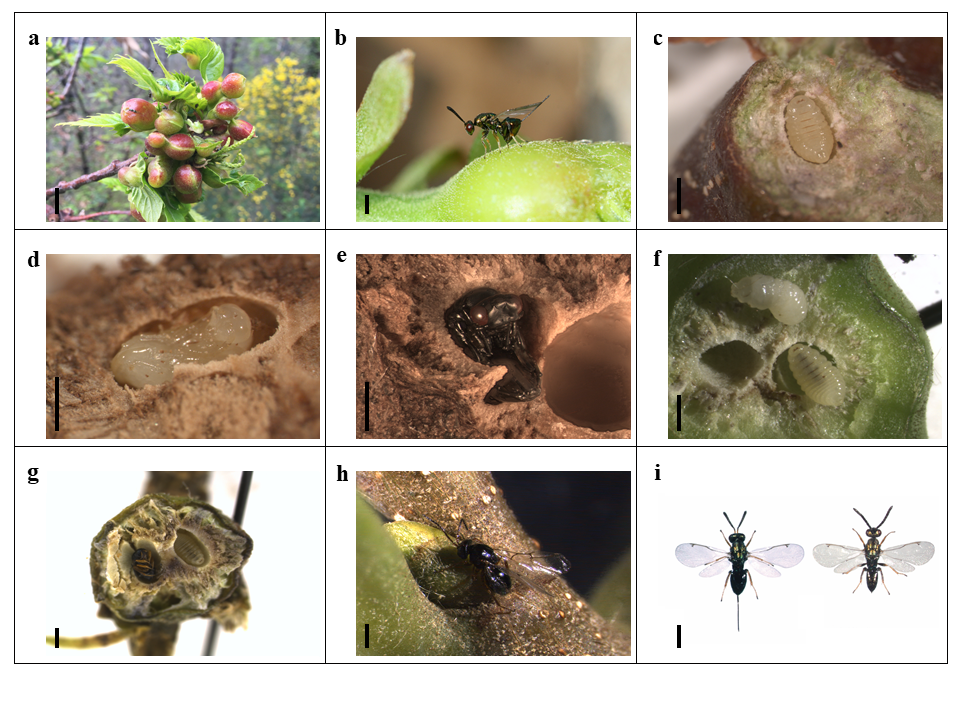


**Supplementary Figure 1: different stages of the parasitoid *Torymus sinensis* Kamijo (Hymenoptera: Torymidae) and *Dryocosmus kuriphilus* Yasumatsu (Hymenoptera: Cynipidae).** a) *T. sinensis* parasitizing galls of a sweet chestnut *Castanea sativa* Mill. (Eudicots: Fagaceae), infested by *D. kuriphilus*; b) *T . sinensis* parasitizing galls of a sweet chestnut (detail); c) *T. sinensis* mature larva; d) *T. sinensis* young pupa; e) *T. sinensis* mature pupa; f) comparison between a *D. kuriphilus* larva (above) and a *T. sinensis* larva (below) inside the chambers of a dissected gall g) Comparison between a *D. kuriphilus* adult (left) and a *T. sinensis* larva (right) inside the chambers of a dissected gall; h) *D. kuriphilus* adult; i) *T. sinensis* female (left) and male (right) adults. Scale bar = 1 cm (Figure 1a), scale bar = 1 mm (Figures 1b-i).

**
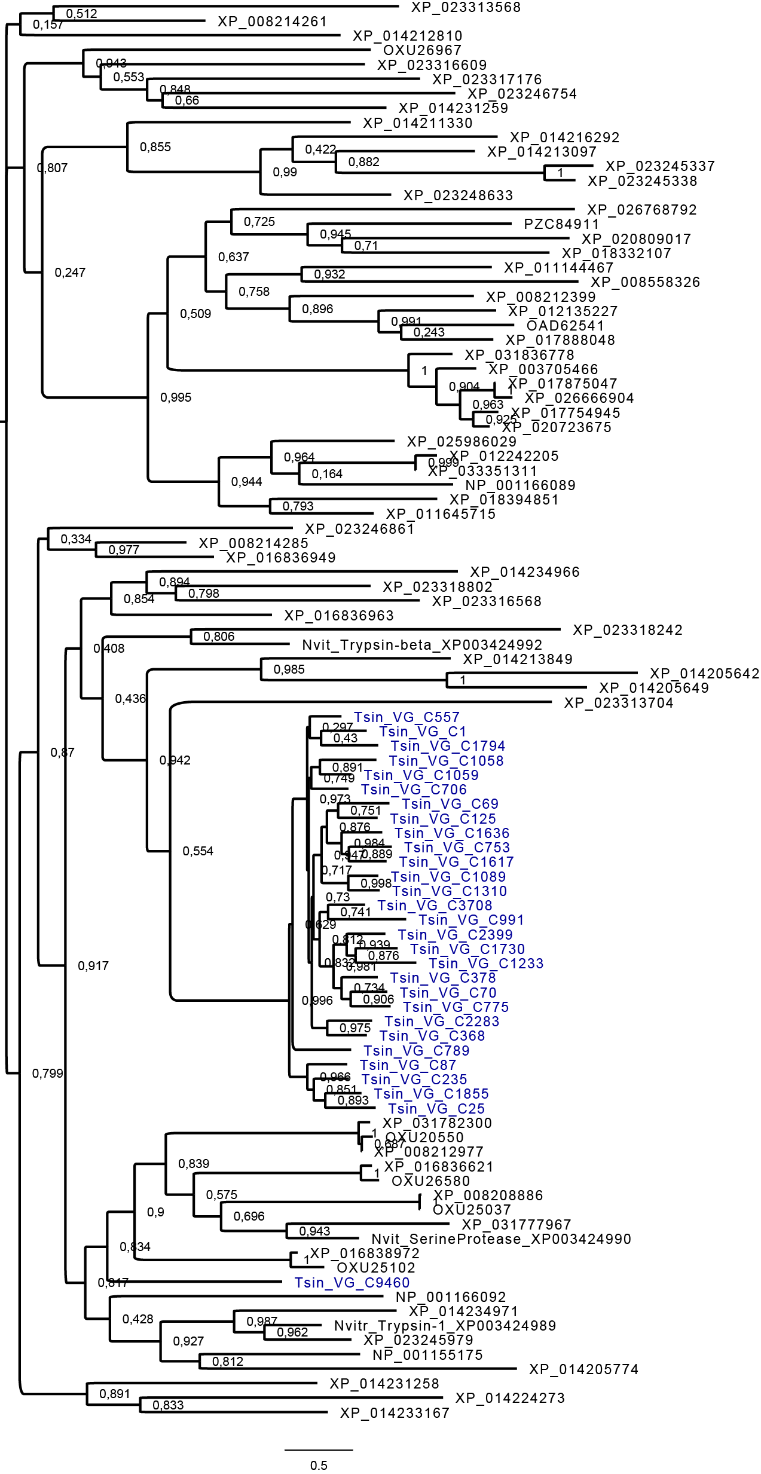
**

**Supplementary Figure 2: Diversification of trypsin-like sequences in *Torymus sinensis*.** Maximum-likelihood inferred phylogeny of all trypsin-like predicted protein sequences identified in the *T. sinensis* venom gland transcriptome and a subset of predicted insect trypsins. The expanded clade in *T. sinensis* is highlighted with blue letters. Bootstrap support values (1,000 replicates) are indicated on the corresponding nodes.

To have further confirmation of the venom nature of the 195 *T. sinensis* proteins identified with proteomic and transcriptomic approach, these proteins were aligned and compared to the identified venom proteins of *Nasonia vitripennis*, described in de Graaf et al*.*^30.^ The alignments, performed with the BLASTp software, allow to identified the following matches, also reported in Supplementary Tables 1, 2, 3:

- 10 protein match with 7 of 21 *N. vitripennis* proteins found by bioinformatic approach (Table S1^30^);

- 56 proteins matched with 16 of the 61 *N. vitripennis* proteins found by proteomic approach and provided with signal peptide (Table S2^30^); 2 of them, respectively identified as c1746 and c1835, matched also with proteins identified by de Graaf et al*.*^30^ through a bioinformatic approach (Table S1^30^), as the reported *N. vitripennis* proteins have the same accession number;

- 10 proteins match with 8 of the 15 *N. vitripennis* proteins found by proteomic approach, reported as not venomous proteins because of the absence of signal peptide (Table S3^30^); 1 of them, identified in *T. sinensis* as c3591, matches also with a protein identified by de Graaf et al*.*^30^ using exclusively a bioinformatic approach (Table S1^30^), as the *N. vitripennis* proteins have the same accession number.

Because of these last matches, we decided to reanalyse *N. vitripennis* protein reported by de Graaf et al.^30^ in Table S3 as “Non-secretory proteins discovered by a proteomic approach” using the Signal P 5.0 software and they resulted all provided with signal peptide, with the exception of actin. This incongruity could be related to usage of different software to analyse the signal peptide presence, as in de Graaf et al.^30^was used Signal P 3.0 Server while we used a most recent version of the same software.

| **Contig** | ***Torymus sinensis* protein** | ***Nasonia vitripennis* protein^30^** | **Query cover** | **E-value** | **Identity** |
| --- | --- | --- | --- | --- | --- |
| 1001 | Calreticulin precursor | [NP_001155151.1 calreticulin precursor [Nasonia vitripennis]](https://blast.ncbi.nlm.nih.gov/Blast.cgi#alnHdr_Query_42975) | 100% | 0.0 | 78.97% |
| 2736 | Chitotriosidase-1 isoform X1 | [NP_001155084.1 chitinase 5 precursor [Nasonia vitripennis]](https://blast.ncbi.nlm.nih.gov/Blast.cgi#alnHdr_Query_42976) | 86% | 5,00E-100 | 40.23% |
| 3012 | Serine protease persephone isoform X2 | [XP_001605133.1 PREDICTED: trypsin-1 [Nasonia vitripennis]](https://blast.ncbi.nlm.nih.gov/Blast.cgi#alnHdr_Query_42986) | 62% | 6,00E-31 | 31.73% |
| 3120 | Venom allergen 5-like | [NP_001155154.1 antigen 5-like protein 1 precursor [Nasonia vitripennis]](https://blast.ncbi.nlm.nih.gov/Blast.cgi#alnHdr_Query_42971) | 96% | 1,00E-72 | 47.37% |
| 6126 | Chymotrypsin-2-like | XP_001605133.1 PREDICTED: trypsin-1 [Nasonia vitripennis] | 90% | 5,00E-42 | 39.75% |
| 6382 | Serine protease 43 precursor | XP_001605133.1 PREDICTED: trypsin-1 [Nasonia vitripennis] | 89% | 1,00E-45 | 38.40% |
| 8578 | Venom allergen 5-like | [NP_001155154.1 antigen 5-like protein 1 precursor [Nasonia vitripennis]](https://blast.ncbi.nlm.nih.gov/Blast.cgi#alnHdr_Query_42971) | 92% | 3,00E-72 | 48.34% |
| 10799 | Putative odorant binding protein 36 | [NP_001155084.1 chitinase 5 precursor [Nasonia vitripennis]](https://blast.ncbi.nlm.nih.gov/Blast.cgi#alnHdr_Query_42976) | 40% | 2.9 | 50.00% |
| 11199 | Angiotensin-converting enzyme | [XP_001607198.1 angiotensin-converting enzyme [Nasonia vitripennis]](https://blast.ncbi.nlm.nih.gov/Blast.cgi#alnHdr_Query_42970) | 98% | 0.0 | 80.00% |
| 11592 | Serine protease 72 precursor | [XP_001605133.1 PREDICTED: trypsin-1 [Nasonia vitripennis]](https://blast.ncbi.nlm.nih.gov/Blast.cgi#alnHdr_Query_42986) | 94% | 3,00E-43 | 36.88% |

**Supplementary Table 1:*Torymus sinensis* venom proteins identified through the proteomic and transcriptomic combined approach aligned with *Nasonia vitripennis* protein in de Graaf et al.^30^ (Table S1. Venom proteins discovered by a bioinformatic approach).** In the table are reported: the contig number, the protein name, the *N. vitripennis* protein and some data concerning the alignment of the two proteins (query cover, E-value and identity). The alignments were performed with the software BLASTp (<https://blast.ncbi.nlm.nih.gov/Blast.cgi?PAGE=Proteins>).

| **Contig** | ***Torymus sinensis* protein** | ***Nasonia vitripennis* protein^30^** | **Query cover** | **E-value** | **Identity** |
| --- | --- | --- | --- | --- | --- |
| 1 | Trypsin-like | NP_001155175.1 serine protease precursor [Nasonia vitripennis] | 86% | 3,00E-46 | 33.80% |
| 4 | Serine protease 33 isoform X2 | NP_001155017.1 serine protease 33 precursor [Nasonia vitripennis] | 98% | 6,00E-78 | 46.72% |
| 25 | Trypsin-like | [NP_001155175.1 serine protease precursor [Nasonia vitripennis]](https://blast.ncbi.nlm.nih.gov/Blast.cgi#alnHdr_Query_43015) | 86% | 1,00E-43 | 33.57% |
| 69 | Trypsin-3-like | [NP_001155175.1 serine protease precursor [Nasonia vitripennis]](https://blast.ncbi.nlm.nih.gov/Blast.cgi#alnHdr_Query_43015) | 86% | 1,00E-15 | 22.84% |
| 70 | Trypsin-like | [NP_001155175.1 serine protease precursor [Nasonia vitripennis]](https://blast.ncbi.nlm.nih.gov/Blast.cgi#alnHdr_Query_43015) | 88% | 4,00E-37 | 30.85% |
| 87 | Trypsin-like | [NP_001155175.1 serine protease precursor [Nasonia vitripennis]](https://blast.ncbi.nlm.nih.gov/Blast.cgi#alnHdr_Query_43015) | 86% | 1,00E-24 | 28.98% |
| 106 | Carboxylesterase clade B, member 2 precursor | [NP_001155148.1 carboxylesterase clade B, member 2 precursor [Nasonia vitripennis]](https://blast.ncbi.nlm.nih.gov/Blast.cgi#alnHdr_Query_42990) | 99% | 6,00E-167 | 44.27% |
| 125 | Chymotrypsin-1-like | [NP_001155175.1 serine protease precursor [Nasonia vitripennis]](https://blast.ncbi.nlm.nih.gov/Blast.cgi#alnHdr_Query_43015) | 88% | 2,00E-43 | 31.49% |
| 157 | Venom protein u precursor | [NP_001155170.1 venom protein U precursor [Nasonia vitripennis]](https://blast.ncbi.nlm.nih.gov/Blast.cgi#alnHdr_Query_43045) | 100% | 1,00E-15 | 24.77% |
| 235 | Trypsin-like | [NP_001155175.1 serine protease precursor [Nasonia vitripennis]](https://blast.ncbi.nlm.nih.gov/Blast.cgi#alnHdr_Query_43015) | 87% | 2,00E-45 | 35.19% |
| 270 | Trypsin-like | [NP_001155175.1 serine protease precursor [Nasonia vitripennis]](https://blast.ncbi.nlm.nih.gov/Blast.cgi#alnHdr_Query_43015) | 94% | 2,00E-46 | 36.04% |
| 289 | Low-density lipoprotein receptor-related protein 2-like | [NP_001155040.1 low-density lipoprotein receptor-like venom protein precursor [Nasonia vitripennis]](https://blast.ncbi.nlm.nih.gov/Blast.cgi#alnHdr_Query_59729) | 11% | 0.30 | 42.86% |
| 368 | Trypsin-like | [NP_001155175.1 serine protease precursor [Nasonia vitripennis]](https://blast.ncbi.nlm.nih.gov/Blast.cgi#alnHdr_Query_43015) | 87% | 2,00E-45 | 33.79% |
| 378 | Chymotrypsin-1-like | [NP_001155175.1 serine protease precursor [Nasonia vitripennis]](https://blast.ncbi.nlm.nih.gov/Blast.cgi#alnHdr_Query_43015) | 93% | 3,00E-43 | 31.15% |
| 392 | Lipase 3-like | [NP_001154991.1 lipase A-like precursor [Nasonia vitripennis]](https://blast.ncbi.nlm.nih.gov/Blast.cgi#alnHdr_Query_43010) | 83% | 8,00E-53 | 33.51% |
| 557 | Trypsin-like | [NP_001155175.1 serine protease precursor [Nasonia vitripennis]](https://blast.ncbi.nlm.nih.gov/Blast.cgi#alnHdr_Query_43015) | 87% | 2,00E-49 | 34.49% |
| 603 | Venom protein Z precursor | [NP_001155169.1 venom protein Z precursor [Nasonia vitripennis]](https://blast.ncbi.nlm.nih.gov/Blast.cgi#alnHdr_Query_43050) | 99% | 4,00E-32 | 39.74% |
| 706 | Trypsin-like | [NP_001155175.1 serine protease precursor [Nasonia vitripennis]](https://blast.ncbi.nlm.nih.gov/Blast.cgi#alnHdr_Query_43015) | 86% | 6,00E-42 | 33.22% |
| 753 | Trypsin-like | [NP_001155175.1 serine protease precursor [Nasonia vitripennis]](https://blast.ncbi.nlm.nih.gov/Blast.cgi#alnHdr_Query_43015) | 87% | 1,00E-46 | 33.33% |
| 775 | Trypsin-like | [NP_001155175.1 serine protease precursor [Nasonia vitripennis]](https://blast.ncbi.nlm.nih.gov/Blast.cgi#alnHdr_Query_43015) | 88% | 4,00E-39 | 31.83% |
| 789 | Trypsin-like | [NP_001155175.1 serine protease precursor [Nasonia vitripennis]](https://blast.ncbi.nlm.nih.gov/Blast.cgi#alnHdr_Query_43015) | 85% | 5,00E-41 | 33.80% |
| 991 | Trypsin-like | [NP_001155175.1 serine protease precursor [Nasonia vitripennis]](https://blast.ncbi.nlm.nih.gov/Blast.cgi#alnHdr_Query_43015) | 86% | 4,00E-44 | 34.40% |
| 1049 | Venom serine carboxypeptidase isoform X1 | [NP_001155148.1 carboxylesterase clade B, member 2 precursor [Nasonia vitripennis]](https://blast.ncbi.nlm.nih.gov/Blast.cgi#alnHdr_Query_42990) | 11% | 0.67 | 42.31% |
| 1058 | Trypsin-like | [NP_001155175.1 serine protease precursor [Nasonia vitripennis]](https://blast.ncbi.nlm.nih.gov/Blast.cgi#alnHdr_Query_43015) | 63% | 6,00E-19 | 32.54% |
| 1059 | Trypsin-like | [NP_001155175.1 serine protease precursor [Nasonia vitripennis]](https://blast.ncbi.nlm.nih.gov/Blast.cgi#alnHdr_Query_43015) | 93% | 5,00E-49 | 33.97% |
| 1089 | Trypsin-like | [NP_001155175.1 serine protease precursor [Nasonia vitripennis]](https://blast.ncbi.nlm.nih.gov/Blast.cgi#alnHdr_Query_43015) | 86% | 2,00E-41 | 34.84% |
| 1233 | Trypsin-like | [NP_001155175.1 serine protease precursor [Nasonia vitripennis]](https://blast.ncbi.nlm.nih.gov/Blast.cgi#alnHdr_Query_43015) | 88% | 2,00E-37 | 31.54% |
| 1301 | Venom protein U precursor | [NP_001155170.1 venom protein U precursor [Nasonia vitripennis]](https://blast.ncbi.nlm.nih.gov/Blast.cgi#alnHdr_Query_43045) | 100% | 1,00E-34 | 33.49% |
| 1310 | Trypsin-like | [NP_001155175.1 serine protease precursor [Nasonia vitripennis]](https://blast.ncbi.nlm.nih.gov/Blast.cgi#alnHdr_Query_43015) | 86% | 4,00E-45 | 33.68% |
| 1617 | Trypsin-like | [NP_001155175.1 serine protease precursor [Nasonia vitripennis]](https://blast.ncbi.nlm.nih.gov/Blast.cgi#alnHdr_Query_43015) | 84% | 6,00E-42 | 31.18% |
| 1636 | Trypsin-like | [NP_001155175.1 serine protease precursor [Nasonia vitripennis]](https://blast.ncbi.nlm.nih.gov/Blast.cgi#alnHdr_Query_43015) | 87% | 1,00E-39 | 32.86% |
| 1651 | Venom carboxylesterase-6 | [NP_001155148.1 carboxylesterase clade B, member 2 precursor [Nasonia vitripennis]](https://blast.ncbi.nlm.nih.gov/Blast.cgi#alnHdr_Query_42990) | 86% | 1,00E-77 | 32.73% |
| 1652 | Carboxylesterase clade E, member 11 precursor | [NP_001155148.1 carboxylesterase clade B, member 2 precursor [Nasonia vitripennis]](https://blast.ncbi.nlm.nih.gov/Blast.cgi#alnHdr_Query_42990) | 91% | 4,00E-80 | 32.78% |
| 1730 | Trypsin-like | [NP_001155175.1 serine protease precursor [Nasonia vitripennis]](https://blast.ncbi.nlm.nih.gov/Blast.cgi#alnHdr_Query_43015) | 86% | 1,00E-39 | 33.57% |
| 1794 | Trypsin-like | [NP_001155175.1 serine protease precursor [Nasonia vitripennis]](https://blast.ncbi.nlm.nih.gov/Blast.cgi#alnHdr_Query_43015) | 88% | 9,00E-34 | 26.58% |
| 1835 | Serine protease homolog 21 precursor | [NP_001155060.1 serine protease homolog 21 precursor [Nasonia vitripennis]](https://blast.ncbi.nlm.nih.gov/Blast.cgi#alnHdr_Query_43023) | 99% | 0.0 | 70.83% |
| 1855 | Trypsin-like | [NP_001155175.1 serine protease precursor [Nasonia vitripennis]](https://blast.ncbi.nlm.nih.gov/Blast.cgi#alnHdr_Query_43015) | 86% | 2,00E-34 | 32.04% |
| 1907 | Serine protease 22 precursor | [NP_001155043.1 serine protease 22 precursor [Nasonia vitripennis]](https://blast.ncbi.nlm.nih.gov/Blast.cgi#alnHdr_Query_43025) | 99% | 0.0 | 69.07% |
| 1971 | Chymotrypsin-1-like | [NP_001155042.1 serine protease 97 precursor [Nasonia vitripennis]](https://blast.ncbi.nlm.nih.gov/Blast.cgi#alnHdr_Query_43022) | 86% | 6,00E-23 | 28.97% |
| 2095 | Carboxylesterase clade B, member 2 precursor | [NP_001155148.1 carboxylesterase clade B, member 2 precursor [Nasonia vitripennis]](https://blast.ncbi.nlm.nih.gov/Blast.cgi#alnHdr_Query_42990) | 97% | 0.0 | 68.59% |
| 2283 | Chymotrypsin-1-like | [NP_001155175.1 serine protease precursor [Nasonia vitripennis]](https://blast.ncbi.nlm.nih.gov/Blast.cgi#alnHdr_Query_43015) | 85% | 5,00E-43 | 35.69% |
| 2399 | Trypsin-like | [NP_001155175.1 serine protease precursor [Nasonia vitripennis]](https://blast.ncbi.nlm.nih.gov/Blast.cgi#alnHdr_Query_43015) | 91% | 4,00E-23 | 29.81% |
| 2622 | Low-density lipoprotein receptor-related protein 6-like | [NP_001155040.1 low-density lipoprotein receptor-like venom protein precursor [Nasonia vitripennis]](https://blast.ncbi.nlm.nih.gov/Blast.cgi#alnHdr_Query_15917) | 8% | 3.6 | 31.03% |
| 2872 | Venom carboxylesterase-6-like | [NP_001155148.1 carboxylesterase clade B, member 2 precursor [Nasonia vitripennis]](https://blast.ncbi.nlm.nih.gov/Blast.cgi#alnHdr_Query_42990) | 90% | 6,00E-71 | 31.41% |
| 2910 | Venom protein R precursor | [NP_001155164.1 venom protein R precursor [Nasonia vitripennis]](https://blast.ncbi.nlm.nih.gov/Blast.cgi#alnHdr_Query_43042) | 97% | 6,00E-66 | 75.74% |
| 3239 | Carboxylesterase clade E, member 11 precursor | [NP_001155148.1 carboxylesterase clade B, member 2 precursor [Nasonia vitripennis]](https://blast.ncbi.nlm.nih.gov/Blast.cgi#alnHdr_Query_42990) | 86% | 2,00E-73 | 31.24% |
| 3708 | Trypsin beta-like | [NP_001155175.1 serine protease precursor [Nasonia vitripennis]](https://blast.ncbi.nlm.nih.gov/Blast.cgi#alnHdr_Query_43015) | 81% | 9,00E-24 | 27.95% |
| 3815 | Inosine-uridine preferring nucleoside hydrolase-like precursor | [NP_001155172.1 inosine-uridine preferring nucleoside hydrolase-like precursor [Nasonia vitripennis]](https://blast.ncbi.nlm.nih.gov/Blast.cgi#alnHdr_Query_43055) | 100% | 2,00E-103 | 47.08% |
| 3878 | Venom acid phosphatase Acph-1 | [NP_001155147.1 venom acid phosphatase-like precursor [Nasonia vitripennis]](https://blast.ncbi.nlm.nih.gov/Blast.cgi#alnHdr_Query_42989) | 96% | 1,00E-96 | 41.71% |
| 4479 | Endonuclease-like venom protein precursor | [NP_001155087.1 endonuclease-like venom protein precursor [Nasonia vitripennis]](https://blast.ncbi.nlm.nih.gov/Blast.cgi#alnHdr_Query_42999) | 74% | 7,00E-60 | 32.88% |
| 6082 | Endonuclease-like venom protein precursor | [NP_001155087.1 endonuclease-like venom protein precursor [Nasonia vitripennis]](https://blast.ncbi.nlm.nih.gov/Blast.cgi#alnHdr_Query_42999) | 78% | 2,00E-79 | 35.32% |
| 6303 | Venom dipeptidyl peptidase 4 isoform X2 | [XP_001599462.2 PREDICTED: venom dipeptidyl peptidase 4 isoform 1 [Nasonia vitripennis]](https://blast.ncbi.nlm.nih.gov/Blast.cgi#alnHdr_Query_42998) | 97% | 0.0 | 72.98% |
| 8693 | General odorant-binding protein 56d | [NP_001155150.1 GOBP-like venom protein precursor [Nasonia vitripennis]](https://blast.ncbi.nlm.nih.gov/Blast.cgi#alnHdr_Query_43004) | 44% | 0.002 | 26.56% |
| 9106 | General odorant-binding protein 71 | [NP_001155150.1 GOBP-like venom protein precursor [Nasonia vitripennis]](https://blast.ncbi.nlm.nih.gov/Blast.cgi#alnHdr_Query_43004) | 82% | 0.029 | 27.59% |
| 9844 | Liver carboxylesterase 1 | [NP_001155148.1 carboxylesterase clade B, member 2 precursor [Nasonia vitripennis]](https://blast.ncbi.nlm.nih.gov/Blast.cgi#alnHdr_Query_42990) | 81% | 1,00E-43 | 26.40% |
| 11004 | Serine protease 67 precursor | [NP_001155043.1 serine protease 22 precursor [Nasonia vitripennis]](https://blast.ncbi.nlm.nih.gov/Blast.cgi#alnHdr_Query_43025) | 83% | 8,00E-43 | 30.16% |

**Supplementary Table 2: *Torymus sinensis* venom proteins identified through the combined proteomic and transcriptomic approach aligned with *Nasonia vitripennis* proteins in de Graaf et al.^30^ (Table S2. Venom proteins discovered by a proteomic approach).** In the table are reported: the contig number, the protein name, the *N. vitripennis* protein and data concerning the alignment of the two proteins (query cover, E-value and identity). The alignments were performed with the software BLASTp (<https://blast.ncbi.nlm.nih.gov/Blast.cgi?PAGE=Proteins>).

| **Contig** | ***Torymus sinensis* protein** | ***Nasonia vitripennis* protein^30^** | **Query cover** | **E-value** | **Identity** |
| --- | --- | --- | --- | --- | --- |
| 2 | Trypsin-3-like | [NP_001166092.1 serine protease 87 precursor [Nasonia vitripennis]](https://blast.ncbi.nlm.nih.gov/Blast.cgi#alnHdr_Query_52027) | 52% | 6,00E-14 | 38.61% |
| 128 | Pancreatic triacylglycerol lipase | [XP_016845666.1 pancreatic lipase-related protein 2-like isoform X1 [Nasonia vitripennis]](https://blast.ncbi.nlm.nih.gov/Blast.cgi#alnHdr_Query_43057) | 78% | 2,00E-56 | 32.89% |
| 495 | Pancreatic triacylglycerol lipase | [XP_016845666.1 pancreatic lipase-related protein 2-like isoform X1 [Nasonia vitripennis]](https://blast.ncbi.nlm.nih.gov/Blast.cgi#alnHdr_Query_43057) | 70% | 4,00E-66 | 36.62% |
| 1105 | Serine protease inhibitor 3/4 isoform X16 | [XP_001606111.2 ovalbumin-related protein X isoform X15 [Nasonia vitripennis]](https://blast.ncbi.nlm.nih.gov/Blast.cgi#alnHdr_Query_52028) | 98% | 0.0 | 71.83% |
| 1143 | Trypsin-like | [NP_001166092.1 serine protease 87 precursor [Nasonia vitripennis]](https://blast.ncbi.nlm.nih.gov/Blast.cgi#alnHdr_Query_43061) | 81% | 6,00E-35 | 32.54% |
| 2025 | Serpin 5 precursor | [XP_001606111.2 ovalbumin-related protein X isoform X15 [Nasonia vitripennis]](https://blast.ncbi.nlm.nih.gov/Blast.cgi#alnHdr_Query_43062) | 93% | 2,00E-44 | 27.01% |
| 3591 | Venom acid phosphatase Acph-1-like | [NP_001155146.1 venom acid phosphatase-like precursor [Nasonia vitripennis]](https://blast.ncbi.nlm.nih.gov/Blast.cgi#alnHdr_Query_43051) | 99% | 4,00E-125 | 47.53% |
| 7646 | Aminopeptidase N isoform X2 | [XP_031781511.1 aminopeptidase N isoform X1 [Nasonia vitripennis]](https://blast.ncbi.nlm.nih.gov/Blast.cgi#alnHdr_Query_16785) | 95% | 4,00E-62 | 23.05% |
| 8199 | Venom metalloproteinase 2-like isoform X2 | [XP_008213426.2 venom metalloproteinase 3-like [Nasonia vitripennis]](https://blast.ncbi.nlm.nih.gov/Blast.cgi#alnHdr_Query_43059) | 75% | 5,00E-24 | 22.80% |
| 11185 | Major royal jelly protein-like 7 precursor | [NP_001154978.1 major royal jelly protein-like 9 precursor [Nasonia vitripennis]](https://blast.ncbi.nlm.nih.gov/Blast.cgi#alnHdr_Query_43060) | 90% | 9,00E-78 | 34.99% |

**Supplementary Table 3: *Torymus sinensis* venom proteins identified through the combined proteomic and transcriptomic approach aligned with *Nasonia vitripennis* proteins in de Graaf et al.^30^ (Table S3. Non-secretory proteins discovered by a proteomic approach)**. In the table are reported: the contig number, the protein name, the *N. vitripennis* protein and data concerning the alignment of the two proteins (query cover, E-value and identity). The alignments were performed with the software BLASTp (<https://blast.ncbi.nlm.nih.gov/Blast.cgi?PAGE=Proteins>).

| **Contig** | **Peptides** | **Signal Peptide Percentage** | **Protein Name** | **Correspondent Protein in NCBI Database** |
| --- | --- | --- | --- | --- |
| 1 | \| 1. CADVNIIPNMVCQNVYR \| \| --- \| \| 1. EDLAPAMYTR \| \| 1. EVGTIYIPR \| \| 1. EVLDSHLCGHMVQR \| \| 1. NVQYTVVAGVTHAHNKEQPNR \| \| 1. SGSDYFERR \| \| 1. SPTHPEGLCTGDSGSPLVIGNTIIGVVSSSPTGCR \| \| 1. VASFIPFIINAMHGVR \| \| 1. YIITVAHCFINLETNR \| | 95.07 | Trypsin-like | XP_016836963.1_trypsin-like [Nasonia vitripennis] |
| 4 | \| 1. EKYDEEDVLINK \| \| --- \| \| 1. GEKIPVEK \| \| 1. LKTPIKYSK \| \| 1. NMFCAYER \| \| 1. NYDDDTTENDIALLK \| \| 1. RTDSCQGDSGGPAVINGK \| \| 1. VGQRLPGSK \| \| 1. VSNQTDCREKYDEEDVLINK \| \| 1. YDEEDVLINK \| | 99.50 | Serine protease 33 isoform X2 | XP_031789341.1_serine protease 33 isoform X2 [Nasonia vitripennis] |
| 12 | \| 1. AINCLDIK \| \| --- \| \| 1. ASLDAQNITCDNYQDNCVR \| \| 1. MISEFSCPIR \| \| 1. VALLCQQLTDEEIKR \| | 99.93 | NA | NA |
| 18 | \| 1. DHVINLISK \| \| --- \| \| 1. FTIDMDR \| \| 1. GQEVTMYAVLPSTPGTAALK \| \| 1. GTEAAAATSVILDR \| \| 1. LPTKPSGTPFDAVLPAR \| \| 1. LQSFDPSAPACK \| \| 1. NTAGISNPGLYADDVIHK \| \| 1. SEVISVDFSGHSR \| \| 1. SQLTADIIDGLVGK \| \| 1. STMGLAQAPAR \| \| 1. TNASSTPAASAPLPTAPEASK \| \| 1. WRDHVINLISK \| | 99.61 | Serine protease inhibitor 28Dc isoform X2 | XP_001603946.3 - Serine protease inhibitor 28Dc isoform X2 [Nasonia vitripennis] |
| 25 | \| 1. DSQLCGHVTQR \| \| --- \| \| 1. EDVEPATYTR \| \| 1. FAEVNIISNNACR \| \| 1. HFVPDIR \| \| 1. NTIIGVLSDGPPGCREDVEPATYTR \| \| 1. NTPEGICEGDSGAPLVIR \| \| 1. RFAEVNIISNNACR \| | 97.34 | Trypsin-like | XP_016836963.1_trypsin-like [Nasonia vitripennis] |
| 69 | \| 1. ENVLPAIYTR \| \| --- \| \| 1. HLCGGGILSNR \| \| 1. IISNEACR \| \| 1. LAQYVVGRDHIK \| \| 1. VTSFIPFITNAVNGVR \| \| 1. YVEVNTGSYSYEKR \| | 96.99 | Trypsin-3-like | XP_016836949.1_trypsin-3-like [Nasonia vitripennis] |
| 70 | \| 1. DIVDSHLCGR \| \| --- \| \| 1. EDLAPAVYTR \| \| 1. EFNIIMVPNAPR \| \| 1. FAQVDILSNNECR \| \| 1. FRDIVDSHLCGR \| \| 1. LTTADIAIVELASDLGLSR \| \| 1. NAEYIVVAGVHQAHNRQER \| \| 1. NAMTNVHAADMR \| \| 1. SPATPEGLCGGDSGAPLVMGSTIIGVVSSSPHGCREDLAPAVYTR \| \| 1. YIMTVAHAFVHPETNIFR \| | 92.15 | Trypsin-like | XP_016836963.1_trypsin-like [Nasonia vitripennis] |
| 87 | \| 1. EDVAPTIYTR \| \| --- \| \| 1. FDNAEYIIVAGATQAHNIHELHR \| \| 1. GGNVAVVHQHTYAVSIQR \| \| 1. VSDIAVVK \| \| 1. VTSFVPFITR \| \| 1. VVAQIYIPR \| | 98.87 | Trypsin-like | XP_016836963.1_trypsin-like [Nasonia vitripennis] |
| 106 | \| 1. ALQLLITK \| \| --- \| \| 1. DQNLALK \| \| 1. FLTECAVDKYR \| \| 1. FSFESDSHLGYSNSNNRK \| \| 1. GPHMMGFTSSEAR \| \| 1. GQLLTTVR \| \| 1. KFLTECAVDKYR \| \| 1. LEFQEISNLK \| \| 1. LLPFRPTIEMK \| \| 1. LSDTFFVTGIDYK \| \| 1. MMNHCYENNQ \| \| 1. PIDPFVVFDELSGIVTAR \| \| 1. QSISMSGSPLCLYWGMQSR \| \| 1. SVDELLHVLYTASTTDIISK \| \| 1. SWTNVLDATVER \| \| 1. VSGVGHAEDLQYLFYIPFVGMSFNNVDVQTTVNK \| | 95.89 | Carboxylesterase clade B, member 2 precursor | NP_001155148.1_carboxylesterase clade B, member 2 precursor [Nasonia vitripennis] |
| 125 | \| 1. ASPEGVCTR \| \| --- \| \| 1. DSGAPLVIGNTLIGVLSAGPK \| \| 1. EYDPIIVFPFVR \| \| 1. LAEYVPGREHAR \| \| 1. NLSGNIMDSHLCGHMVQR \| \| 1. TNQHVEVCSGSHCYER \| \| 1. VLAEIM \| | 94.91 | Chymotrypsin-1-like | XP_008214285.1chymotrypsin-1-like [Nasonia vitripennis] |
| 128 | \| 1. ETQPFCVLDKDVK \| \| --- \| \| 1. IVGLQIAR \| \| 1. KPIDVANIPNEK \| \| 1. KQPEYVTVR \| \| 1. LFANVIEK \| \| 1. NNVALNCFGMPIK \| \| 1. SIIQESVCSHGR \| | 92.20 | Pancreatic triacylglycerol lipase | XP_031783896.1_pancreatic triacylglycerol lipase [Nasonia vitripennis] |
| 157 | \| 1. ALEAEIDK \| \| --- \| \| 1. EIIQPSQECLK \| \| 1. FDNFVASISDK \| \| 1. ICLTEPIK \| \| 1. IFETEDVK \| \| 1. KEIIQPSQECLK \| \| 1. KLTAECVEK \| \| 1. LTAECVEK \| \| 1. MSLDDLASKDLQDK \| \| 1. NKLSEIEGSSIAIVK \| \| 1. SKIFETEDVK \| \| 1. STAESLLR \| | 99.94 | Venom protein u precursor | NP_001155170.1_venom protein U precursor [Nasonia vitripennis] |
| 158 | \| 1. FTGECNNAEAQIR \| \| --- \| \| 1. VLMSQAIGPYCDVK \| \| 1. LTVDGCQK \| \| 1. NHFFAVANTK \| \| 1. SYLITYDELDPYSK \| \| 1. YDLFLAK \| | 96.03 | Uncharacterized protein LOC100115344 | XP_001600095.2_uncharacterized protein LOC100115344 [Nasonia vitripennis] |
| 181 | 1. AFLGGQEPVVVGFFEK 2. DDSPLSAAFHTVSK 3. GSDPPITLAK 4. IFNKDEMVSDYNGPR 5. IGFGHTSDEEILKSEDLK 6. SEDLKNHIVIYRPK 7. VDCTEAGKDTCNK 8. VLQNKFEPSK 9. YSVSGYPTLK | 99.68 | Protein disulfide-isomerase A3 | XP_001599732.2_protein disulfide-isomerase A3 [Nasonia vitripennis] |
| 235 | \| 1. DFRDSQLCGHIK \| \| --- \| \| 1. DSQLCGHIK \| \| 1. LATYEVGR \| \| 1. NTIIGVASDSPK \| \| 1. RFAEVNILSNMACR \| \| 1. SPFHPEGLCTGDSGSPLVIR \| \| 1. VTSFLPFITQAMNGVR \| \| 1. YADIAVVELR \| \| 1. DVEVCSGSYCYER \| | 84.68 | Trypsin-like | XP_016836963.1_ PREDICTED: trypsin-like [Nasonia vitripennis] |
| 266 | 1. AIEELGGLETESDYPYDAQDEK 2. IQVVSGLLYK 3. QMLGAPTK 4. SMDEAGISDFK 5. SPYVIYR 6. WGEQGYYR 7. YGVTQFADLTK | 98.37 | Uncharacterized protein LOC100123649 | XP_001607324.1_uncharacterized protein LOC100123649 [Nasonia vitripennis] |
| 270 | \| 1. AIVVGYGHNYITMR \| \| --- \| \| 1. EFNPIGDK \| \| 1. HDPHTNTAVEVCR \| \| 1. HGCGGAILNSR \| \| 1. LAEYEVGRDHTK \| \| 1. NSVWTVVAGVR \| \| 1. PFFGLIEDSHLCGHVR \| \| 1. QNTYAVSIQKDGR \| \| 1. RFAQVNILSNNACR \| \| 1. SPAEPEGLCTGDSGGPLVMGSSNTLIGLVSR \| \| 1. VVLPAIAGPHPTYANER \| \| 1. GSYCYER \| \| 1. ENLEPAIYTR \| | 89.99 | Trypsin-like | XP_016836963.1­­_trypsin-like [Nasonia vitripennis] |
| 281 | \| 1. GSLHTIVK \| \| --- \| \| 1. INISDLPEEKRK \| \| 1. KHVDTESSVPR \| \| 1. LACALACIYEDEKPHR \| \| 1. RKELLDIANNCK \| \| 1. SELLTPEVEEK \| \| 1. TQAGEDLCK \| \| 1. TRINISDLPEEK \| \| 1. YFDEMVKPK \| | 56.35 | putative odorant binding protein 47 | ­ CCD17816.1_putative odorant binding protein 47 [Nasonia vitripennis] |
| 289 | \| 1. AINIGDVVVDPK \| \| --- \| \| 1. AIQTTRPDHPCSANHGCQK \| \| 1. AVCGCPYGEK \| \| 1. CACSGGYQLGK \| \| 1. DLVVHPNK \| \| 1. FCFALPSSSK \| \| 1. GQTLATNGKPTFTVINPSLFEIGKDSR \| \| 1. GVSPHNVIR \| \| 1. GYLFIAQAYEK \| \| 1. HSNLDGQDIQEVPTPK \| \| 1. IDLTGNNHK \| \| 1. IDYDAEDGLIFYTSSYPDNK \| \| 1. IESMDYDGNDRK \| \| 1. KIHFEDSNAPEK \| \| 1. LDQVSIDALALDYENDR \| \| 1. LNTDGSNLTGIIK \| \| 1. LNTNEQTIMKPDILSGDVGSITYDWVTK \| \| 1. LYWIESK \| \| 1. NIVTLRDK \| \| 1. PTFTVINPSLFEIGK \| \| 1. QADPENTFKK \| \| 1. QAGQPIILAETSPSPYITDVK \| \| 1. QPTEQILK \| \| 1. RIESMDYDGNDRK \| \| 1. RVYFVDTVSR \| \| 1. SCTSVPEILFYPQENFLR \| \| 1. SLIWTVDSNGQNR \| \| 1. TSLDKHLVVAEK \| \| 1. VERPFLPCNFTSFK \| \| 1. VILENVEPIALK \| \| 1. VIYSELGDMNEVLDTLFSPVMR \| \| 1. VVIDFVR \| \| 1. VYFVDTVSR \| \| 1. VYYTESFTETVR \| | 80.50 | Low-density lipoprotein receptor-related protein 2-like | XP_031778155.1_low-density lipoprotein receptor-related protein 2-like [Nasonia vitripennis] |
| 293 | \| 1. FVAPAHVELAK \| \| --- \| \| 1. GYNSDIEEKFR \| \| 1. KGYNSDIEEK \| \| 1. NSGAEDVGFVDIPEGDEEKLK \| \| 1. TEGAVTPVK \| \| 1. TKPGALLQGYEKPVGAK \| \| 1. TYPYEAQDDTCR \| \| 1. YGNNGCNGGLMDNAFK \| | 98.29 | Cathepsin L | XP_031785563.1_cathepsin L [Nasonia vitripennis] |
| 368 | \| 1. FENAEYIVVAGVTNAHNR \| \| --- \| \| 1. IYVPQEYNPLIVTNVK \| \| 1. LATYEVVPCGR \| \| 1. LVEFCSGSYCNER \| \| 1. RFALVMIR \| | 97.92 | Trypsin-like | XP_016836963.1_trypsin-like [Nasonia vitripennis] |
| 378 | \| 1. AVVCGYGHNR \| \| --- \| \| 1. DIVDSHLCGHMR \| \| 1. ENLVPAVYTR \| \| 1. EYNPIMVPNVQR \| \| 1. IVEVCSQSYCYQR \| | 95.65 | Chymotrypsin-1-like | XP_008214285.1_chymotrypsin-1-like [Nasonia vitripennis] |
| 392 | \| 1. DVCNSGLGK \| \| --- \| \| 1. FLTTVVNPK \| \| 1. KFSYGLVENVAK \| \| 1. NLQNVVAYEK \| \| 1. QAVFYSADDAFTSEKDAAQLKK \| \| 1. YGNVIPPEYDFSK \| | 97.30 | Lipase 3-like | XP_008216710.1_lipase 3-like [Nasonia vitripennis] |
| 428 | \| 1. CHITNIACLLHNK \| \| --- \| \| 1. FDGILGMAFDK \| \| 1. FTVVFDTGSSNLWVPSKK \| \| 1. IGGEMILGGSDPDHYEGEFTYVPVSR \| \| 1. ISVDGVPPVFYNMVK \| \| 1. KAYWQFQMDK \| \| 1. LQEVGTELQQLK \| \| 1. QGLVPQPIFSFYLNR \| \| 1. SFSLKGEDYVLK \| \| 1. VQDTTFAEALSEPGLAFVAAK \| \| 1. YGSGSLSGFLSTDVVTIANAK \| \| 1. YYTEFDMANDR \| | 99.46 | Lysosomal aspartic protease-like | XP_001600543.1_lysosomal aspartic protease-like [Nasonia vitripennis] |
| 445 | 1. ADVFGANK 2. AIDLGVAGFR 3. GWDSLSSLQSWGFQDR 4. HMWPGDLEIIYSGLK 5. KTMVHLFEWK 6. NCALSGLHDLNQGR 7. RPYIYQEVIDLGGGEGVKK 8. SGNEQDFADMVR 9. TQYNGLGDVIEFVFGMLLGR 10. WQDVALECEK | 95.97 | Alpha-amylase-like | XP_031783266.1_alpha-amylase-like [Nasonia vitripennis] |
| 495 | \| 1. FTDQVLEK \| \| --- \| \| 1. GSNTWNYLAAAVSTK \| \| 1. HILYLGLGLPER \| \| 1. ITGLDPAQPCFTTADK \| \| 1. IVGQQIAK \| \| 1. KMLETSICSHGR \| \| 1. LDRHDALYVDVIHTNAR \| \| 1. NGGIFFVATSDDNPFCGIK \| \| 1. QSCSEDVCPEMGISSINSYPR \| \| 1. QVAFDANFK \| \| 1. TSEQIDTLLK \| \| 1. VFANAIEK \| | 85.31 | Pancreatic triacylglycerol lipase | XP_031778248.1_pancreatic triacylglycerol lipase [Nasonia vitripennis] |
| 529 | \| 1. APSTQVSSWIR \| \| --- \| \| 1. CDTYGYDEDVTYTK \| \| 1. DIYNNIQPK \| \| 1. EDYDYHVIEWNVNR \| \| 1. GETYEYIPR \| \| 1. GSGGFLSLK \| \| 1. IGTDMWICPSDKDSR \| \| 1. ILNELPDQIMLK \| \| 1. KAAINLDDLDYADTK \| \| 1. KCNGELWNCEYK \| \| 1. KLDSLTLNMK \| \| 1. KVNHIIHIQIQEGK \| \| 1. LAPGMEQR \| \| 1. LFTYNYEPHLKVESN \| \| 1. LLPGGVIGQNTLR \| \| 1. LSNTLNAVK \| \| 1. NMKDEIVDK \| \| 1. NNLYEIYLR \| \| 1. RYDYIEYGNSQR \| \| 1. STQTPGQMCK \| \| 1. SVTADVYDNKVVTGVR \| \| 1. SVWYSTYKEPSGR \| \| 1. TCPDYQR \| \| 1. TIHWMLIETDVNVGDSILTILSR \| \| 1. TKVYDCYDK \| \| 1. VANGICSVVK \| \| 1. WKPVEPYKVNEPMPGTK \| \| 1. YKDDPVLFR \| \| 1. YMINEADINQEGTCTR \| | 99.74 | Hypothetical protein LOC100679659 isoform X1 | XP_003424464.1_ hypothetical protein LOC100679659 isoform X1 [Nasonia vitripennis] |
| 557 | \| 1. FAQVNILTR \| \| --- \| \| 1. IYIPQEYNPIMK \| \| 1. KFAQVNILTR \| \| 1. MACQNFFR \| \| 1. NIMDSQLCGNLIQR \| \| 1. VTSFLPFIINAMNGVR \| \| 1. VVEVCSGSDCYER \| \| 1. YIITAAHCFINPETNR \| | 96.18 | Trypsin-like | XP_016836963.1_trypsin-like [Nasonia vitripennis] |
| 575 | \| 1. FANSLKEETKK \| \| --- \| \| 1. IEELKPQYSK \| \| 1. ILNDWDETCDGCIEK \| \| 1. KIEAEFKK \| \| 1. KMDAQICDLR \| \| 1. PLSWSLPADK \| \| 1. QIDLSTVDLKK \| | 99.84 | Mesencephalic astrocyte-derived neurotrophic factor homolog | XP_001604026.2_mesencephalic astrocyte-derived neurotrophic factor homolog [Nasonia vitripennis] |
| 590 | \| 1. CIIEAKEDECK \| \| --- \| \| 1. HVIDTLNDAQK \| \| 1. MGCLTMCYLK \| \| 1. NELEHDIPMKK \| | 99.94 | Odorant binding protein 53 | CCD17822.1_putative odorant binding protein 53 [Nasonia vitripennis] |
| 706 | \| 1. EDLEPSIYTR \| \| --- \| \| 1. EFNMVPSVDR \| \| 1. FAQVTILSNMACR \| \| 1. HDPNTNQIVEVCSSSDCYER \| \| 1. LAEYEVISCGR \| \| 1. NFATDIMDIHLCGHIR \| \| 1. YIITAAHCFIELGTNK \| | 96.07 | Trypsin-like | XP_016836963.1_trypsin-like [Nasonia vitripennis] |
| 753 | \| 1. EDLRPALYTR \| \| --- \| \| 1. ENLGLNQFR \| \| 1. TADIAVLELR \| \| 1. VNIISNVACR \| \| 1. VVEVCSGSYCYK \| \| 1. DSHLCGHMVQR \| | 96.54 | Trypsin-like | XP_016836963.1_trypsin-like [Nasonia vitripennis] |
| 775 | \| 1. GGTASDVGQHTYAVSIQVR \| \| --- \| \| 1. GKHVAGGGILNNR \| \| 1. HIVTVAHLLTDPSTNR \| \| 1. QEQYRVER \| \| 1. LHAESIR \| \| 1. EVAKIYIPR \| | 89.21 | Trypsin-like | XP_016836963.1_trypsin-like [Nasonia vitripennis] |
| 784 | \| 1. ADIDVFNSKNDKITVNAK \| \| --- \| \| 1. AFKDLLKPIMNELR \| \| 1. AGGEQLFEAK \| \| 1. AGVESLGGNK \| \| 1. AHFGQIRPR \| \| 1. ALPVYEMAQNAYR \| \| 1. ALQSIHKEGSEAVKPEEAR \| \| 1. ALYNDKNPK \| \| 1. ASADPFKQNAK \| \| 1. ASAEAIVKR \| \| 1. ATYPQLK \| \| 1. AVVGLVSQPCEK \| \| 1. CTVGCHGMHSDQSYK \| \| 1. DFFSTVYNYILK \| \| 1. DGDTQVIHKVR \| \| 1. DGFTNMNGLSNYIR \| \| 1. DRTQYTYHLYSNKR \| \| 1. DTQKVETEFALLGHKPVVGNVEIK \| \| 1. EGQTYLYALDGTSVTSVSEGQGEATLK \| \| 1. EGSEAVKPEEARR \| \| 1. ELAIVAFHDIPIVK \| \| 1. ELPTVSDLYYTYRPLYR \| \| 1. ESAAVLSLPSR \| \| 1. EVKEFVQLLIQQVR \| \| 1. EVVTEFGDLFEHLKK \| \| 1. FAFVQGADLK \| \| 1. FALSTDNKLTYPK \| \| 1. FEFDETYLDQQDDNGMVEYTK \| \| 1. FGAELSGNINK \| \| 1. FMKPDFAYNNK \| \| 1. FTYPSQPK \| \| 1. FVEHIEELRQQLNDICGK \| \| 1. FVLGQLAEIHADAFK \| \| 1. FYVTVENNDVSSYHFK \| \| 1. GFVTVDGR \| \| 1. GIDVSLGVPK \| \| 1. GILGDANNEPSDDYILPSGK \| \| 1. GKKEDSPTAAVSLPK \| \| 1. GNEVDEDLDLDLSIK \| \| 1. GNGNINIDIPK \| \| 1. GNNVDKPGIMKGEIEAK \| \| 1. GQNPNGNLVLNLK \| \| 1. HDEDKKVTLEGNGSLK \| \| 1. HEIDVLGGCVTDFTFFK \| \| 1. HVANFELAYDAGKDPNKK \| \| 1. HYTSNNNANIEGGDINHMSFTHK \| \| 1. IAASATDLGNAYK \| \| 1. IAFDSPIKK \| \| 1. IATQIKDVLDKEK \| \| 1. IDVSLYLDRK \| \| 1. IEDSIPPAKK \| \| 1. IFAHFDQGINK \| \| 1. IFITVTSEGK \| \| 1. IGYLGIGQIIGR \| \| 1. IKGSGDVSVAGSK \| \| 1. IKLDTGLECTPDKLNNHIEIISGGVK \| \| 1. ISTDSDVTK \| \| 1. IYLSFVACPCNK \| \| 1. IYQVGSFITSHLR \| \| 1. KASAEAIVK \| \| 1. KDDKLDVQAHTEIADYVTK \| \| 1. KDILTVYHR \| \| 1. KFNVPVEFGLLSSTK \| \| 1. KFVEHIEELR \| \| 1. KGDQTHQVQGTVEVADHDGGKK \| \| 1. KIYSQAINAFR \| \| 1. KKPLEANTDNK \| \| 1. KLGENEYSGDYK \| \| 1. KNDVNVGVDADVNLNNKK \| \| 1. KPFYPLSGPFK \| \| 1. KQADIIFVVEQEADNAK \| \| 1. KQEIVSISSDVLYNNGKPDK \| \| 1. KSVETEAELKYDK \| \| 1. KTDIAVTIDGVK \| \| 1. KVHAEIANKPSSQDGR \| \| 1. KVIVDANANVVSMKDR \| \| 1. KVVGIDGNAVSTPK \| \| 1. KYVFNDVQFVSNGR \| \| 1. LANIAADK \| \| 1. LANIAADKK \| \| 1. LATTYVEAILK \| \| 1. LKADVELAVKPDCIR \| \| 1. LKNELHADQTIKEIQEILNK \| \| 1. LKNVQVNGAAVPQQDLEK \| \| 1. LLTYNDDQK \| \| 1. LNPSCAAAAAK \| \| 1. LPGLSTIR \| \| 1. LQLTQHAEYNLEPEKFALSTDNK \| \| 1. LQSTLDTDHLTHLNHNLR \| \| 1. LRPFSNGDAGAK \| \| 1. LSDKEVHVFNSYCEASKDCAHFK \| \| 1. LTDGYAAAIR \| \| 1. LVCTVETSISCYLR \| \| 1. LVTTLHTATGYDLNVK \| \| 1. NDQVEYFAR \| \| 1. NFEDLTKYER \| \| 1. NFLPTQELK \| \| 1. NIDLEIDHKRENEK \| \| 1. NIIEILTYK \| \| 1. NILAKPFADQEEDSEIRNK \| \| 1. NIMAVMNQYR \| \| 1. NLEAYVTVPIYTVK \| \| 1. NLNQCSNRENIK \| \| 1. NLVTGSTNYKR \| \| 1. NLYSQLGLK \| \| 1. NPANAFGIEWEYHR \| \| 1. PTNQNQYIK \| \| 1. QAELDTFAK \| \| 1. QGLFSGIVNPTAGIQSTPIIGAHQK \| \| 1. QQGITDVFIGLIGFGEDLKWPR \| \| 1. QRLDVELGSFK \| \| 1. QVGEEIGK \| \| 1. RAVISLFQSSVVQEEGSTSR \| \| 1. RIAEGLVNTEIEEDCVCGAEAGYGHTAK \| \| 1. RLGENQYSWSASALPSK \| \| 1. RSEEDTIIAALK \| \| 1. SACDQMVAQGTVK \| \| 1. SALSSCFNYVEPQAFR \| \| 1. SAQEQTLTVTVK \| \| 1. SDQHFALTTNSIDLGSSLSYTDKHQKPK \| \| 1. SEPTITLQEAKEDK \| \| 1. SLEILLETPNSR \| \| 1. SLIFEAPHPVVK \| \| 1. SLIHLLQTQITVENIFGFFETQFPIDVSYLK \| \| \| 1. SLVGYDKDSIYTFADSKK \| \| 1. SNGNVLVNNKPADLPASTK \| \| 1. SPLSPFSLQDYR \| \| 1. SSVESIEK \| \| \| \| 1. SYLTANGQFAYQNGK \| \| 1. TATGEAAEVVVELIK \| \| 1. TFGGLIAAITETAKK \| \| 1. TFTGTLNVLDKPTLTVSDNYNHDLTGEIKK \| \| \| \| 1. TIIDDTK \| \| 1. TKFPEDIRK \| \| 1. TNTKIDIPAILADPQNTAFK \| \| 1. TSLIFTGDVNVDKNAVGIK \| \| 1. TVVETILTSK \| \| 1. VAAIEALPTK \| \| 1. VDDTNEVKK \| \| 1. VGVTIFDSATK \| \| 1. VHAEIANKPSSQDGRR \| \| 1. VIEHFFGPK \| \| 1. VLLDALYR \| \| 1. VPDYVIAPIEEFCHNIK \| \| 1. VSGFYHGK \| \| 1. YADYSLDHQANLYLHSSKDR \| \| 1. YCQQHSCENVPEIK \| \| 1. YGDKTGTGDLTLQWHPELTK \| \| 1. YGYFADGGYFVTFDGK \| \| 1. YIHTDGKDLAYNIVIK \| \| 1. YKPVLEYKVPEHIEK \| \| 1. YLDDATLQK \| \| 1. YTQMTEIELEEAPGIHLVFTCPAGK \| \| 1. YVSLNLQAAVEPDPK \| \| 1. YYHVSPLNDLEVSGK \| | 95.37 | Apolipophorins | XP_001604024.1_apolipophorins [Nasonia vitripennis] |
| 789 | \| 1. ENNKFQNYEYSVVAGVTNAHNIHQQYR \| \| --- \| \| 1. FITYVALPANAR \| \| 1. IYIPLEFIPVGNK \| \| 1. KFALVNILSNMVCK \| \| 1. KIYIPLEFIPVGNK \| \| 1. LAEYEEIVCGPR \| \| 1. LTSDLGLNR \| \| 1. PFPTYENHQAVVCGYGHNLNTMQHNTPTNQNYER \| \| 1. VDSKPDIYTR \| \| 1. VTTFMPFIIQAINSVR \| \| 1. YIITAAHCFINR \| \| 1. VADIAVLK \| | 88.13 | Trypsin-like | XP_016836963.1_ trypsin-like [Nasonia vitripennis] |
| 874 | \| 1. CFQSYYPNYYTER \| \| --- \| \| 1. DCDGIWQESK \| \| 1. IIDAAYSTDEK \| \| 1. KLTGNLAVNER \| \| 1. LLTLLEAPFK \| \| 1. LNNAENLFQGQVK \| \| 1. QAFPNLIVANETPEATKK \| | 51.55 | Adipocyte plasma membrane-associated protein-like | XP_001605615.1_adipocyte plasma membrane-associated protein-like [Nasonia vitripennis] |
| 940 | \| 1. AKFEELNMDLFR \| \| --- \| \| 1. ALSASHQVR \| \| 1. DAGTISGLIVMR \| \| 1. EFFGGKEPSR \| \| 1. ELSDIVQPIIAK \| \| 1. EWSDATVQHDVK \| \| 1. IEIESFFEGEDFSETLTR \| \| 1. ITPSYVAFTADGER \| \| 1. KDVDEIVLVGGSTR \| \| 1. KVTHAVVTVPAYFNDAQR \| \| 1. MKETAEAYLGKK \| \| 1. NELESYAYSLK \| \| 1. NGRVEIIANDQGNR \| \| 1. NQLTTNPENTVFDAKR \| \| 1. TKMEEAIDEK \| \| 1. VATTQGDKIFAPEEISAMVLGK \| \| 1. VLEDADMSKK \| \| 1. VMDHFIK \| \| 1. WLEENQDTDPEEYKR \| \| 1. IINEPTAAAIAYGLDKK \| \| 1. EKIVITNDQNR \| \| 1. FDLTGIPPAPR \| \| 1. MEEAIDEKIK \| | 99.85 | Heat shock protein 70 | AXY94698.1_heat shock protein 70II [Habrobracon hebetor] |
| 990 | \| 1. EEVAGFTKK \| \| --- \| \| 1. IAETVKQNSGTVK \| \| 1. LWTDVKDKLNK \| \| 1. NINDLATNIK \| \| 1. QFQDGVQTLVTETNK \| \| 1. QNSGTVKEEVAGFTKK \| \| 1. SQLNLPDQETVVNTLK \| \| 1. VVEDINTSVPNAK \| | 99.49 | Hypothetical protein LOC100117157 | XP_001602474.2_hypothetical protein LOC100117157 [Nasonia vitripennis] |
| 991 | \| 1. EDFVPAIYTR \| \| --- \| \| 1. EYNHEPSVGNSMFADIAVLELK \| \| 1. KLCGLLCTKK \| \| 1. NFFSNIRDNQLCGHMR \| \| 1. NHVGQANEEVCTGSYCYERR \| \| 1. TNKFINADYVVVAGVANAHNKEEQSR \| \| 1. VTSFIPFIVSAINGGQR \| \| 1. WAEVDVR \| \| 1. YILTTAHCYMDAR \| | 90.75 | Trypsin-like | XP_016836963.1_trypsin-like [Nasonia vitripennis] |
| 1001 | \| 1. EGLDEEEKQQK \| \| --- \| \| 1. EICVVGLDLWQVK \| \| 1. FVDDSWEK \| \| 1. FWNDPDNDKGIQTSQDAK \| \| 1. GPWVHPEIDNPEYIPDQDLYK \| \| 1. IFDCSLK \| \| 1. MKEGLDEEEKQQK \| \| 1. NFKEDLEEK \| \| 1. NWIYSEHPGQEFGK \| \| 1. QFGEEIWKPTIEGEKK \| \| 1. QIENPNYK \| \| 1. QSDMHGDSPYLLMFGPDICGPGTK \| \| 1. SGTIFDNILVTDEPLYAK \| \| 1. HEQNIDCGGGYIK \| \| 1. KVHVIFNYK \| | 99.11 | Calreticulin precursor | NP_001155151.1_calreticulin precursor [Nasonia vitripennis] |
| 1034 | \| 1. AASVAIHPVFK \| \| --- \| \| 1. AAVPLLTSESYGMQYSR \| \| 1. AEVEAGDRYR \| \| 1. AFYTGIK \| \| 1. AHRMDYEETLK \| \| 1. ANAIDISLQVNGGLK \| \| 1. ASLTVQANGPETLR \| \| 1. CWHVAMTSYPK \| \| 1. DAVAQAGTGPALVNIK \| \| 1. DGQDYHVSAALESK \| \| 1. DISDQVQSPSNPQSTGNLVYSYNRPSENANSR \| \| 1. DLVVDKDVPTWEVNVLK \| \| 1. DRVSQSQVELNDNNNK \| \| 1. EDGDLISIVK \| \| 1. EFSATYALTNDDQCQGPALK \| \| 1. ELYEQQPSSNQLK \| \| 1. ESSVLSFSDLIR \| \| 1. FEEGSPLNELVMSALDMAHGASQSHLETSK \| \| 1. GANPDLSQK \| \| 1. GLCGNYDSEPENDQQTPK \| \| 1. HDIMNLKPVSNDR \| \| 1. HSVPSSAK \| \| 1. IADYTNYDSSK \| \| 1. IIFEIYEQSDK \| \| 1. ILSIFEPYLEGKK \| \| 1. ISPNNVCALDK \| \| 1. ISQEEMSSENTNNNDEANNSPNAR \| \| 1. ITLGDKEVELK \| \| 1. ITYDGQR \| \| 1. IVISGDLKK \| \| 1. KFAIQSSVTTNK \| \| 1. KGMSYDFTR \| \| 1. KIQLYSAAVGR \| \| 1. LACSVSSLFLPNAPEKSEYENYVVK \| \| 1. LLGIHGQDPEQIEGAFYMNNQR \| \| 1. LPSQYSK \| \| 1. LQVQPNEDEGDFKALQYR \| \| 1. LSNRPSEESDSR \| \| 1. MFMVLSMYNLAK \| \| 1. MYSNSADCHEIR \| \| 1. NAESLDAADITIR \| \| 1. NDPDRPSQQEQVPDDMQVSVLVK \| \| 1. NIAQEIGNEMQKPDNMPEEQTLEK \| \| 1. NIQLHCVPR \| \| 1. NNWEPGTNDNGR \| \| 1. NSACSYESVQTGNVISDEEAGRK \| \| 1. NSLTNSGFEMEGYTIGSDDSIIPK \| \| 1. QDKSDDAMNAGLK \| \| 1. QEYLAGIDKDMQVYLPMR \| \| 1. QIEGFEAAFVIDTLGK \| \| 1. QMEQSGDKITSACEK \| \| 1. QPYALFK \| \| 1. QVSTYQR \| \| 1. QYTGILMK \| \| 1. RDAWVAFR \| \| 1. RFGFITPFER \| \| 1. RQEYDAVIHYGK \| \| 1. SAHNRFPVHAFGR \| \| 1. SDNEICFSLRPVPSCSSSCSPTSTK \| \| 1. SEFEYDKNK \| \| 1. SEYENYVVK \| \| 1. SIVSQLQVDTQGENAEQSEHNQMPEGK \| \| 1. SMIESLAQLQDEEYREMADAAR \| \| 1. SSVVATAAYGQSNVDEKER \| \| 1. STQTPTPEFLDAYFDLIK \| \| 1. TLASYNPQAK \| \| 1. TMEDTVGGK \| \| 1. TMSADQIAEVQR \| \| 1. TVSVSMLMATNPPVSMLQR \| \| 1. VAVEVAPNDSQMEMTIQTPEGQIK \| \| 1. VELTGVVSSVQNLWNFAQQK \| \| 1. VGYHHGINGR \| \| 1. VLFFASANIK \| \| 1. VSPSNDLNAEVR \| \| 1. VSQSQVELNDNNNKQIIK \| \| 1. VVLNPTQR \| \| 1. YLPYLEEELDSAVK \| \| 1. YSPENVAK \| | 99.83 | Vitellogenin | ABO70318.1_vitellogenin [Pteromalus puparum] |
| 1049 | \| 1. EDVGDPLFLTPLIESGK \| \| --- \| \| 1. EMTDVSSYAGYLTVNK \| \| 1. LIDNDLTNEPSVFKK \| \| 1. NLTEVLVR \| \| 1. YDDAFVIFDK \| \| 1. YVPAVSHAIK \| | 97.62 | Venom serine carboxypeptidase isoform X1 | XP_003424792.1_venom serine carboxypeptidase-like isoform X1 [Nasonia vitripennis] |
| 1058 | \| 1. EYNPIEVHNAQR \| \| --- \| \| 1. NTIIGVVSSSPVGCREDQEPALYTR \| \| 1. PHLTYENK \| \| 1. QAIVCGYGDNWVTTR \| \| 1. TKENVEVCSGR \| \| 1. VTSFLPFITQAMTGIR \| \| 1. NTIIGVVSSSPVGCR \| | 80.77 | Trypsin-like | XP_016836963.1_ trypsin-like [Nasonia vitripennis] |
| 1059 | \| 1. FINKEYIVVAGVSNAHNKEEQYR \| \| --- \| \| 1. FPGSPEGLCTGDSGSPLVIR \| \| 1. KFALVNIISNMACR \| \| 1. KIADIAVLELK \| \| 1. NFFTDIIDSQLCSHMIQR \| \| 1. NNPHTDEIVEVCSGSNCYER \| \| 1. NTIIGVVSSSPEGCREDLEPAIYTR \| \| 1. NVRPHSTYENQQAVVCGYGHNWVAVR \| \| 1. SDLGLNQFITNVVLPR \| \| 1. VTSFIPFIIDAMNGVR \| \| 1. YIITAAHCFIDSQTNK \| | 97.01 | Trypsin-like | XP_016836963.1_trypsin-like [Nasonia vitripennis] |
| 1089 | \| 1. AHPEGLCMGDSGAPLVIGNTIIGVVSGSPR \| \| --- \| \| 1. FILTVAHCFINSR \| \| 1. GYNPIVPR \| \| 1. LATYEVIECGLFCK \| \| 1. TNQDIEVCSGSYCYER \| | 77.67 | Trypsin-like | XP_016836963.1_trypsin-like [Nasonia vitripennis] |
| 1105 | \| 1. AQESANTVNAWVNEK \| \| --- \| \| 1. AVADSAAVFSTNFFKK \| \| 1. EDITGDTR \| \| 1. ESLALPADDAVAKK \| \| 1. EVDLYLPK \| \| 1. FIELPYENKDLK \| \| 1. GTFVYGELPDLK \| \| 1. IDLKEPLEELGMK \| \| 1. MILLNAVYFK \| \| 1. MYIANDLK \| \| 1. QLTAGSFR \| \| 1. SEASELDMSK \| \| 1. SILEKEDITGDTR \| \| 1. SKGENLISSPLSAHTVLSMAAYGAGANTAK \| \| 1. TFHVDEKTEK \| | 99.63 | Serine protease inhibitor 3/4 isoform X16 | XP_008201843.1_serine protease inhibitor 3/4 isoform X16 [Nasonia vitripennis] |
| 1109 | \| 1. DAYVNFICPPEILR \| \| --- \| \| 1. DKIGGDTGPYTK \| \| 1. FEISMNGR \| \| 1. FLLGNADKADDYFR \| \| 1. GHLIADGDKLYK \| \| 1. IGGDTGPYTK \| \| 1. KIEGEDTEIK \| \| 1. MIELCHDTR \| \| 1. NEQVEFKDVK \| \| 1. TCEDMTPTTTPNVAPSMPDVR \| \| 1. TEMLADSPIVASLFTQNNPQTSTPR \| \| 1. VVDKPGVMTVK \| \| 1. YTAILNDTGK \| | 85.22 | Hypothetical protein LOC100116826 | XP_032452125.1_hypothetical protein LOC100116826 [Nasonia vitripennis] |
| 1114 | \| 1. DFPGMCFASTK \| \| --- \| \| 1. LFELVEDCGPLPK \| \| 1. QSWELTPFCGR \| \| 1. STCVPADDNSGR \| | 99.84 | Uncharacterized protein LOC100113619 | XP_001604071.1_uncharacterized protein LOC100113619 [Nasonia vitripennis] |
| 1143 | \| 1. EFHPYGGK \| \| --- \| \| 1. EVDKIYIPR \| \| 1. NQVHHCGGGILNNR \| \| 1. NTIIGVLSNGPPNCR \| \| 1. SHAHPEGICEGDSGAPLVIR \| \| 1. VDIITNIACHR \| \| 1. VLVEEFNFMNR \| | 98.65 | Trypsin-like | XP_016836963.1_trypsin-like [Nasonia vitripennis] |
| 1194 | \| 1. DMVSPCIR \| \| --- \| \| 1. DSVNRPGFSK \| \| 1. EIIMTCETPK \| \| 1. ILEAQVK \| \| 1. LFLESASEER \| \| 1. VIEYLLMR \| | 99.93 | Ferritin precursor | NP_001166224.1_ferritin precursor [Nasonia vitripennis] |
| 1233 | \| 1. AHYTVVAGIR \| \| --- \| \| 1. AIVSGFGDNWVAMRPHPR \| \| 1. ENVEPAIYTR \| \| 1. FAQVDLLSR \| \| 1. QAHNHGEQYR \| \| 1. TSGVLEVCSGSYCNELR \| \| 1. VADIAVLLLR \| | 95.12 | Trypsin-like | XP_016836963.1_trypsin-like [Nasonia vitripennis] |
| 1301 | \| 1. ALQAALNK \| \| --- \| \| 1. DIESTLTTSLDK \| \| 1. ERLLEIKDVK \| \| 1. LTGEFLDK \| \| 1. LVDLQQYTITDFVK \| \| 1. NILVEIEDLK \| \| 1. NSDYYDSKELQDKIQK \| \| 1. QSIQTLK \| | 99.02 | Venom protein U precursor | NP_001155170.1_venom protein U precursor [Nasonia vitripennis] |
| 1303 | \| 1. DKVPCIAACMIR \| \| --- \| \| 1. EKCIAEAGLDPK \| \| 1. GKESTQKELVDEVK \| \| 1. HHVITHDHKDK \| \| 1. LLENKELR \| \| 1. QNPAYPESK \| \| 1. TLQEAIEK \| \| 1. VPCIAACMIR \| | 99.73 | Odorant-binding protein 9 | QGW50304.1_odorant-binding protein 9 [Chouioia cunea] |
| 1310 | \| 1. AQPEGLCAGDSGNPLVINNTIIGVVSGSPK \| \| --- \| \| 1. DLLDSQLCGHMVQPPR \| \| 1. EDLEPAVYTR \| \| 1. FINEEYAVVAGVTR \| \| 1. GCREDLEPAVYTR \| \| 1. HIITVAHCFINLK \| \| 1. IYILQEYNPMVR \| \| 1. NNVHFCGGAILNNR \| \| 1. RFAQVNILSNMACR \| \| 1. TNKDIEVCSGSYCYER \| \| 1. YEVVDCGLFCK \| | 84.46 | Trypsin-like | XP_016836963.1_ trypsin-like [Nasonia vitripennis] |
| 1330 | \| 1. DSNGDGIGDLNGITEK \| \| --- \| \| 1. DVGADALWLSPIFSSPQK \| \| 1. EMENVLTFWMNR \| \| 1. FMILEAYTSIQK \| \| 1. GSTAIDFKK \| \| 1. LLDNFVK \| \| 1. PWLNIPADVK \| \| 1. QYFYHAFAVGQPDLNYR \| \| 1. SGLKEGESLNLTK \| \| 1. SMQFYSVGSNPFNFMFIASLNK \| \| 1. SVDGIEPYKDYYIWR \| \| 1. TIEGWLSAVPR \| \| 1. TLNLATQK \| \| 1. TPFQWDDTTSAGFSSNTK \| \| 1. TSEISHYK \| \| 1. TSVIYQIYPR \| \| 1. TWLPVHDNYK \| \| 1. VANWVVGNHDNHR \| \| 1. VDAVNHMFEDEQLR \| \| 1. KSVDGIEPYK \| \| 1. VFQALTK \| | 98.96 | Alpha-glucosidase-like | NP_001153339.1_alpha-glucosidase-like [Nasonia vitripennis] |
| 1372 | \| 1. ADGTIDVETAR \| \| --- \| \| 1. AVIDGIIK \| \| 1. DEKLDCFSACMLKK \| \| 1. EACITEVGADKAVIDGIIK \| \| 1. LDCFSACMLKK \| \| 1. LREYKEACITEVGADK \| \| 1. NACETGGNVFGCFIK \| \| 1. RDEKLDCFSACMLK \| | 99.79 | General odorant-binding protein 56d | XP_001601182.1_general odorant-binding protein 56d [Nasonia vitripennis] |
| 1432 | \| 1. KLSDSAWEK \| \| --- \| \| 1. LTSTLGDGPALPNCNAK \| \| 1. MNFNQLPHFK \| \| 1. QLATEALR \| \| 1. TLAGHASDLK \| \| 1. VLELTELHSLAK \| | 99.90 | Ferritin heavy polypeptide-like 17 | XP_008203832.1_ferritin heavy polypeptide-like 17 [Nasonia vitripennis] |
| 1617 | \| 1. ACQNLLTDIKDSHLCGHLVQR \| \| --- \| \| 1. AFFTIELPNFPR \| \| 1. EDLRPAVYTR \| \| 1. FINSEYTVVAGATYAHK \| \| 1. RTEVFSGSYFYQR \| \| 1. SPLNPEGLCTGDSGSPLVTGETIIGVVSYSPTGCR \| \| 1. VTPFIPFITNAMSGVR \| \| 1. YILTVAHPFIDPNNR \| \| 1. DSHLCGHLVQR \| \| 1. VAKYEVISCGR \| | 91.07 | Trypsin-like | XP_016836963.1_ trypsin-like [Nasonia vitripennis] |
| 1636 | \| 1. AFMAIMLPNFPR \| \| --- \| \| 1. ANPEGLCAGDSGSSLVIHNTIIGVVSTSPEGCR \| \| 1. HFITDLKDSHLCGHMVQR \| \| 1. NAEYTVVAGVHQAHNKEEPHRVER \| \| 1. NVGGGGILNNR \| \| 1. RFAEVNILSNMECR \| \| 1. VTSFIPFITNAMSGVR \| \| 1. YEVGMYNMK \| \| 1. YIITAAHFFIDSR \| | 91.32 | Trypsin-like | XP_016836963.1_ trypsin-like [Nasonia vitripennis] |
| 1650 | \| 1. DTNGSQFFITVK \| \| --- \| \| 1. LQHYGAGWLSMANAGK \| \| 1. QTPWLDGR \| \| 1. SIYGDRFEDENFK \| \| 1. TVENFFQLAK \| \| 1. VIRDFMIQGGDFTKGDGTGGK \| | 99.90 | Peptidyl-prolyl cis-trans isomerase 5 | XP_014237978.1_peptidyl-prolyl cis-trans isomerase 5 [Trichogramma pretiosum] |
| 1651 | \| 1. ESLDYIHIAEPGDYK \| \| --- \| \| 1. GHYLISHQGNR \| \| 1. LGVLGFLSTEDK \| \| 1. LNPFTPFGPVVEK \| \| 1. NIESFGGDPNK \| \| 1. QNLLSLTHLVGDR \| \| 1. SSDDPFINR \| \| 1. TATDYAMHK \| \| 1. YEAYEGVPYALPPVGDKR \| | 99.67 | Venom carboxylesterase-6 | XP_001603114.1_venom carboxylesterase-6 [Nasonia vitripennis] |
| 1652 | \| 1. AAQSLSDVMSGTK \| \| --- \| \| 1. ELNYLHISGPNK \| \| 1. FSIEYNDNLGEK \| \| 1. GVPDFGVEWPEVNPTK \| \| 1. HYLGLKPIDR \| \| 1. KIPPWTGELQATK \| \| 1. LFVVDAEK \| \| 1. LGALMGCPTDNTMEMIQCLK \| \| 1. LGSLGFLSTEDEVVPGNMGLK \| \| 1. LLTLMVGDR \| \| 1. LYEAYEGIPYALPPVGK \| \| 1. NINFNENK \| \| 1. TNKEYFINK \| \| 1. VSDACLQYTHIPAVQNER \| \| 1. WVHDNIEWFK \| \| 1. DQSMALR \| | 97.04 | Carboxylesterase clade E, member 11 precursor | NP_001165965.1_carboxylesterase clade E, member 11 precursor [Nasonia vitripennis] |
| 1747 | \| 1. DAQDPFAK \| \| --- \| \| 1. DEATDFLEQDTIR \| \| 1. DSANFAEHVEQLMR \| \| 1. EALRTDEQVVQR \| \| 1. EEEAIKIDGLNVAQIK \| \| 1. ELISNASDALDKIR \| \| 1. ESEIDTDDNEDKEESEDHDEL \| \| 1. FNFQTEVNR \| \| 1. GYEVLYLVEAVDEYALSAIPEFDGKK \| \| 1. ILSIMDTGIGMTK \| \| 1. KDEDEDAKVEEANDEK \| \| 1. KQELENIFEPLIK \| \| 1. KTLGIPLDETTEDDDVSENVHETEK \| \| 1. LGVIEDAQNR \| \| 1. LIINSLYR \| \| 1. LLSLTDKNVLETNPELAIR \| \| 1. LTDSPCALVASMFGWTGNMER \| \| 1. MKQNQQHIYYIAGSSIDEVKK \| \| 1. QDLVNNLGTIAK \| \| 1. SGTAEFLGK \| \| 1. SLLFIPK \| \| 1. SSASEKPTLLADYISR \| \| 1. TDEQVVQREEEAIK \| \| 1. TLEINPR \| \| 1. TVEVEEEEKQDKIIEEK \| \| 1. TYYLNQK \| | 99.88 | Endoplasmin | XP_001599282.1_endoplasmin [Nasonia vitripennis] |
| 1794 | \| 1. AVVSGYGHNWVIMR \| \| --- \| \| 1. NAMNGVQAADMR \| \| 1. RDDQTHVVVEDCSGSYCYER \| \| 1. SPNNPEGLCTGDSGAPLIMGNTIIGVASTSPDGCREDSKPSIYTK \| \| 1. VTSFLPFIR \| \| 1. YEIISCGR \| | 96.58 | Trypsin-like | XP_016836963.1_trypsin-like [Nasonia vitripennis] |
| 1835 | \| 1. AGEWDTQTKNEIFPHQDR \| \| --- \| \| 1. DNEAQFGEFPWMVAILK \| \| 1. DTCKGDGGSPLVCPVK \| \| 1. EGHYQVILKR \| \| 1. GDGGSPLVCPVK \| \| 1. LNVYQCGGALIHPR \| \| 1. NEIFPHQDR \| \| 1. NPEGVGFR \| \| 1. PWIDQQLR \| \| 1. QVAQVVVHER \| \| 1. RVELPVVPR \| \| 1. VQAGQCDNYLDVCCAPPDVRPPDQK \| \| 1. VVLTAGHCVNGK \| \| 1. YFTLDQTFICAGGESGKDTCK \| | 99.90 | Serine protease homolog 21 precursor | NP_001155060.1_serine protease homolog 21 precursor [Nasonia vitripennis] |
| 1855 | \| 1. HFVPDISDSQICGSIR \| \| --- \| \| 1. NTIIGVASNAARGCR \| \| 1. NWISIHR \| \| 1. QYPSYANQQAVVCGYGR \| \| 1. SPANPEALCLGDSGAPLVIR \| \| 1. TSDIAILK \| | 96.70 | Trypsin-like | XP_016836963.1_trypsin-like [Nasonia vitripennis] |
| 1890 | \| 1. GAYSPVCGSDGR \| \| --- \| \| 1. IISYQPCYQSNSALVSGQCPCPEK \| \| 1. ILQCVNK \| \| 1. KNIQIVSYR \| \| 1. SYASAGTLQCYNR \| \| 1. TYENPEIVR \| | 98.86 | Ovomucoid-like | XP_008214430.1_ovomucoid-like [Nasonia vitripennis] |
| 1907 | \| 1. DCDNAGTPNEICAER \| \| --- \| \| 1. DSCQGDSGGPLQTTTVYNNQGR \| \| 1. ICGTEGFPGVYTR \| \| 1. LLDHNICGAIGTDR \| \| 1. MHNDIAVIR \| \| 1. SQCGFEGNDPK \| \| 1. TGGPNPEFR \| \| 1. YILTAAHCVTNLPSTLR \| \| 1. YQDFTIDQVIYHPQYSPVK \| \| 1. YVQYGVVSFGTR \| | 99.64 | Serine protease 22 precursor | NP_001155043.1_serine protease 22 precursor [Nasonia vitripennis] |
| 1971 | \| 1. ATIVGWGVSNR \| \| --- \| \| 1. EAGPCIR \| \| 1. HYCGGSLITR \| \| 1. KHVVTAAHCLFTDESVFSGGVEER \| \| 1. LDMTIQSVEDCR \| \| 1. QTALILQK \| \| 1. VIPIALPLSRPAVGQK \| | 83.40 | Chymotrypsin-1-like | XP_023314019.1_chymotrypsin-1-like [Trichogramma pretiosum] |
| 2025 | \| 1. ALHVPDDLGKVDVQR \| \| --- \| \| 1. DILDNGMPSR \| \| 1. DVFFLNK \| \| 1. EAMQQLTDAR \| \| 1. FDFALEALKK \| \| 1. FLPEMTKK \| \| 1. FYALEQALEAER \| \| 1. KALHVPDDLGK \| \| 1. LRDCMLSFFGDELQR \| \| 1. LWLSSSK \| \| 1. NTVDENTDAVLANAVYFK \| | 97.17 | Serpin 5 precursor | NP_001161183.1_serpin 5 precursor [Nasonia vitripennis] |
| 2057 | \| 1. ALGISVVHCGVEDIDEEAFK \| \| --- \| \| 1. FDVDAMK \| \| 1. GVSTPGSHDAYTCLR \| \| 1. IEKDAFQK \| \| 1. IIEYSNANPGLVK \| \| 1. KDDQKELATCVLAK \| \| 1. KLESLDLSGNVIR \| \| 1. LLEGEKPVEPIK \| \| 1. LVNVAFR \| \| 1. NSYASVDGIRDTYK \| \| 1. VGLQWNPYEWSQGVK \| | 99.13 | Insulin-like growth factor-binding protein complex acid labile subunit | XP_001603643.1_ insulin-like growth factor-binding protein complex acid labile subunit [Nasonia vitripennis] |
| 2095 | \| 1. DQNLVLR \| \| --- \| \| 1. FLTECSYNK \| \| 1. GIPYAEPPIGYLR \| \| 1. HETLLFTGSISSILDLLYTATNYIK \| \| 1. IDENLSVGK \| \| 1. ILYETPAFR \| \| 1. LGPLGFLSLNHDDATGNAGLK \| \| 1. LGSVNPPFKPTIER \| \| 1. LIEEAEK \| \| 1. LTEVVNTSK \| \| 1. MIPSLSPIPIPFLDK \| \| 1. NIINFGGDPQK \| \| 1. TSWNNVLK \| \| 1. TTIEGANCVQK \| \| 1. YGNPTPEGEKDNLLQGIIWPESGK \| \| 1. MWTNFAK \| | 97.15 | Carboxylesterase clade B, member 2 precursor | NP_001155148.1_carboxylesterase clade B, member 2 precursor [Nasonia vitripennis] |
| 2101 | \| 1. FLHTVESGVQR \| \| --- \| \| 1. LAEVSNMLLTK \| \| 1. LFNFCDPIDSGK \| \| 1. LIGQWLEKDK \| \| 1. QYGAMCFLLEHR \| \| 1. SKYPHLVHGAMSASGPLLAK \| \| 1. TYSQACVDTIAAAIK \| \| 1. WVVEGQMVEYAK \| \| 1. YFMNADYYKK \| \| 1. YNNMVEEMR \| \| 1. YYGMSHPTPDLSVK \| | 98.72 | Putative serine protease K12H4.7 | XP_031779307.1_putative serine protease K12H4.7 [Nasonia vitripennis] |
| 2176 | \| 1. AITNALDNGYR \| \| --- \| \| 1. DLPNLTELQVVK \| \| 1. EIPQVGLGTSTIKPDELEK \| \| 1. GGKREDLFITSK \| \| 1. HAVQEGLVVIPK \| \| 1. HIDTAFAYDNEAGIGK \| \| 1. IVMTAYAPLGSYSSR \| \| 1. KIEALDKGEK \| \| 1. LPSQGNRPASVEK \| \| 1. LTDEEMK \| \| 1. NAEIKPSNLQVESHAYLQQR \| \| 1. QKQNIDLFNFK \| \| 1. SIGLSNFNQAQVLNVYK \| \| 1. TPSQILLR \| | 96.87 | Alcohol dehydrogenase [NADP(+)] B-like | XP_014219296.1_ alcohol dehydrogenase [NADP(+)] B-like [Copidosoma floridanum] |
| 2201 | \| 1. EIDEQKQDIHWWK \| \| --- \| \| 1. EQTAGKPVVLLINFK \| \| 1. KSEISHFK \| \| 1. LTDEPPSGANVPANDYDYFNHVYTR \| \| 1. NEIDNWLTSIPANK \| \| 1. NSMLDQEMK \| \| 1. NYYIWQDGKK \| \| 1. QYYYHAFVAGQPDLNYR \| \| 1. SFKDSNGDGIGDLK \| \| 1. TILNTPDSLK \| \| 1. TSTAFDFK \| \| 1. TSVIYQVYPR \| \| 1. VDAVNYLFEDEK \| \| 1. VEIADDNGDVLAVIR \| \| 1. VLIPSVNSK \| \| 1. VMILEAYTSLDNSMK \| \| 1. TLNLAAQK \| | 99.55 | Alpha-glucosidase-like | NP_001153339.1_alpha-glucosidase-like [Nasonia vitripennis] |
| 2219 | \| 1. AASVPGTPTAQQPAGTPAGR \| \| --- \| \| 1. ENEYISPAQR \| \| 1. FNIVTHK \| \| 1. GNTVTDEDLEK \| \| 1. KPAPGTEVGLDIGLPKPK \| \| 1. NPGLGGGSGQGYR \| | 99.66 | Poly(U)-specific endoribonuclease homolog | XP_001607440.2_poly(U)-specific endoribonuclease homolog [Nasonia vitripennis] |
| 2283 | \| 1. HIITVAHCFIGEK \| \| --- \| \| 1. NVMDSQLCGNIIQR \| \| 1. RFADIAVLELK \| \| 1. TNRFDSEEYIVVAGVTNAHNLQGR \| \| 1. VTSFIPFIVNAIR \| \| 1. YEVLSCGR \| | 98.42 | Chymotrypsin-1-like | XP_008214285.1_chymotrypsin-1-like [Nasonia vitripennis] |
| 2298 | \| 1. FVTSAGDIEETWK \| \| --- \| \| 1. IIVWEMNR \| \| 1. LYHELGVR \| \| 1. NVPDDVLHTVK \| \| 1. SCMTDLPR \| \| 1. SPVIFSHSSAFALCQNYR \| \| 1. SQPPYQNIITGQELK \| \| 1. WSREDLEK \| \| 1. YPALFDR \| | 82.47 | Dipeptidase 1 isoform X1 | XP_031784812.1_dipeptidase 1 isoform X1 [Nasonia vitripennis] |
| 2399 | \| 1. FENANYIVVAGVHQAHR \| \| --- \| \| 1. IYIPGQYNSIR \| \| 1. RDAQTEVCSGSHCHER \| \| 1. RFAQVDILPNNACR \| | 90.49 | Trypsin-like | XP_016836963.1_ trypsin-like [Nasonia vitripennis] |
| 2538 | \| 1. AYQVVDK \| \| --- \| \| 1. EQHPAVAVLEK \| \| 1. NAWILTR \| \| 1. QISSLSGVSSSISGHAMQK \| \| 1. VDTAEDFDTQK \| | 99.90 | Apolipoprotein D-like | XP_011504442.1_ apolipoprotein D-like [Ceratosolen solmsi marchali] |
| 2618 | \| 1. ANGDNVDKCQALFDLK \| \| --- \| \| 1. DDLNNFEDIIK \| \| 1. INESSVMLTEFK \| \| 1. LTGEQLNK \| \| 1. NSLLIEGK \| \| 1. VEWLVHK \| | 98.00 | NA | NA |
| 2622 | \| 1. GVGTSTQPIR \| \| --- \| \| 1. HSGIIFSDLQR \| \| 1. LDLFGACSVGFR \| \| 1. LYQLDLLGIK \| \| 1. LYWFSADLTK \| \| 1. NIYVYDETTEK \| \| 1. VQYANIDATDFK \| | 95.68 | Low-density lipoprotein receptor-related protein 6-like | XP_001601339.2_low-density lipoprotein receptor-related protein 6-like [Nasonia vitripennis] |
| 2736 | \| 1. ADIANYVALLK \| \| --- \| \| 1. CNVFYYCQPFNNK \| \| 1. DGFGNFNK \| \| 1. EAFDKEGLILSAAVAAAEK \| \| 1. FLIDNIDPTLCTHLIYTFVGISESGDVK \| \| 1. FVQNVVAFVK \| \| 1. GDQWVGYDNQQSLTEK \| \| 1. LILGVPLYGR \| \| 1. LREISPESK \| \| 1. SASLSYDIPNVSK \| \| 1. TGINAPLYAGSWETDAER \| \| 1. TMIAIGGWNEGSVK \| \| 1. VLDSWQDLPDDSGKDGFGNFNK \| \| 1. VVANPEIR \| | 99.68 | Chitotriosidase-1 isoform X1 | XP_008210079.1_chitotriosidase-1 isoform X1 [Nasonia vitripennis] |
| 2872 | \| 1. APLEYLEGGK \| \| --- \| \| 1. FAEVTNCPPENSQQLLNCLK \| \| 1. IILAHAER \| \| 1. KHYLGDKPISK \| \| 1. NYEAYEGIPFAKPPTGNLR \| \| 1. TFAELSTSYAK \| \| 1. WVNNNIQYFGGDPK \| \| 1. DQNLALR \| | 96.57 | Venom carboxylesterase-6-like | XP_016839500.1_ venom carboxylesterase-6-like [Nasonia vitripennis] |
| 2910 | \| 1. LIYQENIVKPSK \| \| --- \| \| 1. SHDLIIGSR \| \| 1. TAGGPGYSNITLK \| \| 1. TFGTGPAYAITQVR \| \| 1. WLQVIEVSK \| | 99.03 | Venom protein R precursor | NP_001155164.1_venom protein R precursor [Nasonia vitripennis] |
| 3012 | \| 1. DFVEIVCCPK \| \| --- \| \| 1. HVLTAAHCVNNINNYVPIEIR \| \| 1. IPVAEVVAHPQHR \| \| 1. SLNYYDIAILK \| \| 1. TPTLSIIDR \| \| 1. VGSVNLDDNTK \| \| 1. YNCGGTLISVR \| | 90.42 | Serine protease persephone isoform X2 | XP_008206147.2_ serine protease persephone isoform X2 [Nasonia vitripennis] |
| 3120 | \| 1. FDTGQNLAQTK \| \| --- \| \| 1. IVTLHNTLR \| \| 1. SMPNLEWDEELAK \| \| 1. VISSGLSNEEQNK \| \| 1. WADQCEYEHDCSPIER \| | 99.84 | Venom allergen 5-like | XP_031786283.1_venom allergen 5-like [Nasonia vitripennis] |
| 3239 | \| 1. FRPPEPTQPWK \| \| --- \| \| 1. LATLVNCPTGDAK \| \| 1. LFLVDAEK \| \| 1. LGPEWTPVDAR \| \| 1. NLDQLTHIATDR \| \| 1. QHYLNDKPTNYK \| \| 1. THIQAFGGDPTK \| | 97.20 | Carboxylesterase clade E, member 11 precursor | NP_001165965.1_carboxylesterase clade E, member 11 precursor [Nasonia vitripennis] |
| 3257 | \| 1. EEVSDYLR \| \| --- \| \| 1. EIDADKDNQLSR \| \| 1. KLVIPPALGYGDR \| \| 1. LVEEIFQHEDKDK \| \| 1. LVIPPALGYGDR \| \| 1. NGFISHEEFSGPK \| \| 1. RQMQEAEQSGAGESDEMKK \| \| 1. VFVPEVCDVK \| | 99.42 | FK506-binding protein 2 isoform X2 | XP_001599993.2_FK506-binding protein 2 isoform X2 [Nasonia vitripennis] |
| 3464 | \| 1. FTDTDSLKR \| \| --- \| \| 1. QAPGVDDPQSALETAFEWR \| \| 1. TDELLLR \| \| 1. YLDWVYPTVQR \| | 99.42 | Uncharacterized protein LOC100116563 | XP_001601022.2_uncharacterized protein LOC100116563 [Nasonia vitripennis] |
| 3591 | \| 1. ERYDDYLGAYEEKR \| \| --- \| \| 1. HGDRAPELYPQR \| \| 1. LFAVSSGLAR \| \| 1. MYGLGELLR \| \| 1. NELKPYESYLNNLR \| \| 1. TATGYLFSYITEDMEK \| \| 1. VTLNDINLLHNNLDLER \| | 98.70 | Venom acid phosphatase Acph-1-like | XP_014205848.1_venom acid phosphatase Acph-1-like [Copidosoma floridanum] |
| 3708 | \| 1. AYNPMVVSADGNTR \| \| --- \| \| 1. DLHYCGGGILSNR \| \| 1. NILDSQLCGHMNQR \| \| 1. RFAQVNILPNRR \| | 98.70 | Trypsin beta-like | XP_031777966.1_trypsin beta-like [Nasonia vitripennis] |
| 3779 | \| 1. LSLIEPVDFR \| \| --- \| \| 1. LTPDGILHEAPSTTVTLLPSGK \| \| 1. RADEDTYTSTAPAK \| \| 1. VVPTSPFVQAPPIR \| | 99.76 | Uncharacterized protein LOC100680448 isoform X1 | XP_031782833.1_  uncharacterized protein LOC100680448 isoform X1 [Nasonia vitripennis] |
| 3815 | \| 1. EQITLLPLETSINAR \| \| --- \| \| 1. GIVLVDYRER \| \| 1. IILAYNETLFK \| \| 1. MLFDAAAK \| \| 1. MLTIMGRDDIPVYGGAK \| \| 1. NVDVVTTDGPAK \| \| 1. QGGPYIDFLNK \| \| 1. VEAIGLSK \| \| 1. YAANALIDIVNGNK \| | 99.81 | Inosine-uridine preferring nucleoside hydrolase-like precursor | NP_001155173.1_inosine-uridine preferring nucleoside hydrolase-like precursor [Nasonia vitripennis] |
| 3847 | 1. ENNAILPTGGSIYR 2. SDPSSSTYTLQIK 3. SREDQLPISTNTALIIR 4. TPTISYISQEQIK | 98.15 | Lachesin isoform X2 | XP_031785062.1_lachesin isoform X2 [Nasonia vitripennis] |
| 3848 | \| 1. ALNWLVEK \| \| --- \| \| 1. ALYDNFQR \| \| 1. DASQIENMTPER \| \| 1. KYVEPDGYEQK \| \| 1. LNYETAGK \| \| 1. NEVNYLSYVEMTSLSGK \| \| 1. PSVVHESHQQSNAPTSHSDPYGR \| \| 1. RNEENSFLDEIVK \| \| 1. RPSSTSVSRPSAPTYSHPVR \| \| 1. TAEAPATPANFISDEDLMK \| | 97.16 | Poly(U)-specific endoribonuclease homolog isoform X2 | XP_032457640.1_poly(U)-specific endoribonuclease homolog isoform X2 [Nasonia vitripennis] |
| 3878 | \| 1. ENELSSFMQK \| \| --- \| \| 1. GTILQYLLYGYNEK \| \| 1. HGDRAPGNFIK \| \| 1. IFVPQMCVEYQK \| \| 1. IRENELSSFMQK \| \| 1. LLINVFPTEEELKCNR \| \| 1. LLYYHGIPAK \| \| 1. LSGGVILRK \| \| 1. MIEDMKNVIR \| \| 1. SNYDGFLGPVYLPNQVEAISSDSPR \| \| 1. TDVMQIPGCSQLCSFDDFVK \| \| 1. TNWQPFLTSFLPTQLDK \| \| 1. TSPISLLDMNHLYQTLSAEK \| | 74.73 | Venom acid phosphatase Acph-1 | XP_031787723.1_venom acid phosphatase Acph-1 [Nasonia vitripennis] |
| 4156 | \| 1. AVELMDR \| \| --- \| \| 1. DAFWAYEYKK \| \| 1. HGTCALTSPATNSVPK \| \| 1. KHVQFCSNSVR \| \| 1. KWAEAGSATCSPCSAAK \| \| 1. SFKPIDCPNQSLSSCNR \| \| 1. YNIATLIAR \| \| 1. YQPDLISSSLR \| | 99.12 | Ribonuclease Oy-like | XP_016839503.2_ribonuclease Oy isoform X2 [Nasonia vitripennis] |
| 4479 | \| 1. DLDSPYHK \| \| --- \| \| 1. FYDTFTCIK \| \| 1. LMCIINIK \| \| 1. LVDNADNYFK \| \| 1. SCSYFNQR \| \| 1. SSVPGIIAGGQGR \| \| 1. TENEFVPGQLSHPSYFIYK \| \| 1. FEVKPEK \| | 94.80 | Endonuclease-like venom protein precursor | NP_001155087.1_endonuclease-like venom protein precursor [Nasonia vitripennis] |
| 4531 | \| 1. ADGTINHKPEDSEQKTACK \| \| --- \| \| 1. ELECFSACLLK \| \| 1. GVMKADGTINHKPEDSEQK \| \| 1. IMECFSMNGLIPK \| \| 1. KEIDDCIAESGVTK \| \| 1. NLMDDIKNHR \| \| 1. NLVGKDDCETGGK \| | 99.66 | General odorant-binding protein 56d | XP_014204137.1_ general odorant-binding protein 56d [Copidosoma floridanum] |
| 4663 | \| 1. EKVDEVINMCK \| \| --- \| \| 1. IMREDGTIDEEVAR \| \| 1. TACITETGIDGAIIEK \| \| 1. VLGCLLK \| | 99.75 | General odorant-binding protein 56d | XP_001601068.1_ general odorant-binding protein 56d [Nasonia vitripennis] |
| 4724 | \| 1. AFYNNIK \| \| --- \| \| 1. AGAESGWDFSAR \| \| 1. DWDFVDR \| \| 1. EFNYWQR \| \| 1. IGDPHYR \| \| 1. ILSNFYR \| \| 1. KTDFAPAMNVAK \| \| 1. MTTDVRDNPSR \| \| 1. NLAILEK \| \| 1. NLALTWLR \| \| 1. QQVESFVR \| \| 1. SNYLGFNQSSK \| \| 1. SQPPLLIPMIDK \| \| 1. TDFAPAMNVAK \| \| 1. TVQLAAIYK \| \| 1. TYGFIPNGGR \| \| 1. YDATEPGK \| | 99.88 | Trehalase | XP_008215783.2_trehalase [Nasonia vitripennis] |
| 4813 | \| 1. DAVLFTTDGAGR \| \| --- \| \| 1. DSFLNTQGWGK \| \| 1. FQPQITSYDYDAPITEAGDLTPK \| \| 1. SVDDTHLAR \| \| 1. VGPQITLYVPAAYLK \| \| 1. VNFGSVDVEDFK \| \| 1. YIHYAER \| \| 1. YLEQILTK \| | 99.87 | Beta-galactosidase | XP_001603160.1_beta-galactosidase [Nasonia vitripennis] |
| 4917 | \| 1. DGLISYEEFLNESK \| \| --- \| \| 1. EVVQALESDPEFR \| \| 1. HKEHEPLHHPGSK \| \| 1. IANELEYVSHHVR \| \| 1. IPEHLDHANPHTFEIDDLKK \| | 98.29 | Nucleobindin-2 isoform X2 | XP_016844102.1_nucleobindin-2 isoform X2 [Nasonia vitripennis] |
| 5058 | \| 1. DYTHVPQFALHDIAVVK \| \| --- \| \| 1. FVENVYLHK \| \| 1. ILGGDSDDNNDFK \| \| 1. IVAGTNTLDNDDGIYK \| \| 1. LDSALDLKDPR \| \| 1. LSTRPYEDLVGK \| \| 1. NPYGGQVR \| \| 1. NTVIGVVSR \| \| 1. QHVLTAAHCFK \| | 95.41 | Chymotrypsin-2-like | XP_031777810.1_chymotrypsin-2-like [Nasonia vitripennis] |
| 5249 | \| 1. FGQEISNALR \| \| --- \| \| 1. GYAGVQVSPIQENK \| \| 1. NCELVGLHDLDQSQEHVR \| \| 1. SGNEEEFR \| \| 1. VMSSFGFDNFER \| | 99.59 | Alpha-amylase | QAV56506.1_ alpha-amylase [Pteromalus puparum] |
| 5267 | \| 1. ITGSYSTDLEHNDFIINNLK \| \| --- \| \| 1. NIESVFQK \| \| 1. NLFSSEITSYFK \| \| 1. MSMNFDIPQAPAYK \| | 99.46 | Uncharacterized protein LOC107980689 | XP_031786091.1_uncharacterized protein LOC107980689 [Nasonia vitripennis] |
| 5291 | \| 1. AEERENIALEK \| \| --- \| \| 1. DEALFFLINR \| \| 1. GMSEYEAEEYFK \| \| 1. ILDSDGSGTVTITELQTR \| \| 1. LLSVSEPNR \| \| 1. QPAVQYDEETQALVDEAR \| \| 1. SSLYRPGADFECLDGSQLITFGK \| \| 1. YKPAPSSQQAADAEGEEK \| | 99.53 | Glucosidase 2 subunit beta | XP_008208849.1_ glucosidase 2 subunit beta [Nasonia vitripennis] |
| 5399 | \| 1. CYDTLAPLLTGPENAQALK \| \| --- \| \| 1. EFISGFK \| \| 1. GNYLDTLAR \| \| 1. GQCSIATANFPIMIK \| \| 1. LENIETTIK \| \| 1. NPIEVQGMK \| \| 1. QAHIFVNDSR \| \| 1. SCFETMEK \| \| 1. TLHFGEPTDYER \| \| 1. TVITEISGADKLEK \| | 93.65 | Xaa-Pro aminopeptidase ApepP | XP_001599945.1_xaa-Pro aminopeptidase ApepP [Nasonia vitripennis] |
| 5412 | \| 1. ELSTNAEGPR \| \| --- \| \| 1. HAAISTLLK \| \| 1. KDDIDEDIAK \| \| 1. LIHNPDYFNDENPFK \| \| 1. SPETVGPALLAESFDDKER \| \| 1. TPYTIMFGPDK \| \| 1. TVEDGNFGLVLK \| \| 1. WLPPLVNNPNYK \| \| 1. YDGVWVVEEPK \| | 97.74 | Calnexin isoform X2 | XP_008215933.1 _calnexin isoform X2 [Nasonia vitripennis] |
| 5502 | \| 1. DFYTDGVCLAK \| \| --- \| \| 1. DILATDNK \| \| 1. DILEEVTR \| \| 1. ETYDGSDYSTDR \| \| 1. GTEGFGGFLGLELR \| \| 1. LDDYLLDIHAK \| \| 1. LKDILATDNK \| \| 1. LVLLETAMNDFSIAISIIDSHFDDLLLYR \| \| 1. LYEHYPIPASHFVLPGDPHDIR \| \| 1. TLNHEGEYKEGVNVAK \| \| 1. VPLGCTGNFR \| \| 1. KYSPDNNLLPNER \| \| 1. SSVFIFR \| | 59.38 | Uncharacterized protein LOC108911535 isoform X1 | XP_018572018.1_uncharacterized protein LOC108911535 isoform X1 [Anoplophora glabripennis] |
| 5573 | \| 1. FLIDGFPR \| \| --- \| \| 1. GTVCQNIVDK \| \| 1. KPQILFVLGGPGAGK \| \| 1. QGSQYGELIETHIK \| \| 1. SDDNMESLLKR \| \| 1. TYVNDTMPIIEHYK \| | 98.83 | Chemosensory protein 3 | QGW50250.1_chemosensory protein 3 [Chouioia cunea] |
| 6082 | \| 1. ANQQMASFR \| \| --- \| \| 1. DIIVQSAPSSGPSR \| \| 1. IEDHAVIMATELKR \| \| 1. MGFSYICR \| | 94.03 | Endonuclease-like venom protein precursor | NP_001155087.1_endonuclease-like venom protein precursor [Nasonia vitripennis] |
| 6126 | \| 1. DAPDGKYPYQVSLR \| \| --- \| \| 1. LGGASPNHLQEINLK \| \| 1. LVNDVAVVR \| \| 1. VVVGTNLLHGGEEK \| \| 1. YPYQVSLR \| | 99.90 | Chymotrypsin-2-like | XP_031788471.1_chymotrypsin-2-like [Nasonia vitripennis] |
| 6303 | \| 1. ALAEADILYDQVSYPDEAHGLSHVTK \| \| --- \| \| 1. DEENVFK \| \| 1. FVTPLADGR \| \| 1. FYLSPDKNYVLIR \| \| 1. GTAMIYAVYR \| \| 1. HLYHTMDK \| \| 1. HSSLSTFGIYSIR \| \| 1. IMPQQMDLVVESHGFDAR \| \| 1. KYPLLVNVYAGPDSQR \| \| 1. LLLPEDFDKSK \| \| 1. LLSIAFWAPK \| \| 1. QLYEVGIEVPK \| \| 1. SLEQESGPGAR \| \| 1. SNYFDEASNQK \| \| 1. TLGQFSHVTR \| \| 1. TVVATWTNR \| \| 1. VMGLPTPSDNLK \| \| 1. VTLNLVDLVDHGSSPISLK \| \| 1. VVYYTASPPGEPSQK \| \| 1. VYDYDHDELYTWSTNPELR \| \| 1. YGNPGDLKDQYPKEEK \| | 72.69 | Venom dipeptidyl peptidase 4 isoform X2 | XP_008204459.1_venom dipeptidyl peptidase 4 isoform X2 [Nasonia vitripennis] |
| 6382 | \| 1. AEIYIADPEYCQTVMK \| \| --- \| \| 1. NNVCANDPTIRR \| \| 1. VAEPFIFNKR \| \| 1. VAPVPLPLLHSK \| \| 1. YDPYQTQNDIALIK \| | 99.78 | Serine protease 43 precursor | NP_001166062.1_serine protease 43 precursor [Nasonia vitripennis] |
| 6782 | \| 1. CQSPVVDLEVR \| \| --- \| \| 1. FNQGTLFATDDNSFPYATCK \| \| 1. GFFMYVR \| \| 1. GGILLVTPGCHVK \| \| 1. GKELLTDKFGSDDLEQR \| \| 1. HIEVILDSK \| \| 1. IAFSNAHELFSICR \| \| 1. IGLDGQPK \| \| 1. IGNAKPFLPSILGYSMPVSAASPEK \| \| 1. IITLPDNLNWLSVR \| \| 1. KLATVNGLDLIK \| \| 1. LPETETGTSTNIHCSQFDADGK \| \| 1. LTYDLNGKK \| \| 1. TSNLNFPIDK \| \| 1. VSILNMVNCK \| | 93.78 | Uncharacterized protein LOC100680003 | XP_008205889.1_uncharacterized protein LOC100680003 [Nasonia vitripennis] |
| 7280 | \| 1. AIMAVASQEFWR \| \| --- \| \| 1. GQEGFGGFLGLK \| \| 1. HVRDDYNTAVR \| \| 1. KNPHVFDDTR \| \| 1. MLLDDTTASYLTK \| \| 1. MSQLQLLDSLLVK \| \| 1. NLPAIFDHVTK \| \| 1. NTLESVIDYDIYEVQR \| \| 1. VAMAQYYMTK \| \| 1. VGELSCPDGK \| | 95.50 | Uncharacterized protein LOC106783674 isoform X2 | XP_014597933.1_PREDICTED: uncharacterized protein LOC106783674 isoform X2 [Polistes canadensis] |
| 7398 | \| 1. AAGDDCKFLQCIK \| \| --- \| \| 1. APFAGILLDASEK \| \| 1. DDQIPENK \| \| 1. DNSGCFTEQGLDVDFSK \| \| 1. EMLETATDCKTK \| \| 1. RKEMLETATDCK \| \| 1. VLTPVEDKVACAMSCMYHK \| | 99.90 | Putative odorant binding protein 47 | CCD17816.1_putative odorant binding protein 47 [Nasonia vitripennis] |
| 7646 | \| 1. AGYEPCIAEAR \| \| --- \| \| 1. ASDNWGLMIFK \| \| 1. DYIEALR \| \| 1. LDVVAMPMYTATK \| \| 1. LPLVTVTR \| \| 1. QSTYKPALLTK \| \| 1. WNIPLVVQSR \| | 97.90 | Aminopeptidase N isoform X2 | XP_008216015.1_aminopeptidase N isoform X2 [Nasonia vitripennis] |
| 7912 | \| 1. CDNFEVTK \| \| --- \| \| 1. EVHCTQFAEGQADAR \| \| 1. IETMSESLSSDDAK \| \| 1. INVLDMNK \| \| 1. QGFYITGFTVNPDR \| \| 1. SQVIVYGK \| \| 1. TFDVVMTGK \| \| 1. VVITGLENDRKDDEIAYLR \| | 98.08 | Uncharacterized protein LOC105360086 | XP_011495179.1_PREDICTED: uncharacterized protein LOC105360086 [Ceratosolen solmsi marchali] |
| 7961 | \| 1. SFEEALQELKDK \| \| --- \| \| 1. VALKEEIAK \| \| 1. VIGNGIGNLEELKEK \| \| 1. VQDWLHHVQDVAK \| | 99.79 | Uncharacterized protein LOC100117458 | XP_001601691.5_uncharacterized protein LOC100117458 [Nasonia vitripennis] |
| 7966 | \| 1. GEPLDTYLNTAK \| \| --- \| \| 1. GEYGTVAMR \| \| 1. SHVGNDAVLYTTDGSYK \| \| 1. TVQASEPPTFEALK \| \| 1. VAMQLFDPQAR \| \| 1. VNFGNIDVEDFK \| \| 1. YAEEYLNQLLTR \| \| 1. YLPLPDIPTPIVSLK \| \| 1. YVSGSFHYFR \| | 99.02 | Beta-galactosidase-like isoform X1 | XP_001602984.2_beta-galactosidase-like isoform X1 [Nasonia vitripennis] |
| 8029 | \| 1. AVAWWVGR \| \| --- \| \| 1. DYPAPLDVLPYFNK \| \| 1. GCNDGESYDFALK \| \| 1. IVTDPNSEVVK \| \| 1. KADGTNVQNVLVTK \| \| 1. KLEALGIDSFK \| \| 1. KSVATGAPLNAPIWWLDPTNTYAHK \| \| 1. LAADTEWFGGPQLR \| \| 1. LSGTPALQENCAALTTCVK \| \| 1. LTETELPPK \| \| 1. NTAGDVHTVWWQGADAGAIDFTNPK \| \| 1. QFADQILK \| \| 1. VIDNPDGQITDCYK \| \| 1. VNDEYLLGEEILVAPVIEENAVTR \| \| 1. WKDGNTQVTWTGPILLR \| | 99.74 | Uncharacterized family 31 glucosidase KIAA1161 isoform X1 | XP_015190744.1_ PREDICTED: uncharacterized family 31 glucosidase KIAA1161 isoform X1 [Polistes dominula] |
| 8096 | \| 1. ATAAAAAAAATVPTGPTEQQPPHPHLLSR \| \| --- \| \| 1. LEGWATEIETK \| \| 1. LSLVTQEFNQIK \| \| 1. MSEVDTNLNYLLGR \| \| 1. NALTASILEYSGQIESR \| \| 1. QGLGAALDNIK \| \| 1. TSDTLEDLESR \| \| 1. VAGIEQLIAQK \| \| 1. VNQELHDLGK \| | 96.42 | Uncharacterized protein LOC105367713 | XP_011504784.1_PREDICTED: uncharacterized protein LOC105367713 [Ceratosolen solmsi marchali] |
| 8199 | \| 1. ADGYIMSYK \| \| --- \| \| 1. AQCLFNQPDIGTK \| \| 1. IPSAAEGQPK \| \| 1. KNHLEVTGGPNNK \| \| 1. LNIAGIILAK \| \| 1. NVLTFTPLK \| \| 1. TLVEGIIDNLAIR \| | 99.00 | Venom metalloproteinase 2-like isoform X2 | XP_016845742.1 _venom metalloproteinase 2-like isoform X2 [Nasonia vitripennis] |
| 8212 | \| 1. CFNVPQAGCLK \| \| --- \| \| 1. DIFDNTATLTGILR \| \| 1. GELQNLNFR \| \| 1. GNLLISPISLK \| \| 1. INSIIDPSTLQPDTK \| \| 1. LGIQNLFGK \| \| 1. LPFDSIDLR \| \| 1. LWLAQDVPILK \| \| 1. NALMILAEAAASQTR \| \| 1. NIAAYTINPFK \| \| 1. SNTELQTAIK \| \| 1. VSMIIMLPR \| \| 1. YSMFIILPSTPGK \| | 91.45 | Uncharacterized protein LOC100118367 | XP_008207605.3 _uncharacterized protein LOC100118367 [Nasonia vitripennis] |
| 8578 | \| 1. FNKDHVTAFK \| \| --- \| \| 1. LANQCGDSTDGCR \| \| 1. VAKGEESHGEPGPQPAAK \| \| 1. YSQIVWAK \| | 99.92 | Venom allergen 5-like | XP_001603551_venom allergen 5-like [Nasonia vitripennis] |
| 8693 | \| 1. ACIAETGVDIETLFK \| \| --- \| \| 1. DDQSLDEDAIR \| \| 1. IDKFIDACR \| \| 1. KTGVIKDDQSLDEDAIR \| \| 1. LTFGQVLAYK \| \| 1. SREQEVFDEKVK \| \| 1. TDVGKESCETGGIMLK \| \| 1. TGVIKDDQSLDEDAIRFK \| \| 1. VLQGLPEDKIDK \| | 97.96 | General odorant-binding protein 56d | XP_001601068.1_ general odorant-binding protein 56d [Nasonia vitripennis] |
| 9106 | \| 1. ALDNAVNNCKNEVGKNDCETAGK \| \| --- \| \| 1. AYMSANIPEK \| \| 1. EACIAESGVAESTLKEAK \| \| 1. KIGFMNSEGKLDEDITR \| | 99.54 | General odorant-binding protein 71 | CCD17840.1_putative odorant binding protein 71 [Nasonia vitripennis] |
| 9844 | \| 1. DFLGGLPLVGNSAETGK \| \| --- \| \| 1. DYIEFFGGNPQK \| \| 1. TLVPSLQTFVPIFGQSAK \| \| 1. YAEPPVGNR \| | 84.16 | Liver carboxylesterase 1 | XP_001605713.1_PREDICTED: liver carboxylesterase 1 [Nasonia vitripennis] |
| 9897 | \| 1. CFSDETLQKK \| \| --- \| \| 1. CNYDVVSKDEK \| \| 1. ENLIETHLIDAEGTSKR \| \| 1. FASLEVPDFK \| \| 1. FASSSANDAYTICDK \| \| 1. GNSVYTSQQR \| \| 1. GYLVFLPR \| \| 1. IYCYYPPPNHDR \| \| 1. LSVKPLVGNIYR \| \| 1. LTCIPSTHEIPPHLTFHK \| \| 1. SAIVTSSCTQYGLDGTEK \| \| 1. SGNVYLR \| \| 1. STEPSCEVTIKPYTEDR \| \| 1. VLSEYYDVK \| | 99.47 | Uncharacterized protein LOC100680003 | XP_008205889.1_uncharacterized protein LOC100680003 [Nasonia vitripennis] |
| 10216 | \| 1. DEPLSGLTNDPK \| \| --- \| \| 1. KIEPYTDYYIWHEGK \| \| 1. LLNDNVFAFSR \| \| 1. QAYYLHQFAPEQPDLNYR \| \| 1. SFKDSDDDGIGDLK \| \| 1. SMSLYQIYPR \| \| 1. VELYHTTALDGTGVGETIDSAK \| | 99.52 | Alpha-glucosidase | XP_001604662.2_alpha-glucosidase [Nasonia vitripennis] |
| 10521 | \| 1. CDNAVADLK \| \| --- \| \| 1. EYGCPIGTVFK \| \| 1. KSELLAGLQGSGETR \| \| 1. LYGIFADEKK \| \| 1. PSPARPAPAQQNQE \| \| 1. TCGNGLAFDASDSK \| \| 1. TQLEPPISTPHCTR \| \| 1. YQCSPGLAYDR \| \| 1. YYICMEGVAR \| | 99.98 | Uncharacterized protein LOC105362096 | XP_011497739.1_PREDICTED: uncharacterized protein LOC105362096 [Ceratosolen solmsi marchali] |
| 10716 | \| 1. DFDNLIAK \| \| --- \| \| 1. DQDEIYDLTSSWR \| \| 1. DSNGDGIGDLNGIASK \| \| 1. EMENVMTFWLDR \| \| 1. FAIVEAGSPIPLMMK \| \| 1. IKPYDNYYVWK \| \| 1. SGLKEQIVK \| \| 1. TGQLEISTDKGNNVLGVVR \| \| 1. TLNLAAEK \| \| 1. TWLPVHENYK \| \| 1. VFQTLAK \| | 99.91 | Alpha-glucosidase-like precursor | NP_0011989540.1_alpha-glucosidase-like precursor [Nasonia vitripennis] |
| 10799 | \| 1. GSKDEVPEDVRK \| \| --- \| \| 1. LSCALACIYENESSNR \| \| 1. SYHSLVK \| \| 1. YLEEIAKPK \| | 99.78 | Putative odorant binding protein 36 | CCD17805.1_putative odorant binding protein 36 [Nasonia vitripennis] |
| 11004 | \| 1. FTQEITANHICAGR \| \| --- \| \| 1. HVLTAAHCVYR \| \| 1. IHQGYVATTYK \| \| 1. LGEYDLR \| \| 1. TYQSCIAPGSK \| \| 1. YVVPGKPIQAPSK \| | 92.82 | Serine protease 67 precursor | NP_001166085.1_serine protease 67 precursor [Nasonia vitripennis] |
| 11185 | \| 1. CEPQLLAFDLNTDR \| \| --- \| \| 1. ELSQSNLEMVAQDSER \| \| 1. IAIDECNR \| \| 1. IFISTLGMEGVPATLNTVTNR \| \| 1. ILPMDVDKAPDGR \| \| 1. KDDCDSIQNVYR \| \| 1. LESPLFGPSDNAK \| \| 1. TAAIQSGAYDYSR \| \| 1. VQPEEEVFVLTNR \| \| 1. YIDYLWPSEAAR \| | 99.68 | Major royal jelly protein-like 7 precursor | NP_001154975.1_major royal jelly protein-like 7 precursor [Nasonia vitripennis] |
| 11199 | \| 1. DLFQQLVDLNNDAAR \| \| --- \| \| 1. GASIPWQDALQESIGESR \| \| 1. HLQTLGLANK \| \| 1. LKELYSGTKPPVLR \| \| 1. LVLLPYSIALDK \| \| 1. SEDDFDPGSK \| | 71.27 | Angiotensin-converting enzyme | XP_001607198.1_angiotensin-converting enzyme [Nasonia vitripennis] |
| 11592 | \| 1. AYSNEPFK \| \| --- \| \| 1. SSLHGILSR \| \| 1. VGEIPYQVSIR \| \| 1. YIDWIHK \| | 93.21 | Serine protease 72 precursor | NP_001166089.1_serine protease 72 precursor [Nasonia vitripennis] |
| 12519 | \| 1. GGVCLGADANR \| \| --- \| \| 1. IEVIDAGK \| \| 1. LSFGANKPGVFVEGGIHAR \| \| 1. WMEGGASSNPCQETFAGSAPFSEVETK \| | 99.32 | Zinc carboxypeptidase-like | XP_016838928.1_zinc carboxypeptidase-like [Nasonia vitripennis] |
| 14598 | \| 1. AANEQSDECEVAGAMGK \| \| --- \| \| 1. CLHACTMK \| \| 1. ELEELAK \| \| 1. HMPNADDVVK \| \| 1. IMDECAAEVGMTEHPHGPPDFSDQK \| | 99.89 | Putative odorant binding protein 8 | CCD17777.1_ putative odorant binding protein 8 [Nasonia vitripennis] |
| 18029 | \| 1. DLEISQVTLK \| \| --- \| \| 1. ESNELILHSK \| \| 1. LPEVAIPDTYNIR \| \| 1. SGSSINSDFQLEPER \| \| 1. YIGILNNNMR \| | 78.73 | Aminopeptidase N-like | XP_011497942.1_aminopeptidase N-like [Ceratosolen solmsi marchali] |

**Supplementary Table 4: *Torymus sinensis* venom proteins identified through the combined proteomic and transcriptomic approach.** In the table are reported: the contig number, the aminoacidic sequence of the peptides identified with by SDS-PAGE and LC-MS/MS, the percentage of signal peptide identified by Signal P 5.0 (<http://www.cbs.dtu.dk/services/SignalP/>), the protein name and the corresponding protein in NCBI database. NA = not annotated.

| **Contig** | **Contig Length (nt)** | **Signal Peptide Percentage (%)** | **Protein Name** | **Corresponding Protein in NCBI Database** |
| --- | --- | --- | --- | --- |
| 1029 | 1255 | 99.56 | Venom protein F precursor | NP_001155160.1_venom protein F precursor [Nasonia vitripennis] |
| 1427 | 1679 | 99.99 | Venom allergen 5-like | XP_001603551.4_venom allergen 5-like [Nasonia vitripennis] |
| 2092 | 831 | 99.26 | Venom protein L precursor | NP_001155029.1_venom protein L precursor [Nasonia vitripennis] |
| 2487 | 638 | 99.47 | Venom protein T precursor | NP_001155166.1_venom protein T precursor [Nasonia vitripennis] |
| 3070 | 3093 | 97.18 | Venom acid phosphatase Acph-1 | XP_001605452.1_venom acid phosphatase Acph-1 [Nasonia vitripennis] |
| 4647 | 769 | 99.21 | Venom protein V precursor | NP_001155041.1_venom protein V precursor [Nasonia vitripennis] |
| 5346 | 3125 | 99.04 | Cysteine-rich/KU venom protein precursor | NP_001154998.1_cysteine-rich/KU venom protein precursor [Nasonia vitripennis] |
| 7259 | 2073 | 99.25 | Venom protease | XP_016838644.1_venom protease [Nasonia vitripennis] |
| 7684 | 1825 | 99.47 | Venom carboxylesterase-6-like | XP_023246298.1_venom carboxylesterase-6-like [Copidosoma floridanum] |
| 7836 | 1663 | 81.03 | Venom acid phosphatase-like precursor | NP_001155147.1_venom acid phosphatase-like precursor [Nasonia vitripennis] |
| 9506 | 1853 | 99.35 | C1q-like venom protein precursor | NP_001155152.1_C1q-like venom protein precursor [Nasonia vitripennis] |
| 10580 | 902 | 96.81 | Venom protein N precursor | NP_001164349.1_venom protein N precursor [Nasonia vitripennis] |
| 11921 | 400 | 99.65 | Kazal type serine protease inhibitor-like venom protein 1 isoform X2 | XP_016843724.1_Kazal type serine protease inhibitor-like venom protein 1 isoform X2 [Nasonia vitripennis] |
| 12240 | 1707 | 95.83 | Venom carboxylesterase-6-like | XP_014211329.1_venom carboxylesterase-6-like [Copidosoma floridanum] |
| 12568 | 909 | 86.40 | U8-agatoxin-Ao1a-like isoform X2 | XP_032454492.1_U8-agatoxin-Ao1a-like isoform X2 [Nasonia vitripennis] |
| 12687 | 935 | 99.66 | Venom allergen 3 | XP_011165202.1_venom allergen 3 [Solenopsis invicta] |
| 19579 | 664 | 99.84 | Venom protein O precursor | NP_001155031.1_venom protein O precursor [Nasonia vitripennis] |
| 21875 | 428 | 99.96 | Cysteine-rich/pacifastin venom protein 2 precursor | NP_001154996.1_cysteine-rich/pacifastin venom protein 2 precursor [Nasonia vitripennis] |

**Supplementary Table 5: *Torymus sinensis* venom proteins, identified in the venom gland transcriptome through the “venom/toxin” keyword approach, provided by signal peptides.** The table contains the contig number, the length of the nucleotide sequence of the contig, the percentage of signal peptide identified by Signal P 5.0 (<http://www.cbs.dtu.dk/services/SignalP/>), the protein name and the corresponding protein in the NCBI database.

| **Contig** | **Contig Length (nt)** | **ORF** | **Protein Name** | **Corresponding Protein in NCBI Database** |
| --- | --- | --- | --- | --- |
| 472 | 530 | CLOSED | Venom protein Q precursor | NP_001155161.1_venom protein Q precursor [Nasonia vitripennis] |
| 1130 | 1587 | CLOSED | Plancitoxin-1 | XP_003424114.1_plancitoxin-1 [Nasonia vitripennis] |
| 1526 | 1158 | OPEN | Low-density lipoprotein receptor-like venom protein precursor | NP_001155040.1_low-density lipoprotein receptor-like venom protein precursor [Nasonia vitripennis] |
| 1535 | 1852 | NA | Kazal type serine protease inhibitor-like venom protein 2 precursor | NP_001164350.1_Kazal type serine protease inhibitor-like venom protein 2 precursor [Nasonia vitripennis] |
| 1953 | 1629 | OPEN | Endonuclease-like venom protein precursor | NP_001155087.1_endonuclease-like venom protein precursor [Nasonia vitripennis] |
| 3227 | 1212 | CLOSED | Venom protein D precursor | NP_001155171.1_venom protein D precursor [Nasonia vitripennis] |
| 4090 | 909 | CLOSED | Venom carboxylesterase-6 | XP_031779032.1_venom carboxylesterase-6 [Nasonia vitripennis] |
| 5910 | 803 | OPEN | Gamma-glutamyl cyclotransferase-like venom protein isoform 1 precursor | NP_001155144.1_gamma-glutamyl cyclotransferase-like venom protein isoform 1 precursor [Nasonia vitripennis] |
| 7070 | 4122 | CLOSED | Venom dipeptidyl peptidase 4 isoform X1 | XP_008202160.1_venom dipeptidyl peptidase 4 isoform X1 [Nasonia vitripennis] |
| 7321 | 1286 | CLOSED | Venom acid phosphatase Acph-1-like isoform X3 | XP_014215459.1_venom acid phosphatase Acph-1-like isoform X3 [Copidosoma floridanum] |
| 7343 | 1744 | CLOSED | Venom acid phosphatase Acph-1 isoform X1 | XP_031785785.1_venom acid phosphatase Acph-1 isoform X1 [Nasonia vitripennis] |
| 7940 | 749 | OPEN | Venom allergen 5-like | XP_001603551.4_venom allergen 5-like [Nasonia vitripennis] |
| 7893 | 1255 | OPEN | Venom serine carboxypeptidase-like | XP_001599671.2_venom serine carboxypeptidase-like [Nasonia vitripennis] |
| 8187 | 1436 | CLOSED | Venom acid phosphatase Acph-1-like | XP_001604473.1_venom acid phosphatase Acph-1-like [Nasonia vitripennis] |
| 8948 | 1327 | OPEN | Venom protease | XP_016838644.1_venom protease [Nasonia vitripennis] |
| 8691 | 1376 | CLOSED | Venom acid phosphatase Acph-1-like isoform X1 | XP_014215457.1_venom acid phosphatase Acph-1-like isoform X1 [Copidosoma floridanum] |
| 8857 | 750 | OPEN | Venom acid phosphatase Acph-1-like | XP_001603363.1_venom acid phosphatase Acph-1-like [Nasonia vitripennis] |
| 11671 | 2117 | CLOSED | Venom carboxylesterase-6 | XP_001605936.1_venom carboxylesterase-6 [Nasonia vitripennis] |
| 13041 | 1117 | OPEN | Venom acid phosphatase Acph-1 | XP_008217192.1_venom acid phosphatase Acph-1 [Nasonia vitripennis] |
| 13453 | 1274 | CLOSED | Venom acid phosphatase Acph-1-like | XP_014208120.1_venom acid phosphatase Acph-1-like [Copidosoma floridanum] |
| 12783 | 1069 | CLOSED | Venom allergen 3-like isoform X1 | XP_014222132.1_venom allergen 3-like isoform X1 [Trichogramma pretiosum] |
| 14872 | 333 | OPEN | Venom acid phosphatase Acph-1-like | XP_014231272.1_venom acid phosphatase Acph-1-like [Trichogramma pretiosum] |
| 14873 | 1210 | CLOSED | Venom acid phosphatase Acph-1-like | XP_001607714.2_venom acid phosphatase Acph-1-like [Nasonia vitripennis] |
| 15076 | 1441 | CLOSED | Venom serine carboxypeptidase | XP_003704314.1_PREDICTED: venom serine carboxypeptidase [Megachile rotundata] |
| 15429 | 218 | OPEN | Venom carboxylesterase-6 | XP_014217874.1_venom carboxylesterase-6 [Nasonia vitripennis] |
| 16269 | 964 | OPEN | Venom acid phosphatase Acph-1-like | XP_001607711.1_venom acid phosphatase Acph-1-like [Nasonia vitripennis] |
| 16876 | 779 | OPEN | Venom acid phosphatase | ACA60733.1_venom acid phosphatase [Pteromalus puparum] |
| 17081 | 1774 | OPEN | Venom laccase isoform X1 | XP_016840302.1_venom laccase isoform X1 [Nasonia vitripennis] |
| 18015 | 562 | OPEN | Venom acid phosphatase Acph-1-like | XP_014213880.1_venom acid phosphatase Acph-1-like [Copidosoma floridanum] |
| 18733 | 1010 | OPEN | Venom acid phosphatase Acph-1 | XP_001605515.2_venom acid phosphatase Acph-1 [Nasonia vitripennis] |
| 18844 | 314 | OPEN | Venom acid phosphatase Acph-1-like | XP_001605977.1_venom acid phosphatase Acph-1-like [Nasonia vitripennis] |
| 19895 | 316 | OPEN | Venom carboxylesterase-6 isoform X1 | XP_001599255.3_venom carboxylesterase-6 isoform X1 [Nasonia vitripennis] |
| 20332 | 292 | OPEN | Venom acid phosphatase Acph-1-like | XP_016915476.1_venom acid phosphatase Acph-1-like [Apis cerana] |
| 21975 | 210 | NA | Venom carboxylesterase-6 | XP_00159980_venom carboxylesterase-6 [Nasonia vitripennis] |

**Supplementary Table 6: *Torymus sinensis* venom proteins identified in venom gland transcriptome through the “venom/toxin” keyword approach, devoid of signal peptide.** The table contains the contig number, the length of the nucleotide sequence of the contig, indications concerning the sequence at 5 ' end (open, closed or not available (NA) as the ORF was too small to obtain information about signal peptide), the protein name and the corresponding protein in NCBI database.

To further support the belonging of the 52 putative proteins annotated using “venom” as key, to the mixture of the *T. sinensis* venom components, their nucleotide sequences were translated using Expasy-translate tool software (https://web.expasy.org/translate/) and the corresponding protein sequences were compared to those of *N. vitripennis*, reported in de Graaf et al.^30^, obtaining the following matches, also reported in Supplementary tables 5, 6, 7:

- 10 *T. sinensis* contigs match with 7 of the *N. vitripennis* proteins found by bioinformatic approach (Table S1^30^);

- 34 *T. sinensis* contigs match with 22 of the *N. vitripennis* venom proteins found by proteomic approach and provided with signal peptide (Table S2^30^);

- 2 *T. sinensis* contigs match with 1 of the *N. vitripennis* protein found by proteomic approach (Table S3^30^);

Three *T. sinensis* proteins (c3591, c14873 and c16269) match with 2 of the *N. vitripennis* venom proteins identified by both a bioinformatic approach and protein discovered by a proteomic approach, as they have the same accession number.

9 *T. sinensis* contigs don’t match with any of the *N. vitripennis* venom proteins, but they resulted very similar to other venom components, such as carboxylesterase, Kazal type serine protease inhibitor, acid phosphatase and carboxypeptidase (Supplementary Table 8).

| **Contig** | ***Torymus sinensis* protein** | ***Nasonia vitripennis* protein^30^** | **Query cover** | **E-value** | **Identity** |
| --- | --- | --- | --- | --- | --- |
| 1427 | Venom allergen 5-like | [NP_001155154.1 antigen 5-like protein 1 precursor [Nasonia vitripennis]](https://blast.ncbi.nlm.nih.gov/Blast.cgi#alnHdr_Query_28086) | 98% | 2,00E-98 | 56.14% |
| 5346 | Cysteine-rich/KU venom protein precursor | [NP_001154998.1 cysteine-rich/KU venom protein precursor [Nasonia vitripennis]](https://blast.ncbi.nlm.nih.gov/Blast.cgi#alnHdr_Query_28092) | 100% | 0.0 | 77.50% |
| 7940 | Venom allergen 5-like | [NP_001155154.1 antigen 5-like protein 1 precursor [Nasonia vitripennis]](https://blast.ncbi.nlm.nih.gov/Blast.cgi#alnHdr_Query_28086) | 99% | 3,00E-109 | 61.74% |
| 9506 | C1q-like venom protein precursor | [NP_001155152.1 C1q-like venom protein precursor [Nasonia vitripennis]](https://blast.ncbi.nlm.nih.gov/Blast.cgi#alnHdr_Query_28089) | 100% | 1,00E-92 | 84.38% |
| 12783 | Venom allergen 3-like isoform X1 | [NP_001155154.1 antigen 5-like protein 1 precursor [Nasonia vitripennis]](https://blast.ncbi.nlm.nih.gov/Blast.cgi#alnHdr_Query_28086) | 89% | 6,00E-84 | 59.89% |
| 14873 | Venom acid phosphatase Acph-1-like | [NP_001155146.1 venom acid phosphatase-like precursor [Nasonia vitripennis]](https://blast.ncbi.nlm.nih.gov/Blast.cgi#alnHdr_Query_28084) | 94% | 2,00E-51 | 32.56% |
| 16269 | Venom acid phosphatase Acph-1-like | [NP_001155146.1 venom acid phosphatase-like precursor [Nasonia vitripennis]](https://blast.ncbi.nlm.nih.gov/Blast.cgi#alnHdr_Query_28084) | 81% | 2,00E-18 | 28.78% |
| 17081 | Venom laccase isoform X1 | [NP_001155158.1 venom laccase precursor [Nasonia vitripennis]](https://blast.ncbi.nlm.nih.gov/Blast.cgi#alnHdr_Query_28098) | 98% | 0.0 | 68.52% |
| 20332 | Venom acid phosphatase Acph-1-like | [NP_001155146.1 venom acid phosphatase-like precursor [Nasonia vitripennis]](https://blast.ncbi.nlm.nih.gov/Blast.cgi#alnHdr_Query_28084) | 100% | 3,00E-04 | 40.54% |
| 21875 | Cysteine-rich/pacifastin venom protein 2 precursor | [NP_001154996.1 cysteine-rich/pacifastin venom protein 2 precursor [Nasonia vitripennis]](https://blast.ncbi.nlm.nih.gov/Blast.cgi#alnHdr_Query_28094) | 97% | 2,00E-36 | 53.45% |

**Supplementary Table 7: *Torymus sinensis* venom proteins identified through the transcriptomic approach aligned with *Nasonia vitripennis* proteins in de Graaf et al.^30^ (Table S1. Venom proteins discovered by a bioinformatic approach).** In the table are reported: the contig number, the protein name, the *N. vitripennis* protein and data concerning the alignment of the two proteins (query cover, E-value and identity). The alignments were performed with the software BLASTp (<https://blast.ncbi.nlm.nih.gov/Blast.cgi?PAGE=Proteins>).

| **Contig** | ***Torymus sinensis* protein** | ***Nasonia vitripennis* protein^30^** | **Query cover** | **E-value** | **Identity** |
| --- | --- | --- | --- | --- | --- |
| 472 | Venom protein Q precursor | [NP_001155161.1 venom protein Q precursor [Nasonia vitripennis]](https://blast.ncbi.nlm.nih.gov/Blast.cgi#alnHdr_Query_28074) | 91% | 5,00E-24 | 46.23% |
| 1029 | Venom protein F precursor | [NP_001155160.1 venom protein F precursor [Nasonia vitripennis]](https://blast.ncbi.nlm.nih.gov/Blast.cgi#alnHdr_Query_28063) | 82% | 5,00E-117 | 65.35% |
| 1535 | Kazal type serine protease inhibitor-like venom protein 2 precursor | [NP_001164350.1 Kazal type serine protease inhibitor-like venom protein 2 precursor [Nasonia vitripennis]](https://blast.ncbi.nlm.nih.gov/Blast.cgi#alnHdr_Query_28042) | 92% | 8,00E-14 | 40.74% |
| 2092 | Venom protein L precursor | [NP_001155029.1 venom protein L precursor [Nasonia vitripennis]](https://blast.ncbi.nlm.nih.gov/Blast.cgi#alnHdr_Query_28069) | 73% | 7,00E-11 | 31.33% |
| 2487 | Venom protein T precursor | [NP_001155166.1 venom protein T precursor [Nasonia vitripennis]](https://blast.ncbi.nlm.nih.gov/Blast.cgi#alnHdr_Query_28077) | 83% | 2,00E-15 | 30.00% |
| 3070 | Venom acid phosphatase Acph-1 | [NP_001155147.1 venom acid phosphatase-like precursor [Nasonia vitripennis]](https://blast.ncbi.nlm.nih.gov/Blast.cgi#alnHdr_Query_28023) | 86% | 3,00E-46 | 32.33% |
| 3227 | Venom protein D precursor | [XP_016838723.1 venom protein D isoform X1 [Nasonia vitripennis]](https://blast.ncbi.nlm.nih.gov/Blast.cgi#alnHdr_Query_28061) | 94% | 2,00E-60 | 45.50% |
| 4090 | Venom carboxylesterase-6 | [NP_001155148.1 carboxylesterase clade B, member 2 precursor [Nasonia vitripennis]](https://blast.ncbi.nlm.nih.gov/Blast.cgi#alnHdr_Query_28024) | 43% | 0.41 | 25.00% |
| 4647 | Venom protein V precursor | [NP_001155041.1 venom protein V precursor [Nasonia vitripennis]](https://blast.ncbi.nlm.nih.gov/Blast.cgi#alnHdr_Query_28079) | 41% | 8,00E-08 | 33.67% |
| 5910 | Gamma-glutamyl cyclotransferase-like venom protein isoform 1 precursor | [NP_001155144.1 gamma-glutamyl cyclotransferase-like venom protein isoform 1 precursor [Nasonia vitripennis]](https://blast.ncbi.nlm.nih.gov/Blast.cgi#alnHdr_Query_28036) | 97% | 7,00E-95 | 71.01% |
| 7070 | Venom dipeptidyl peptidase 4 isoform X1 | [XP_001599462.2 PREDICTED: venom dipeptidyl peptidase 4 isoform 1 [Nasonia vitripennis]](https://blast.ncbi.nlm.nih.gov/Blast.cgi#alnHdr_Query_28032) | 89% | 0.0 | 39.46% |
| 7259 | Venom protease | [NP_001155043.1 serine protease 22 precursor [Nasonia vitripennis]](https://blast.ncbi.nlm.nih.gov/Blast.cgi#alnHdr_Query_28058) | 90% | 1,00E-57 | 36.12% |
| 7321 | Venom acid phosphatase Acph-1-like isoform X3 | [NP_001155147.1 venom acid phosphatase-like precursor [Nasonia vitripennis]](https://blast.ncbi.nlm.nih.gov/Blast.cgi#alnHdr_Query_28023) | 92% | 4,00E-43 | 32.19% |
| 7343 | Venom acid phosphatase Acph-1 isoform X1 | [NP_001155147.1 venom acid phosphatase-like precursor [Nasonia vitripennis]](https://blast.ncbi.nlm.nih.gov/Blast.cgi#alnHdr_Query_28023) | 83% | 4,00E-47 | 37.67% |
| 7836 | Venom acid phosphatase-like precursor | [NP_001155147.1 venom acid phosphatase-like precursor [Nasonia vitripennis]](https://blast.ncbi.nlm.nih.gov/Blast.cgi#alnHdr_Query_28023) | 100% | 3,00E-168 | 57.74% |
| 7684 | Venom carboxylesterase-6-like | [NP_001155148.1 carboxylesterase clade B, member 2 precursor [Nasonia vitripennis]](https://blast.ncbi.nlm.nih.gov/Blast.cgi#alnHdr_Query_28024) | 93% | 8,00E-136 | 42.45% |
| 7893 | Venom serine carboxypeptidase-like | [NP_001155148.1 carboxylesterase clade B, member 2 precursor [Nasonia vitripennis]](https://blast.ncbi.nlm.nih.gov/Blast.cgi#alnHdr_Query_28024) | 16% | 1.9 | 27.14% |
| 8187 | Venom acid phosphatase Acph-1-like | [NP_001155147.1 venom acid phosphatase-like precursor [Nasonia vitripennis]](https://blast.ncbi.nlm.nih.gov/Blast.cgi#alnHdr_Query_28023) | 93% | 4,00E-59 | 46.35% |
| 8691 | Venom acid phosphatase Acph-1-like isoform X1 | [NP_001155147.1 venom acid phosphatase-like precursor [Nasonia vitripennis]](https://blast.ncbi.nlm.nih.gov/Blast.cgi#alnHdr_Query_28023) | 86% | 2,00E-43 | 32.66% |
| 8857 | Venom acid phosphatase Acph-1-like | [NP_001155147.1 venom acid phosphatase-like precursor [Nasonia vitripennis]](https://blast.ncbi.nlm.nih.gov/Blast.cgi#alnHdr_Query_28023) | 98% | 1,00E-18 | 29.15% |
| 8948 | Venom protease | [NP_001155077.1 serine protease 16 precursor [Nasonia vitripennis]](https://blast.ncbi.nlm.nih.gov/Blast.cgi#alnHdr_Query_28059) | 54% | 2,00E-15 | 36.51% |
| 10580 | Venom protein N precursor | [NP_001164349.1 venom protein N precursor [Nasonia vitripennis]](https://blast.ncbi.nlm.nih.gov/Blast.cgi#alnHdr_Query_28071) | 95% | 1,00E-54 | 54.29% |
| 11671 | Venom carboxylesterase-6 | [NP_001155148.1 carboxylesterase clade B, member 2 precursor [Nasonia vitripennis]](https://blast.ncbi.nlm.nih.gov/Blast.cgi#alnHdr_Query_28024) | 90% | 6,00E-59 | 29.00% |
| 11921 | Kazal type serine protease inhibitor-like venom protein 1 isoform X2 | [NP_001154995.1 Kazal type serine protease inhibitor-like venom protein 1 precursor [Nasonia vitripennis]](https://blast.ncbi.nlm.nih.gov/Blast.cgi#alnHdr_Query_28041) | 98% | 1,00E-27 | 66.67% |
| 12240 | Venom carboxylesterase-6-like | [NP_001155148.1 carboxylesterase clade B, member 2 precursor [Nasonia vitripennis]](https://blast.ncbi.nlm.nih.gov/Blast.cgi#alnHdr_Query_28024) | 95% | 2,00E-71 | 30.77% |
| 13041 | Venom acid phosphatase Acph-1 | [NP_001155147.1 venom acid phosphatase-like precursor [Nasonia vitripennis]](https://blast.ncbi.nlm.nih.gov/Blast.cgi#alnHdr_Query_28023) | 91% | 1,00E-56 | 37.58% |
| 13453 | Venom acid phosphatase Acph-1-like | [NP_001155147.1 venom acid phosphatase-like precursor [Nasonia vitripennis]](https://blast.ncbi.nlm.nih.gov/Blast.cgi#alnHdr_Query_28023) | 93% | 8,00E-42 | 31.02% |
| 15429 | Venom carboxylesterase-6 | [NP_001155148.1 carboxylesterase clade B, member 2 precursor [Nasonia vitripennis]](https://blast.ncbi.nlm.nih.gov/Blast.cgi#alnHdr_Query_28024) | 88% | 2,00E-14 | 48.21% |
| 16876 | Venom acid phosphatase | [NP_001155147.1 venom acid phosphatase-like precursor [Nasonia vitripennis]](https://blast.ncbi.nlm.nih.gov/Blast.cgi#alnHdr_Query_28023) | 100% | 3,00E-109 | 55.96% |
| 18015 | Venom acid phosphatase Acph-1-like | [NP_001155147.1 venom acid phosphatase-like precursor [Nasonia vitripennis]](https://blast.ncbi.nlm.nih.gov/Blast.cgi#alnHdr_Query_28023) | 81% | 2,00E-18 | 35.59% |
| 18733 | Venom acid phosphatase Acph-1 | [NP_001155147.1 venom acid phosphatase-like precursor [Nasonia vitripennis]](https://blast.ncbi.nlm.nih.gov/Blast.cgi#alnHdr_Query_28023) | 94% | 1,00E-28 | 26.43% |
| 18844 | Venom acid phosphatase Acph-1-like | [NP_001155147.1 venom acid phosphatase-like precursor [Nasonia vitripennis]](https://blast.ncbi.nlm.nih.gov/Blast.cgi#alnHdr_Query_28023) | 100% | 4,00E-17 | 44.29% |
| 19895 | Venom carboxylesterase-6 isoform X1 | [NP_001155148.1 carboxylesterase clade B, member 2 precursor [Nasonia vitripennis]](https://blast.ncbi.nlm.nih.gov/Blast.cgi#alnHdr_Query_28024) | 100% | 2,00E-19 | 55.17% |
| 19579 | Venom protein O precursor | [NP_001155031.1 venom protein O precursor [Nasonia vitripennis]](https://blast.ncbi.nlm.nih.gov/Blast.cgi#alnHdr_Query_28072) | 77% | 4,00E-38 | 51.49% |

**Supplementary Table 8: *Torymus sinensis* venom proteins identified through the transcriptomic approach aligned with *Nasonia vitripennis* proteins in de Graaf et al.^30^ (Table S2. Venom proteins discovered by a proteomic approach).** In the table are reported: the contig number, the protein name, the *N. vitripennis* protein and data concerning the alignment of the two proteins (query cover, E-value and identity). The alignments were performed with the software BLASTp (<https://blast.ncbi.nlm.nih.gov/Blast.cgi?PAGE=Proteins>).

| **Contig** | ***Torymus sinensis* protein** | ***Nasonia vitripennis* protein^30^** | **Query cover** | **E-value** | **Identity** |
| --- | --- | --- | --- | --- | --- |
| 14873 | Venom acid phosphatase Acph-1-like | [NP_001155146.1 venom acid phosphatase-like precursor [Nasonia vitripennis]](https://blast.ncbi.nlm.nih.gov/Blast.cgi#alnHdr_Query_28084) | 94% | 2,00E-51 | 32.56% |
| 16269 | Venom acid phosphatase Acph-1-like | [NP_001155146.1 venom acid phosphatase-like precursor [Nasonia vitripennis]](https://blast.ncbi.nlm.nih.gov/Blast.cgi#alnHdr_Query_28084) | 81% | 2,00E-18 | 28.78% |

**Supplementary Table 9: *Torymus sinensis* venom proteins identified through the transcriptomic approach aligned with *Nasonia vitripennis* proteins in de Graaf et al.^30^ (Table S3. Non-secretory proteins discovered by a proteomic approach).** In the table are reported: the contig number, the protein name, the *N. vitripennis* protein and data concerning the alignment of the two proteins (query cover, E-value and identity). The alignments were performed with the software BLASTp (<https://blast.ncbi.nlm.nih.gov/Blast.cgi?PAGE=Proteins>).

| **Contig** | **Protein name** | **Correspondent Protein in NCBI Database** |
| --- | --- | --- |
| 1130 | Plancitoxin-1 | XP_003424114.1_plancitoxin-1 [Nasonia vitripennis] |
| 1526 | Low-density lipoprotein receptor-like venom protein precursor | NP_001155040.1_low-density lipoprotein receptor-like venom protein precursor [Nasonia vitripennis] |
| 1535 | Kazal type serine protease inhibitor-like venom protein 2 precursor | NP_001164350.1_Kazal type serine protease inhibitor-like venom protein 2 precursor [Nasonia vitripennis] |
| 1953 | Endonuclease-like venom protein precursor | NP_001155087.1_endonuclease-like venom protein precursor [Nasonia vitripennis] |
| 12568 | U8-agatoxin-Ao1a-like isoform X2 | XP_032454492.1_U8-agatoxin-Ao1a-like isoform X2 [Nasonia vitripennis] |
| 12687 | Venom allergen 3 | XP_011165202.1_venom allergen 3 [Solenopsis invicta] |
| 14872 | Venom acid phosphatase Acph-1-like | XP_014231272.1_venom acid phosphatase Acph-1-like [Trichogramma pretiosum] |
| 15076 | Venom serine carboxypeptidase | XP_003704314.1_PREDICTED: venom serine carboxypeptidase [Megachile rotundata] |
| 21975 | Venom carboxylesterase-6 | XP_00159980_venom carboxylesterase-6 [Nasonia vitripennis] |

**Supplementary Table 10: *Torymus sinensis* venom proteins identified through the transcriptomic approach that don’t match with *N. vitripennis* proteins in de Graaf et al. ^30^.** In the table are reported: the contig number, the protein name and the corresponding protein in NCBI database

| **Contig** | **Gene name** | **Primer 5’ 3’** |
| --- | --- | --- |
| 2629 | Glyceraldehyde 3-phosphate dehydrogenase (GAPDH) | **F:** ATGTCGAAACGGAACTGAGG  **R:** TTGTCGAATCACGGAAATGA |
| 21759 | Beta tubulin | **F:** ACACTTTGGGCGAAGGTACA  **R:** ACGCAAGGAAGCAGAGTCAT |
| 4 | Serine protease 33 isoform X2 | **F:** ACTTACGATCGCCTGGTGAC  **R:** CGTGCTCTTAGAACGGGAAG |
| 428 | Lysosomal aspartic protease-like | **F:** CCATGAAGCCACTCAAGCAG  **R:** TCGCTGACACTGGAACTTCT |
| 1650 | Peptidyl-prolyl cis-trans isomerase 5 | **F:** TTCCATCTCCCTTGGTGAAG  **R:** CAAGAAGGGACCCAAAGTCA |
| 293 | Cathepsin L | **F:** TCCTTGTTACGGGCCATCTT  **R:** CCTGTTTCCGTTGCCATTGA |
| 2095 | Carboxylesterase clade B, member 2 precursor | **F:** GCCGGCGTTTCGTAAAGTAT  **R:** GGTGGTGTTGCTGTTGACTT |
| 1194 | Ferritin precursor | **F:** CGCTTGCTGTGCTACTACTG  **R:** CATCAGGCATCTCTCTCCGT |
| 1971 | Chymotrypsin-1-like | **F:** TACACGGTCCTGCTTCCTTT  **R:** GCCATTAAGTCGTCCAGCTG |
| 495 | Pancreatic triacylglycerol lipase | **F:** TCTGTGCCCAATGAAACTGC  **R:** CAACGGTGGTCAAGTACAGC |
| 3120 | Venom allergen 5-like | **F:** TGCCAGTTCCTCATCCCATT  **R:** CGGACCATCAAAAGCATGCT |
| 13330 | Alpha-glucosidase-like | **F:** GGGAGTGTGGAATGTGTGGA  **R:** ACATCGACAAAACAACGGCT |

**Supplementary Table 11: Primers used for qRT-PCR of *Torymus sinensis* selected venom components.** F: forward, R: reverse.
